# Phase I study of pembrolizumab in combination with ibrutinib for the treatment of unresectable or metastatic melanoma

**DOI:** 10.3389/fimmu.2025.1491448

**Published:** 2025-02-04

**Authors:** Yuan Yao, Yiyi Yan, Vera J. Suman, Allan B. Dietz, Courtney L. Erskine, Anastasios Dimou, Svetomir N. Markovic, Robert R. McWilliams, Heather N. Montane, Matthew S. Block

**Affiliations:** ^1^ Department of Oncology, Mayo Clinic, Rochester, MN, United States; ^2^ Division of Hematology and Oncology, Department of Medicine, Mayo Clinic, Jacksonville, FL, United States; ^3^ Department of Health Sciences, Division of Quantitative Health Sciences, Mayo Clinic, Rochester, MN, United States; ^4^ Department of Laboratory Medicine and Pathology, Mayo Clinic, Rochester, MN, United States; ^5^ Department of Immunology, Mayo Clinic, Rochester, MN, United States

**Keywords:** melanoma, ibrutinib, pembrolizumab, Th2 (type-2) immune responses, Th1 immune responses, immunotherapy

## Abstract

**Background:**

Immune checkpoint inhibitors (ICIs) have been transformative in the treatment of patients with metastatic melanoma, but primary and secondary resistance to ICI treatment is common. One key mechanism for ICI resistance is the skewing of the immune response from a cytotoxic (Th1) to a chronic inflammatory (Th2) profile. The small molecule ibrutinib is a dual-target agent that inhibits Bruton’s Tyrosine Kinase (BTK) and Interleukin-2-inducible T-cell Kinase (ITK), a key regulator of Th2 immunity. Therefore, combining ibrutinib and pembrolizumab could potentially induce an increase in Th1 immune polarity in melanoma patients. We hypothesize that the combination would be well-tolerated and might result in clinical benefit for patients with metastatic melanoma. The primary aim of this phase I study was to evaluate the safety, tolerability, and determine the maximum tolerated dose (MTD) of ibrutinib in combination with pembrolizumab in patients with metastatic melanoma.

**Methods:**

A 3 + 3 phase I clinical trial was conducted in patients with unresectable Stage III or metastatic melanoma (stage IV) not amenable to local therapy. Pembrolizumab (200 mg/kg every 3 weeks) was combined with ibrutinib, administered orally at the dose assigned at the time of registration (140 mg daily, 280 mg daily, and 420 mg daily). Patients were treated until disease progression, intolerability, or patient decision to discontinue. Blood samples were collected after each cycle of treatment for immunophenotyping and Th1/Th2 polarity assessment based on immune response markers.

**Results:**

Between January 31, 2017 and January 9, 2023, 17 patients were enrolled. The MTD of ibrutinib in combination with pembrolizumab was determined to be 420 mg daily. The adverse events leading to discontinuation included: grade 4 ALT and AST increase (1 pt, DL0); grade 4 ALT increase with grade 3 AST increase (1 pt, DL1); and grade 3 hyponatremia, hypoxia, and maculo-papular rash (1 pt, DL1). Three of the 16 patients treated had objective responses (2 partial responses, 1 complete response) lasting over 8 months. The median progression-free survival was 3 months, and median and overall survival was 1.8 years. The combination treatment did not result in consistent increase in Th1 immune polarity.

**Conclusion:**

In conclusion, the maximum tolerated dose of ibrutinib in combination with pembrolizumab in patients with advanced or metastatic melanoma was established at 420 mg by mouth once daily. The combination was well-tolerated but did not result in a consistent increase in Th1 immune polarity; further investigation is needed to assess the relative clinical efficacy of this approach. (Funded by Pharmacyclics; ClinicalTrials.gov number: NCT03021460)

**Clinical trial registration:**

www.clinicaltrials.gov, identifier NCT03021460.

## Introduction

Melanoma is one of the most immunogenic malignancies. Over the last decade, our understanding of immune checkpoints and the effects of immune checkpoint inhibition has paved the way for the advances made in cancer immunotherapy, including in metastatic melanoma. Dysregulation of immune checkpoints in tumor cells is an important mechanism of tumor immune escape, and accumulating evidence shows that escape can be overcome by immune checkpoint blockade. Blockade of cytotoxic T-lymphocyte antigen 4 (CTLA-4) and programmed death (PD-1) receptors have shown durable anti-melanoma effects (1, 2) and have been transformative in the care of patients with advanced or metastatic melanoma. Specifically, the human IgG4 PD-1 blocking antibody pembrolizumab has shown a high rate of durable clinical responses, with an objective response rate (ORR) of 26% and median progression-free survival of 22 weeks [95% CI: 12-36 weeks (2)]. The addition of anti-CTLA-4 or anti-LAG3 antibodies to anti-PD-1 therapy has resulted in modest improvements in ORR, but these gains have come at the expense of increased toxicity (3, 4).

However, despite these promising data, the majority of melanoma patients either exhibit primary or secondary resistance to pembrolizumab and other PD-1/PD-L1-targeting monoclonal antibodies. Proposed mechanisms of PD-1 blockade resistance include additional mechanisms of immunosuppression in the tumor microenvironment (5) and derangements in systemic immune competence (6). Specifically, increased regulatory T-cells (Treg) and Th2 cells were demonstrated in patients with metastatic melanoma (7, 8). Successful tumor-immune surveillance is mediated by Th1 cells, which are associated with tumor–specific CD8+ T-cells. In contrast, a Th2-dominant immune response—with production of “chronic inflammation” cytokines such as interleukin (IL-) 4, IL-5, IL-10, and IL-13—is linked to malignancy progression and metastasis. Our previous study showed that advanced melanoma patients have elevated Th2 cytokines that incapacitate anti-tumor immune responses (9). Such dysregulation can serve as a barrier to successful immunotherapy.

Ibrutinib is a first-in-class, potent, orally administered inhibitor of Bruton’s Tyrosine Kinase (BTK) (10). Activity of ibrutinib was demonstrated in chronic lymphocytic leukemia (CLL)/small lymphocytic lymphoma (SLL), diffuse large B-cell lymphoma (DLBCL), mantle cell lymphoma (MCL), follicular lymphoma (FL), Waldenstrom’s macroglobulinemia (WM), marginal zone lymphoma (MZL), and multiple myeloma (MM) (11–16). Recent studies revealed that in addition to inhibiting BTK, ibrutinib irreversibly binds to Interleukin-2-inducible T-cell Kinase (ITK). Given that ITK plays a critical role in Th2 differentiation, it has been shown that ibrutinib can skew immune responses from a Th2-dominant pattern to a Th1-dominant pattern (17).

Therefore, the addition of ibrutinib, by skewing T-cell responses towards an anti-tumor Th1 phenotype, might synergize with pembrolizumab to improve the efficacy of PD-1-targeting immune checkpoint inhibitor therapy. Based on currently available data, ibrutinib has an acceptable safety profile as monotherapy and combined with certain chemoimmunotherapies or immunotherapies. As such, we designed a phase I/II clinical trial to determine the safety, tolerability, and maximum tolerated dose of ibrutinib in combination with pembrolizumab in patients with advanced melanoma, and to estimate the overall response rate for this patient population treated at the maximum tolerated dose of ibrutinib in combination with pembrolizumab. We then tested for alterations in the balance between Th1 and Th2 immune responses using plasma and peripheral blood cells from serial patient biospecimens.

## Methods

### Study design and patients

A Phase II clinical trial was initially designed to assess the anti-tumor activity and safety profile of pembrolizumab at 200 mg/kg every 3 weeks with ibrutinib 560 mg daily. Enrollment to the trial was temporarily closed after 2 patients accrued both developed grade 3 maculopapular rash. Following discussion with the Data and Safety Monitoring Board (DSMB) and approval by Mayo Clinic Institutional Review Board (IRB), the study was converted to a 3 + 3 phase I clinical trial with an expansion cohort to determine the maximum tolerated dose (MTD) of ibrutinib in combination with pembrolizumab.

A 3 + 3 phase I clinical trial with an expansion cohort of 6 patients at the MTD (**Supplementary Materials**: Protocol) was conducted to evaluate the safety, tolerability, and maximum tolerated dose of ibrutinib in combination with pembrolizumab in patients with unresectable stage III or metastatic melanoma (stage IV) not amenable to local therapy, while exploring the preliminary antitumor activity of the combination. This trial had a pre-registration component where a tumor biopsy was undertaken for research purposes.

The first cycle of treatment administration was designed to evaluate changes in immune parameters after 7 days of ibrutinib from pre-ibrutinib levels and then changes from post-ibrutinib levels to levels after 21 days of pembrolizumab. Thus cycle 1 was 28 days with ibrutinib administered orally at dose assigned at registration on days 1-7 followed by 200 mg pembrolizumab administered intravenously on day 8. Subsequent cycles were 21 days long, with pembrolizumab 200 mg administered intravenously on day 1 and ibrutinib administered daily.

Dose escalation began with dose level 0 (DL0): ibrutinib 280 mg daily. If at most 1 of 3 to 6 patients treated at DL0 developed a dose-limiting toxicity (DLT), then the next dose level to be tested was DL1: ibrutinib 420 mg daily. If 2 or more of the 3 to 6 patients treated at DL0 developed a DLT then the next dose level to be tested was DL -1: ibrutinib 140 mg daily. No dose escalation was permitted in an individual patient.

DLTs were defined as: grade 4 neutrophil count decrease, grade 4 anemia, PLT < 25,000, serum creatinine ≥2 times baseline, ≥ grade 2 neurosensory or neuromotor toxicity, grade 3 rash or fever despite maximal supportive treatment(s), and any other non-hematologic toxicity of grade 3 or higher per NCI Common Terminology Criteria for Adverse Events version 4.0 (CTCAE v. 4.0) occurring from the start of treatment administration on Day 1 of Cycle 1 up to the start of treatment administration on Day 1 of Cycle 2 (28 +/- 3 days).

Eligible patients were at least 18 years of age; had received a diagnosis of unresectable stage III or IV melanoma with at least 1 measurable non-nodal lesion; had an Eastern Cooperative Oncology Group performance status of 0 (asymptomatic), 1 (ambulatory but restricted in strenuous activity) or 2 (capable of self-care but unable to perform work activities) (18); and had adequate organ function. If patients received prior anti-PD-1 or anti-PD-L1 therapy, eligibility criteria include disease progression within 6 months after the last dose of anti-PD-1/anti-PD-L1 treatment in the adjuvant or metastatic setting.

Exclusion criteria include uveal melanoma, prior chemotherapy, immunotherapy (including monoclonal antibody), or radioactive therapy within 28 days prior to registration, history of severe autoimmune disease or organ transplant, human immunodeficiency virus infection, hepatitis B or C and central nervous system metastases. For a complete list of inclusion and exclusion criteria, please refer to **Supplementary Materials**: Protocol section 3.0.

### Monitoring

After each cycle of treatment, patients underwent a physical exam, assessment of performance status, blood chemistries toxicity assessments, and research blood draws (baseline; cycle 1, day 8; and day 1 of subsequent cycles). Does modification guidelines are provided in **Supplementary Materials**: Protocol section 8.0. The number of ibrutinib dose reductions that were allowed were based on starting dose of ibrutinib and assuring that no patient received less than 140 mg daily. Disease status was radiographically evaluated using Response Evaluation Criteria in Solid Tumors (RECIST) v. 1.1 at registration and at completion of every even cycle until treatment discontinuation. Patients were treated until disease progression, intolerability, or patient request to discontinue. Aliquots from research blood samples were assayed immediately by flow cytometry, while additional aliquots were processed to preserve viable peripheral blood mononuclear cells (PBMCs) and plasma for batched analysis.

### Study oversight

This study protocol was approved by the Mayo Clinic IRB in accordance with assurances filed with and approved by the Department of Health and Human Services. The study was conducted in accordance with the Declaration of Helsinki and International Conference on Harmonization Guidelines for Good Clinical Practice. The study was registered at Clinicaltrials.gov as NCT03021460. All patients provided written informed consent. The study was designed by the senior academic authors. The study medication, ibrutinib, was provided by the sponsor, Pharmacyclics. The protocol is available online (**Supplementary Materials**).

### Statistical analysis

All patients meeting the eligibility criteria who provided written informed consent and began protocol-directed therapy were included in the description of baseline characteristics and analysis of safety and clinical outcomes. The data lock for this report was June 11, 2024. The primary aim was to determine the maximum tolerated dose (MTD) of ibrutinib in combination with pembrolizumab. MTD was defined as the highest dose level in which at most 1 of 6 patients developed a DLT during the first treatment cycle. Secondary endpoints included the safety profile of pembrolizumab and ibrutinib and the tumor response rate defined as the proportion of patients whose disease meets the RECIST v. 1.1 for partial or complete response (PR or CR) on two consecutive evaluations at least 8 weeks apart. Clinical benefit is defined as remaining on protocol treatment (regardless of dose reductions or discontinuing one of the agents) and progression-free for at least 6 months. The maximum grade of each type of toxicity was recorded for each patient and the number of patients developing any grade of that toxicity as well as the number developing a severe degree of that toxicity will be tabled.

Duration of tumor response was defined as the time from registration to disease progression or treatment discontinuation for any reason. Progression-free survival (PFS) was defined as the time from registration to documentation of disease progression. Overall survival (OS) was defined as the time from registration to death due to any cause. The distribution of event times was estimated using the Kaplan-Meier method.

Given the small sample size and the likelihood of few tumor responses in this patient population, the correlative aims were undertaken to gather preliminary data for generating hypotheses to be tested in future studies with an appropriate sample size to detect clinically meaningful differences. Exact Wilcoxon rank sum tests were used to assess for differences between patients deriving clinical benefit (6+ months on protocol treatment without progression—CB) and those without CB.

### Immunophenotyping

Fresh peripheral blood collected in EDTA tubes was labeled for same-day flow cytometry with antibodies to B7-H1, CD3, CD4, CD8, CD11b, CD14, CD15, CD16, CD19, CD21, CD25, CD27, CD28, CD33, CD40, CD44, CD45RA, CD45RO, CD56, CD62L, CD66b, CD86, CD123, CD142, CD154, CD197, CD203c, CTLA-4, HLA-DR, IgD, IgM, PD-1, and TCR gamma delta. The details of the antibodies used can be found in **Supplementary Table 1**. All flow cytometry procedures, antibodies, flow protocols and gating strategies were previously described in our work (19–21).

### Th1/Th2 polarity assessment

T cells were isolated from frozen PBMCs (1 x 10^7^ PBMCs/100 mcl) with CD3 paramagnetic beads (Thermo Fisher Scientific, Waltham, MA, catalog number 8802-6830-74) per the manufacturer’s instructions. The purified T cells (8 x 10^4^ cells per well in a 96-well plate) were incubated with anti-CD3 and anti-CD28 antibody-containing beads (Thermo Fisher Scientific, Waltham, MA, catalog number 11161D) at a 1:1 ratio for 24 hours at 37°C. Following stimulation, cell culture supernatants were assessed for interferon gamma (IFNγ) and interleukin 4 (IL-4) by ELISA (R & D Systems, Minneapolis, MN, catalog numbers DIF50C and M4000B-1) according to the manufacturer’s instructions. Absorbance at 450 nm was read on a Spectra Max Plus microplate reader (Molecular Devices, San Jose, CA).

## Results

### Baseline characteristics

From January 31, 2017 to January 9, 2023, a total of 17 individuals were registered onto the study. Prior to the initiation of protocol therapy one patient died. The patient was an 81-year-old male assigned to DL0 who presented at the emergency room with acute abdominal pain following his pre-registration research liver biopsy. The patient was placed in intensive care for hemorrhagic shock but failed to recover and died.

**Figure 1:**
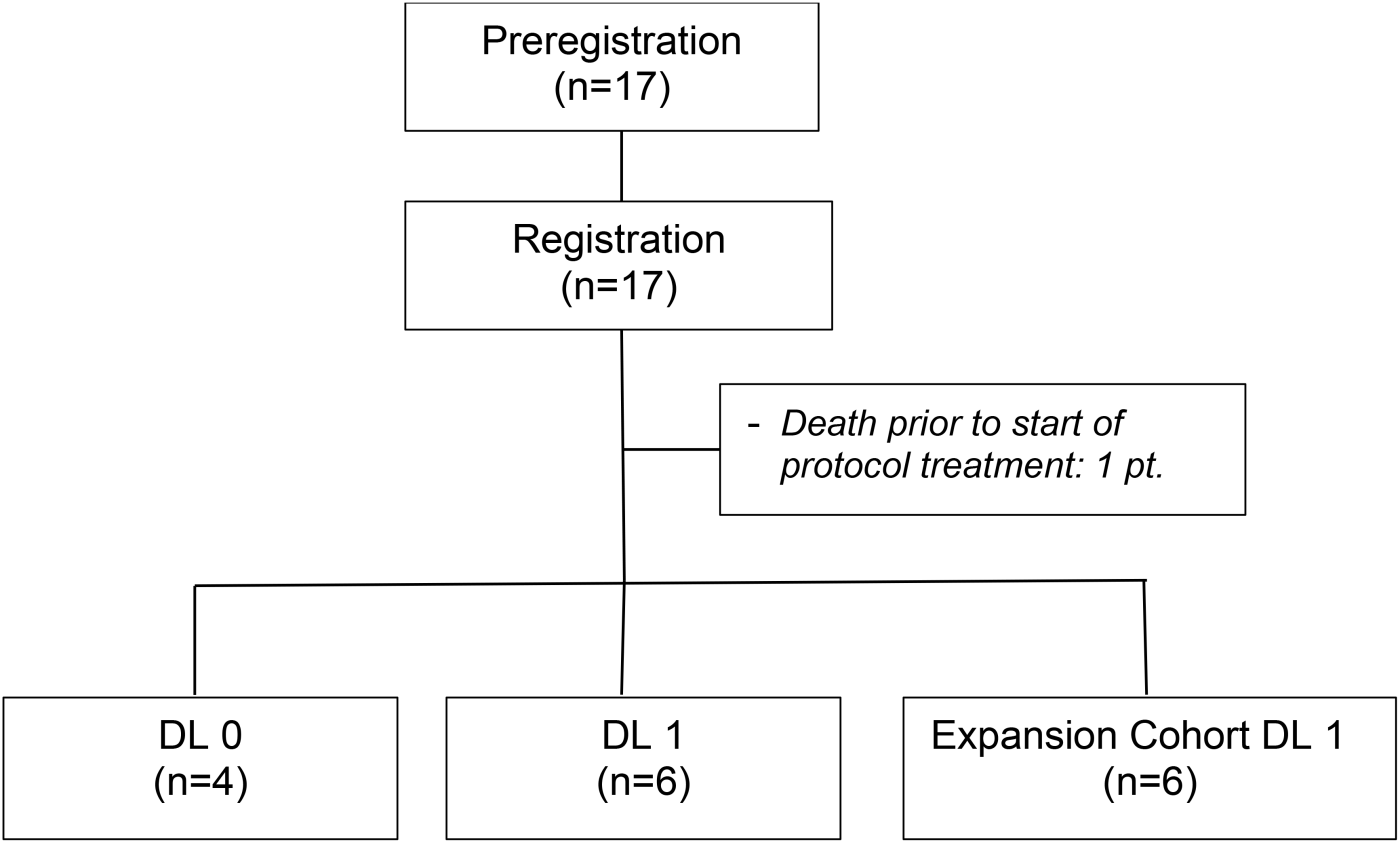
Consort diagram.

The remaining 16 patients (10 males; 6 females) comprise the analysis cohort (**Figure 1**). The patient and tumor baseline characteristics of these 16 patients are presented in **Table 1**. Fifty percent of patients were between 70 to 79 years of age. Six patients (37.5%) had a BRAF V600E or V600K mutation. The most common sites of metastases were lymph nodes (50%), lung (47.8%), and liver (37.5%). Four patients (25%) had prior systemic therapy, namely ipilimumab and nivolumab, and had progressed within 6 months after the last treatment dose.

**Table 1:** Patient characteristics at registration.

Characteristic	no. (%)
	(N=16)
Age (years)	
35 - 49	1 (6.3%)
50 - 59	3 (18.8%)
60 - 69	4 (25.0%)
70 - 79	8 (50.0%)
Gender	
Male	10 (62.5%)
Female	6 (37.5%)
Race/Ethnicity	
White/non-Hispanic and non-Latina	17 (100%)
ECOG PS	
0	15 (93.8%)
2	1 (6.3%)
BRAF V600E or V600K mutation	
Yes	6 (37.5%)
No	9 (56.3%)
Insufficient tissue/DNA to ascertain	1 (6.3%)
Sites of Metastases	
Lymph nodes	8 (50.0%)
Lung	7 (47.8%)
Liver	6 (37.5%)
Subcutaneous tissue	3 (18.8%)
Soft tissue/skin	2 (12.5%)
Bone	1 (6.3%)
Prior therapy	
None	12 (75%)
Adjuvant nivolumab+/-ipilimumab	4 (25%)

### Treatment course, tumor response, and adverse events

Four patients were enrolled onto DL0 before enrollment was temporarily halted. None of these 4 patients developed a DLT. Enrollment to DL1 was then opened, and none of the first cohort of 3 patients enrolled developed a DLT. As no higher dose levels were planned, dose escalation ceased and a second cohort of 3 patients was enrolled to DL1 to confirm it as the MTD. None of these 3 additional patients developed a DLT, and DL1 was established as the MTD. Enrollment was then re-opened at DL1 for an expansion cohort of 6 patients.

All patients have discontinued all protocol treatment. The median number of treatment cycles administered was 3 (range: 1-27). One patient on DL1 required an ibrutinib dose reduction after cycle 1 due to grade 3 arthralgia. None of the patients discontinued one agent and continued the other. However, there were 3 patients who discontinued all protocol therapy due to either a Grade 4 alanine aminotransferase (ALT) increase and a Grade 4 aspartate aminotransferase (AST) increase after 3 cycles of treatment on DL0 (1 pt), a Grade 4 ALT increase and Grade 3 AST increase after 2 cycles of treatment on DL1 (1 pt), or a Grade 3 hyponatremia, hypoxia, and maculopapular rash (which resolved with topical steroids) after 1 cycle of treatment on DL1 (1 patient). **Table 2** provides the toxicities reported across all cycles regardless of attribution.

**Table 2:** Grade 2-5 toxicities reported regardless of attribution.

Event	Grade 1 n (%)	Grade 2 n (%)	Grade 3 n (%)	Grade 4 n (%)	Grade 5 n (%)
Abdominal pain	0	0	1 (6.3)	0	0
Alanine aminotransferase increased	0	1 (6.3)	0	2 (12.5)	0
Alopecia	0	1 (6.3)	0	0	0
Anemia	5 (31.3)	1 (6.3)	0	0	0
Arthralgia	0	1 (6.3)	1 (6.3)	0	0
Aspartate aminotransferase increased	0	0	1 (6.3)	1 (6.3)	0
Back pain	0	0	1 (6.3)	0	0
Blood bilirubin increased	2 (12.5)	2 (12.5)	0	0	0
Diarrhea	3 (18.8)	2 (12.5)	0	0	0
Dry mouth	0	1 (6.3)	0	0	0
Dyspnea	4 (25.0)	0	0	0	0
Fatigue	9 (56.3)	2 (12.5)	0	0	0
Fever	2 (12.5)		0	0	0
General disorders/administrative site conditions	0	1 (6.3)	0	0	0
Hypertension	0	0	1 (6.3)	0	0
Hyponatremia	0	0	1 (6.3)	0	0
Hypoxia	0	0	1 (6.3)	0	0
Lymphocyte count decreased	0	1 (6.3)	1 (6.3)	0	0
Mucosal infection	0	1 (6.3)	0	0	0
Nausea	0	1 (6.3)	0	0	0
Pain	0	1 (6.3)	1 (6.3)	0	0
Pain in extremity	0	1 (6.3)	0	0	0
Purpura	0	1 (6.3)	0	0	0
Rash maculo-papular	4 (25.0)	0	1 (6.3)	0	0
Upper respiratory infection	0	1 (6.3)	0	0	0

Of the other 13 patients, protocol treatment was discontinued due to disease progression (10 pts) or physician/patient decision after 22-27 cycles of treatment having had a partial or complete tumor response (3 pts). These 3 patients were the only ones with a tumor response. Specifically, 1 patient on DL0 had a complete tumor response lasting 12 months before deciding to discontinue treatment and 2 patients on DL1 had a partial response lasting 8.3 and 12.8 months before deciding to discontinue. Thus, the tumor response rate was 18.5% (95% CI: 4.1-45.7%).

### Progression-free and overall survival

**Figure 2:**
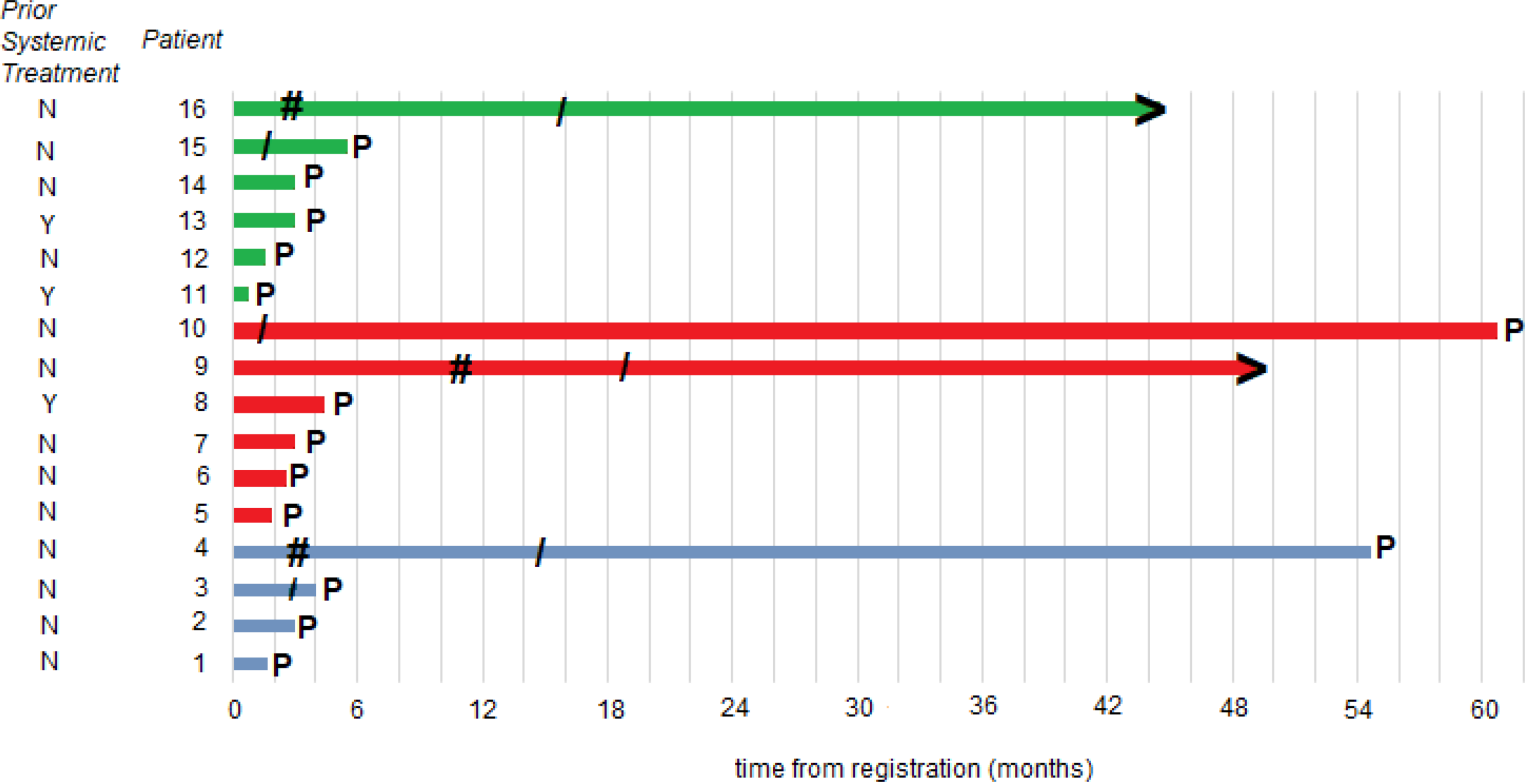
Patient responses. Swimmer plot illustrates the time to disease progression. The time point at which progression occurred is denoted as P; the starting point of an objective response is denoted as #; the end of protocol treatment is denoted as/; patients without progression at the time of data cutoff are denoted with >; a blue line is a DL0 patient; a red line is a DL1 dose escalation cohort patient; and a green line is a DL1 dose expansion cohort patient.

At the time of the data lock, 3 patients were alive without disease progression, 4 were alive having had disease progression, and 9 were dead following disease progression. The median time to disease progression was 3 months with a 6-month PFS rate of 25.0% (95%CI: 10.7 - 56.4%). The median OS was 1.8 years. Clinical events (tumor response, disease progression, death) and duration of response, treatment, and freedom from progression are summarized in the swimmer plot (**Figure 2**).

### Translational studies

**Figure 3:**
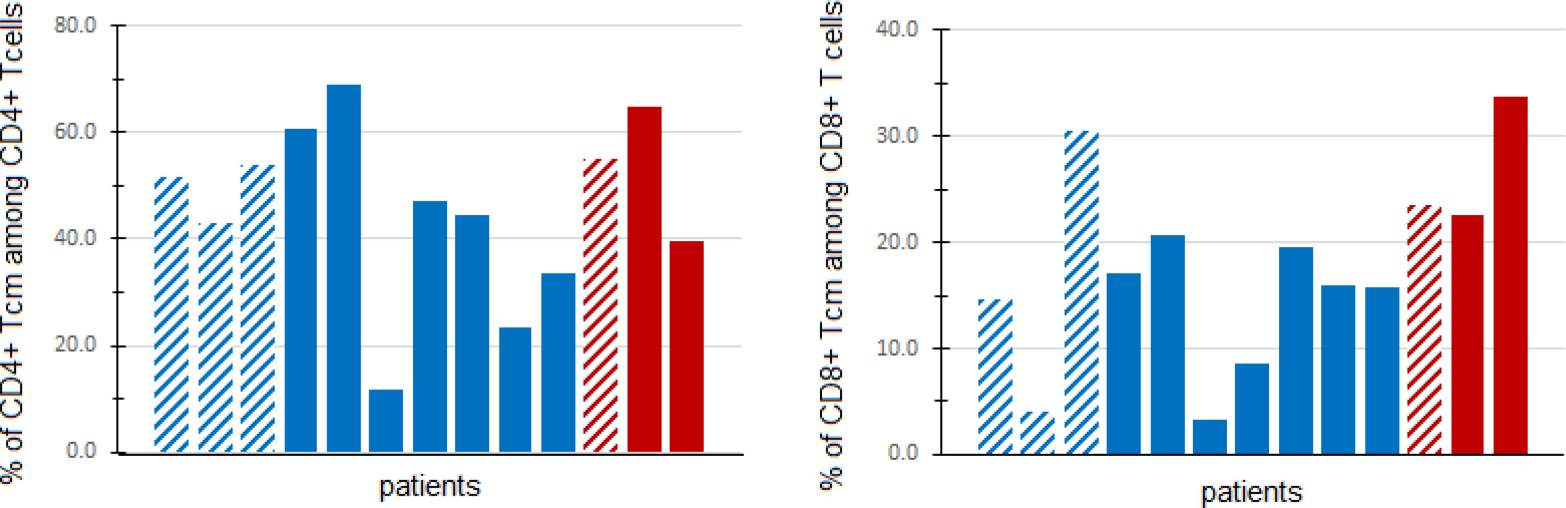
Central memory T cells in peripheral blood. Fresh PBMCs from baseline blood samples were stained with multiple antibodies and assessed for central memory T cells (T_CM_) (CD45RO+CD197+) via flow cytometry. The percentage of CD4+ (left panel) and CD8+ (right panel) T_CM_ (out of all CD4+ and CD8+ T cells, respectively) are shown for patients with clinical benefit (CB, defined as progression-free and on treatment at least 6 months) (red) and patients without CB (blue). Patients in DL0 are denoted with dashed columns, while DL1 patients are denoted with solid columns.

We explored the relationship between peripheral blood immune cell populations with 6-month clinical benefit (CB), defined as remaining on protocol treatment for at least 6 months (6 cycles of treatment) without disease progression. Blood specimens for immunophenotyping were provided by 13 patients: 3 patients with CB, and 10 patients without CB. There was a higher percentage (Wilcoxon rank sum test p=0.028) of CD8+ central memory T cells (T_CM_) among patients with CB (median 23.5%, range 22.7-33.7%) than among patients without CB (median: 15.8%, range 3.3-30.6%). However, CD4+ T_CM_ were not found to differ significantly (Wilcoxon rank sum test p=0.398) between patients with CB (median 55.0%, range 39.6-65.0%) and those without CB (median 45.8%, range 11.6-68.9%), as shown in **Figure 3**.

The percentages of CD8+ T cells, CD4+ T cells, Tregs, B cells, memory B cells, myeloid-derived suppressor cells (MDSCs), and CD33+ monocytes, as well as the ratios of neutrophil to lymphocyte counts and monocyte to lymphocyte counts were similar among those with versus without CB (all exact Wilcoxon rank sum test p-values > 0.05, **Supplementary Figure 1**).

**Figure 4:**
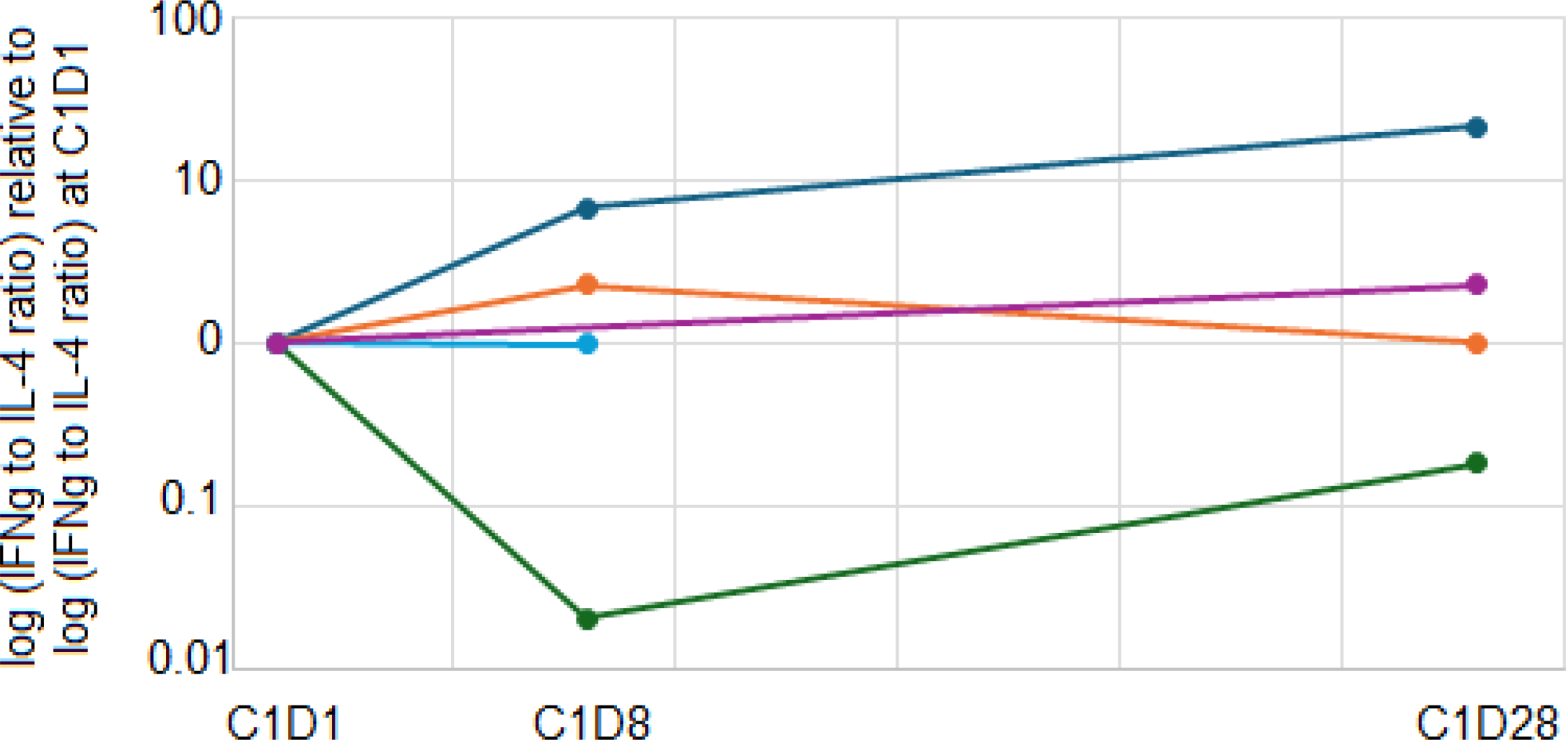
Immune polarity changes. Patient PBMCs from baseline, C1D8, and C1D28 were stimulated with anti-CD3 and anti-CD28. After 24 hours, culture supernatants were harvested, and IFNγ and IL-4 concentrations were quantitated. The ratio of IFNγ:IL-4 was calculated and normalized to the baseline ratio. Navy, orange, blue, purple and green lines represent five different patients.

We also explored whether the addition of ibrutinib to pembrolizumab increased the ratio of Th1 to Th2 immune responses by measuring the Th1 cytokine IFNγ and the Th2 cytokine IL-4 produced by stimulated patient PBMCs before and after ibrutinib therapy. After stimulation with anti-CD3 and anti-CD28 antibodies *in vitro* for 24 hours, 5 patients (1 DL0, 4 DL1) had detectable levels of both IFNγ and IL-4 at baseline and at least one post-treatment timepoint. One patient on DL0 had a clear increase in the IFNγ:IL-4 ratio after 1 week of ibrutinib therapy that persisted until the completion of the first cycle of pembrolizumab. That patient discontinued protocol treatment after 2 months due to toxicity. The other 4 patients did not have a sustained increase in the Th1:Th2 ratio and discontinued protocol therapy after 3-4 months due to toxicity (**Figure 4**). The IFNγ and IL-4 levels are shown in **Supplementary Figure 2**.

## Discussion

As melanoma patients frequently exhibit evidence of Th2 polarization of the immune response which could compromise the ability of the immune system to eradicate melanoma cells, the addition of ibrutinib could potentially drive a change in Th1/Th2 immune polarity in favoring Th1 anti-tumor immune responses, thus leading to an improvement in the objective response rate of advanced melanoma patients over that expected by pembrolizumab alone. Based on previously published safety data (11), this trial was originally designed to assess the anti-tumor activity of ibrutinib 560 mg daily with pembrolizumab 200 mg every 3 weeks. However, each of the first two patients developed grade 3 skin rash and withdrew for the study. The study was then redesigned as a 3 + 3 phase 1 clinical trial to evaluate the safety, tolerability, and determine the MTD of ibrutinib in combination with pembrolizumab in patients with metastatic melanoma, which was determined to be 420 mg daily.

Our study is the first study to investigate the combination of ibrutinib and pembrolizumab in melanoma. Prior phase 2 study by Moschos et al. did not show any meaningful clinical benefit using ibrutinib alone in systemic treatment-refractory distant metastatic cutaneous melanoma (22). The combination of an ITK inhibitor and pembrolizumab was probed in other solid tumors, including mismatch repair proficient metastatic colorectal cancer (23), metastatic urothelial cancer (24), and advanced pancreatic cancer (25). In the phase 1 colorectal cancer study, the combination of ibrutinib and pembrolizumab was tested, and in contrast to our study, ibrutinib was well-tolerated when dosed at 560 mg once daily, and an MTD was not identified. The difference in MTD could be due to the difference in immunogenicity between the two different tumor types. In all three studies (2 combining acalabrutinib and 1 combining ibrutinib with pembrolizumab) (23–25), no significant anti-cancer activity was found.

While our study did not have sufficient patients for efficacy comparison with pembrolizumab monotherapy, concurrent treatment with ibrutinib and pembrolizumab did not lead to substantially more clinical benefit than would be expected from pembrolizumab alone. Flow cytometry data of patients treated with this combination showed a higher proportion of CD8+ T_CM_ in non-progressors, although a limited number of patients precludes formal comparison. Interestingly, we also observed higher frequencies of activated CD8+ T cells in a portion of patients with no prolonged CB (**Supplementary Figure 1F**). Although activated CD8+ T cells are thought to be associated with favorable outcomes, several other possible explanations of the data exist. First, the number of patients assessed is quite small and precludes formal statistical comparison of the groups. Second, it is possible that some of the patients in the No CB group were responding to treatment initially (the assay was conducted on baseline samples) but later experienced a change in immune potential leading to progression. Finally, activated CD8+ T cells as identified by flow cytometry could include T cells that are reactive to viruses or other non-melanoma antigens, so it is possible that this does not predict a favorable response.

After ibrutinib treatment, we did not observe a consistent increase in the ratio of Th1:Th2 immune responses. It is not clear whether the lack of consistent improvement in the Th1:Th2 ratio is due to an inadequate exposure of lymphocytes to ibrutinib, or whether the immune milieu of metastatic melanoma patients drives Th2 differentiation in a manner that does not require ITK. Further study with a larger number of patients would be needed for further clarification.

In summary, among the dose levels of ibrutinib tested in combination with pembrolizumab, the safety profile was acceptable and durable clinical benefit was observed in a subset of patients. The maximum tolerated dose of ibrutinib in combination with pembrolizumab was identified as 420 mg daily. Observations in the correlative studies are hypothesis-generating and would need further exploration in a larger study.

## Data Availability

The original contributions presented in the study are included in the article/**Supplementary Material**. Further inquiries can be directed to the corresponding author/s.

## Supplementary material

Supplementary Figure 1 Immune cell subpopulations in peripheral blood. Fresh PBMCs from baseline blood samples were stained with multiple antibodies and assessed for immune cell subpopulations via flow cytometry. The neutrophil count per mL of blood **(A)**; percentage of IgD+ B cells among all B cells **(B)**; percentage of memory B cells among all B cells **(C)**; percentage of myeloid-derived suppressor cells (MDSCs) among all nucleated cells **(D)**; percentage of activated CD4+ T cells among all CD4+ T cells **(E)**; percentage of activated CD8+ T cells among all CD8+ T cells **(F)**; tumor-related CD4+ T cells among all CD4+ T cells **(G)** and tumor-related CD8+ T cells among all CD8+ T cells **(H)**; CD14+ monocytes among all monocytes **(I)**; CD33+ monocytes among all monocytes **(J)**, percentage of Treg among CD4+ T cells **(K)**, ratio of monocyte count to lymphocyte count **(L)**; and neutrophils to lymphocytes ratio **(M)**, are shown for patients with clinical benefit (CB, defined as progression-free and on treatment at least 6 months) (red) and patients without CB (blue). Patients in DL0 are denoted with dashed columns, while DL1 patients are denoted with solid columns.

Supplementary Figure 2 Immune polarity changes. Patient PBMCs from baseline, C1D8, and C1D28 were stimulated with anti-CD3 and anti-CD28. After 24 hours, culture supernatants were harvested, and IFNγ **(A)** and IL-4 **(B)** concentrations were quantitated and shown in the figures. Navy, orange, blue, purple and green lines represent five different patients.

Supplementary Table 1 Antibodies and reagents used for flow cytometry.
