# Supplementary material for: Phase I study of pembrolizumab in combination with ibrutinib for the treatment of unresectable or metastatic melanoma
Source: Front Immunol. 2025 Feb 4;16:1491448. doi: 10.3389/fimmu.2025.1491448 (PMC11832643; doi:10.3389/fimmu.2025.1491448)
Supplement: Supplementary Figure 1 — Immune cell subpopulations in peripheral blood. Fresh PBMCs from baseline blood samples were stained with multiple antibodies and assessed for immune cell subpopulations via flow cytometry. The neutrophil count per mL of blood (A); percentage of IgD+ B cells among all B cells (B); percentage of memory B cells among all B cells (C); percentage of myeloid-derived suppressor cells (MDSCs) among all nucleated cells (D); percentage of activated CD4+ T cells among all CD4+ T cells (E); percentage of activated CD8+ T cells among all CD8+ T cells (F); tumor-related CD4+ T cells among all CD4+ T cells (G) and tumor-related CD8+ T cells among all CD8+ T cells (H); CD14+ monocytes among all monocytes (I); CD33+ monocytes among all monocytes (J), percentage of Treg among CD4+ T cells (K), ratio of monocyte count to lymphocyte count (L); and neutrophils to lymphocytes ratio (M), are shown for patients with clinical benefit (CB, defined as progression-free and on treatment at least 6 months) (red) and patients without CB (blue). Patients in DL0 are denoted with dashed columns, while DL1 patients are denoted with solid columns. [file Image1.pdf]

Supplementary Figure 1. Immune cell subpopulations in peripheral blood. Fresh PBMCs from baseline blood samples were stained with multiple antibodies and assessed for immune cell subpopulations via flow cytometry. The neutrophil count per mL of blood (Panel A); percentage of IgD+ B cells among all B cells (Panel B); percentage of memory B cells among all B cells (Panel C); percentage of myeloid-derived suppressor cells (MDSCs) among all nucleated cells (Panel D); percentage of activated CD4+ T cells among all CD4+ T cells (Panel E); percentage of activated CD8+ T cells among all CD8+ T cells (Panel F); tumor-related CD4+ T cells among all CD4+ T cells (Panel G) and tumor-related CD8+ T cells among all CD8+ T cells (Panel H); CD14+ monocytes among all monocytes (Panel I); CD33+ monocytes among all monocytes (Panel J) , percentage of Treg among CD4+ T cells (Panel K), ratio of monocyte count to lymphocyte count (Panel L); and neutrophils to lymphocytes ratio (Panel M), are shown for patients with clinical benefit (CB, defined as progression-free and on treatment at least 6 months) (red) and patients without CB (blue). Patients in DL0 are denoted with dashed columns, while DL1 patients are denoted with solid columns.

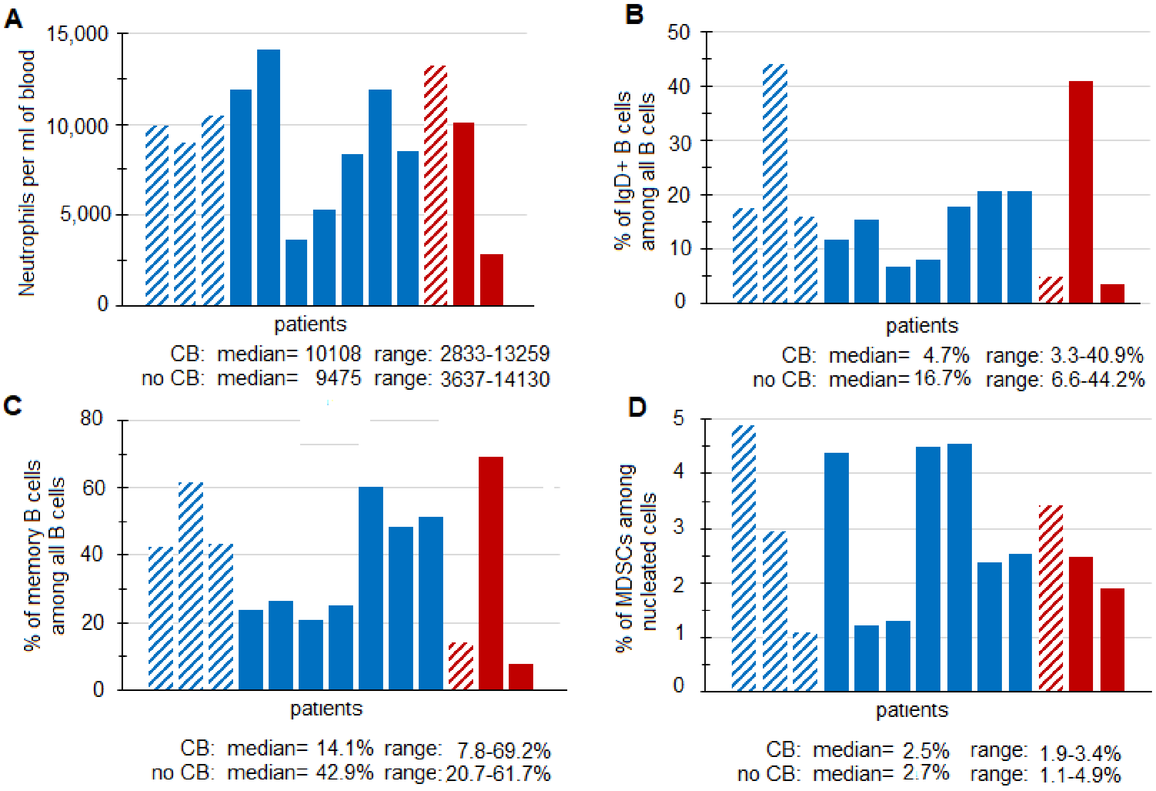

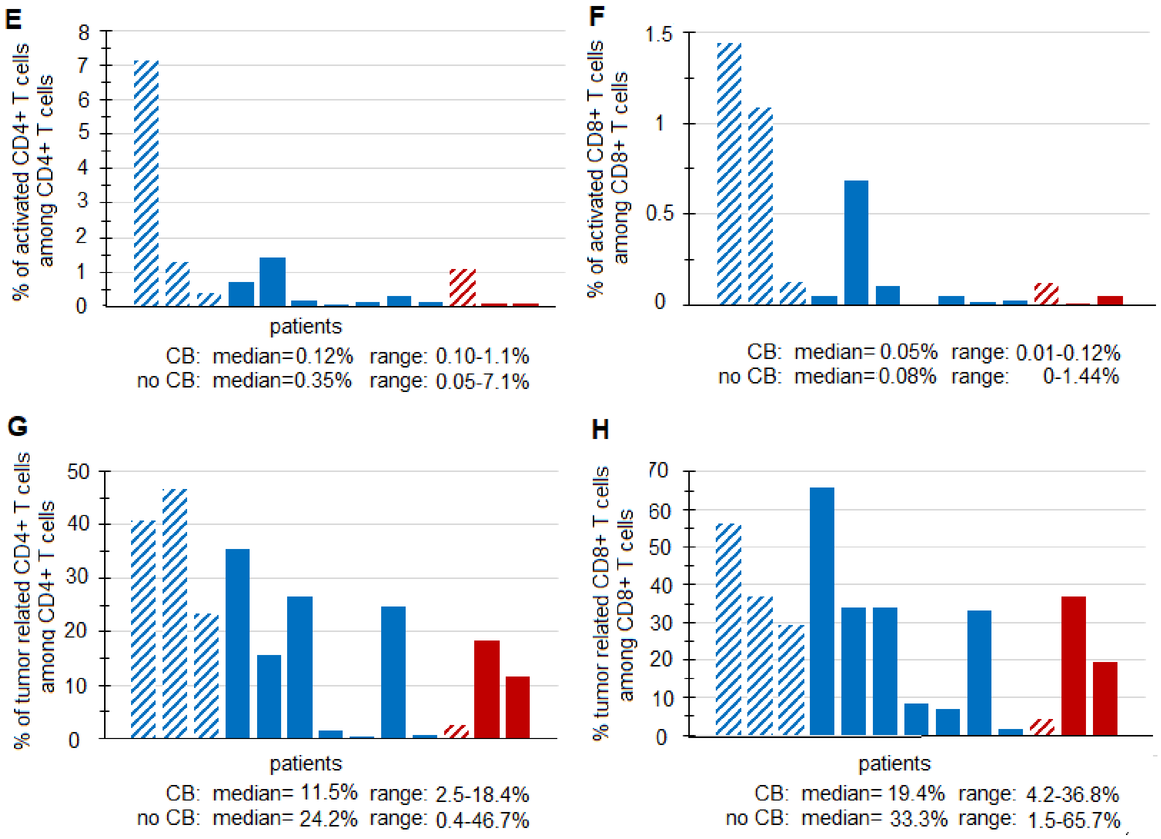
E
% of activated CD4+ T cells among CD4+ T cells
patients
CB: median=0.12% range: 0.10-1.1%
no CB: median=0.35% range: 0.05-7.1%
F
% of activated CD8+ T cells among CD8+ T cells
CB: median=0.05% range: 0.01-0.12%
no CB: median=0.08% range: 0-1.44%
G
% of tumor related CD4+ T cells among CD4+ T cells
patients
CB: median= 11.5% range: 2.5-18.4%
no CB: median= 24.2% range: 0.4-46.7%
H
% of tumor related CD8+ T cells among CD8+ T cells
patients
CB: median= 19.4% range: 4.2-36.8%
no CB: median= 33.3% range: 1.5-65.7%

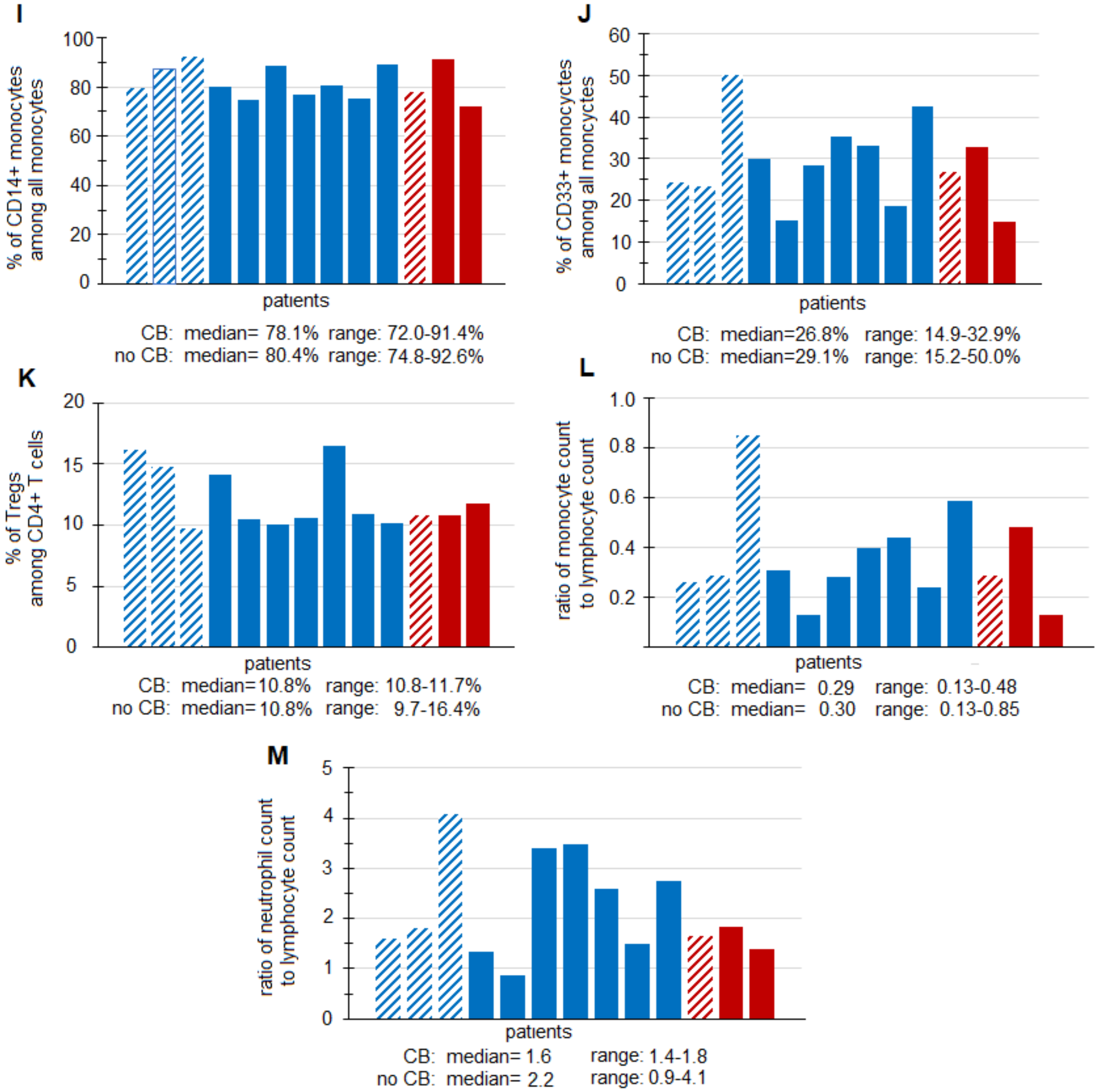
I
% of CD14+ monocytes among all monocytes
patients
CB: median= 78.1% range: 72.0-91.4%
no CB: median= 80.4% range: 74.8-92.6%
J
% of CD33+ monocytes among all moncytes
patients
CB: median=26.8% range: 14.9-32.9%
no CB: median=29.1% range: 15.2-50.0%
K
% of Tregs among CD4+ T cells
patients
CB: median=10.8% range: 10.8-11.7%
no CB: median=10.8% range: 9.7-16.4%
L
ratio of monocyte count to lymphocyte count
patients
CB: median= 0.29 range: 0.13-0.48
no CB: median= 0.30 range: 0.13-0.85
M
ratio of neutrophil count to lymphocyte count
patients
CB: median= 1.6 range: 1.4-1.8
no CB: median= 2.2 range: 0.9-4.1

Supplemental Figure 2. Immune polarity changes. Patient PBMCs from baseline, C1D8, and C1D28 were stimulated with anti-CD3 and anti-CD28. After 24 hours, culture supernatants were harvested, and IFNγ (A) and IL-4 (B) concentrations were quantitated and shown in the figures. Navy, orange, blue, purple and green lines represent five different patients.

A

B

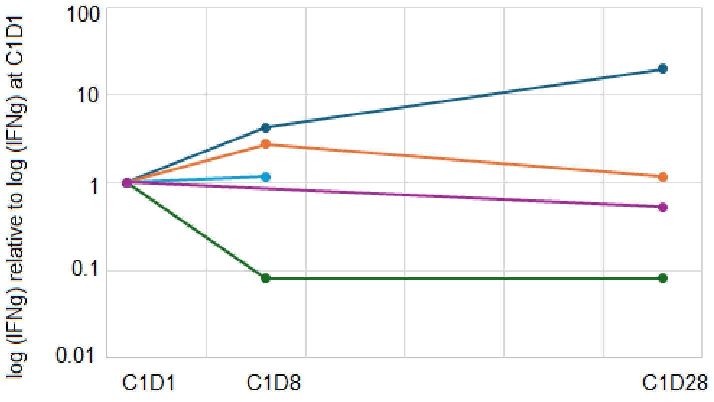

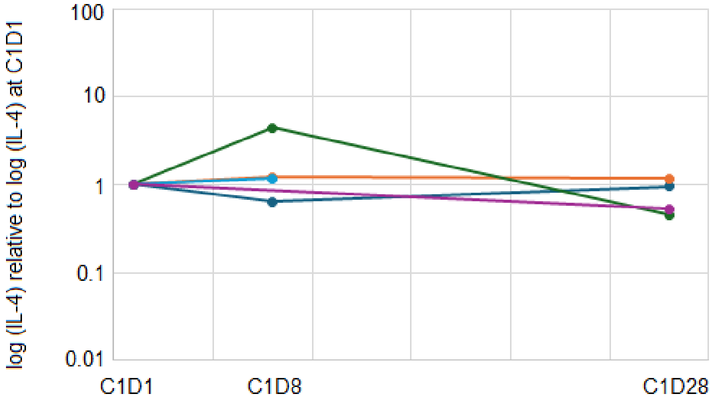

Supplementary Table 1. Antibodies and reagents used for flow cytometry.

Antibody	Company	Catalog number	Clone number
CD3 APC-750	Coulter	A66329	UCHT1
CD3 KrO	Coulter	B00068	UCHT1
CD3 PC7	Coulter	6607100	UCHT1
CD4 Pac Blu	Coulter	A82789	13B8.2
CD8 FITC	Coulter	IM0451U	B9.11
CD8 APC-700	Coulter	A66332	B9.11
CD8 KrO	Coulter	B00067	B9.11
CD11b PC7	Coulter	A54822	Bear1
CD14 ECD	Coulter	IM2707U	RMO52
CD14 PC5.5	Coulter	A70204	RMO52
CD15 FITC	Coulter	IM1423U	80H5
CD15 Pac Blu	Coulter	A74775	80H5
CD16 APC-750	Coulter	A66330	3G8
CD16 APC-700	Coulter	B20023	3G8
CD16 ECD	Coulter	A33098	3G8
CD19 APC	Coulter	IM2470U	J3-119
CD19 APC-700	Coulter	A78837	J3-119
CD21 Pac Blu	Coulter	B09982	BL13
CD25 PC5.5	Coulter	A79386	B1.49.9
CD27 PE	Coulter	IM2578	1A4CD27
CD28 APC-750	Coulter	B08757	CD28.2
CD33 APC	Coulter	IM2471U	D3HL60.251
CD40 APC	BD	555591	5C3
CD44 PerCp Cy 5.5	BD	560531	G44-26
CD45RA APC-750	Coulter	A86050	2H4LDH11LDB9
CD45RO ECD	Coulter	IM2712U	UCHL1
CD56 PC7	Coulter	A51078	N901 (HLDA6)
CD62L APC	BD	559722	DREG-56
CD66b FITC	Coulter	IM0531U	80H3
CD66b APC-750	Coulter	B08756	80H3
CD86 APC	BD	555660	2331 (FUN-1)
CD123 PE	Coulter	A32535	SSDCLY107D2
Gamma delta TCR PE	Coulter	IM1418U	IMMU510
HLA-DR ECD	Coulter	IM3636	Immu-357
HLA-DR Pac Blu	Coulter	A74781	Immu-357
IgD FITC	Coulter	B30652	IA6-2
IgM APC	Coulter	735972	SA-DA4
