# Supplementary material for: Phase I study of pembrolizumab in combination with ibrutinib for the treatment of unresectable or metastatic melanoma
Source: Front Immunol. 2025 Feb 4;16:1491448. doi: 10.3389/fimmu.2025.1491448 (PMC11832643; doi:10.3389/fimmu.2025.1491448)
Supplement: Supplementary Table 1 — Antibodies and reagents used for flow cytometry. [file DataSheet1.docx]

Mayo Clinic Cancer Center

# MC1577 Phase I Study of Pembrolizumab in Combination with Ibrutinib in the Treatment of Unresectable or Metastatic Melanoma

Study Chairs: Matthew S Block MD PhD*

Mayo Clinic

200 First Street SW Rochester, MN 55905 507/284-2511

Study Cochairs: Yiyi Yan, MD PhD

Svetomir N Markovic MD PhD Statistician: Vera J Suman PhD√

*Investigator having NCI responsibility for this protocol

√Study contributor(s) not responsible for patient care

**Drug Availability**

**Supplied Investigational Agents:** Ibrutinib **Commercial Agents:** Pembrolizumab **Drug Company Supplied:** Ibrutinib

| **Document History** | **(Effective Date)** |
| --- | --- |
| Activation | January 31, 2017 |
| MCCC Addendum 1 | May 24, 2017 |
| MCCC Amendment 2 | November 21, 2017 |
| MCCC Amendment 3 | September 27, 2018 |
| MCCC Amendment 4 | August 5, 2019 |
| MCCC Amendment 5 | February 6, 2020 |
| MCCC Amendment 6 | October 23, 2020 |
| MCCC Amendment 7 | June 3, 2021 |
| MCCC Amendment 8 | November 3, 2022 |

# Protocol Resources

| **Questions:** | **Contact Name:** |
| --- | --- |
| Patient eligibility*****, test schedule, treatment delays/interruptions/adjustments, dose modifications, adverse events,  forms completion and submission | Katie A Hervey Data Manager  Phone: (507) 266-6117  E-mail: [hervey.katie@mayo.edu](mailto:hervey.katie@mayo.edu) |
| Drug administration, infusion pumps, nursing guidelines | Lisa A Kottschade, APRN, CNP Phone: (507) 293-0571  E-mail: [kottschade.lisa@mayo.edu](mailto:kottschade.lisa@mayo.edu) |
| Forms completion and submission | Robbin A Peterson CCRP Phone: (507) 266-3234  Email: [peterson.robbin@mayo.edu](mailto:peterson.robbin@mayo.edu) |
| Protocol document, consent form, regulatory issues | Lynn Flickinger Phone: (507) 284-0938  Email: [flickinger.lynn@mayo.edu](mailto:flickinger.lynn@mayo.edu) |
| Non-paraffin biospecimens | Courtney L Erskine Biospecimen Resource Manager Phone: (507)284-4488  Email: [erskine.courtney@mayo.edu](mailto:erskine.courtney@mayo.edu) |
| Serious Adverse Events | SAE Coordinator  E-mail: [INDSafety@mayo.edu](mailto:INDSafety@mayo.edu) |

*No waivers of eligibility per NCI

# Table of Contents

[MC1577 Phase I Study of Pembrolizumab in Combination with Ibrutinib in the Treatment of](#_bookmark0) [Unresectable or Metastatic Melanoma 1](#_bookmark0)

[Protocol Resources 2](#_bookmark1)

[Table of Contents 3](#_bookmark2)

[Schema 4](#_bookmark3)

1. [Background 5](#_bookmark4)
2. [Goals 8](#_bookmark5)
3. [Patient Eligibility 9](#_bookmark6)
4. [Test Schedule 12](#_bookmark7)
5. [Grouping Factors 13](#_bookmark8)
6. [Registration Procedures 13](#_bookmark9)
7. [Protocol Treatment 15](#_bookmark10)
8. [Dosage Modification Based on Adverse Events 17](#_bookmark11)
9. [Ancillary Treatment/Supportive Care 25](#_bookmark12)
10. [Adverse Event (AE) Monitoring and Reporting 28](#_bookmark13)
11. [Treatment Evaluation Using RECIST Guideline 42](#_bookmark14)
12. [Descriptive Factors 47](#_bookmark15)
13. [Treatment/Follow–up Decision at Evaluation of Patient 47](#_bookmark16)
14. [Body Fluid Biospecimens 50](#_bookmark17)
15. [Drug Information 54](#_bookmark18)
16. [Statistical Considerations and Methodology 61](#_bookmark19)
17. [Pathology Considerations/Tissue Biospecimens 66](#_bookmark20)
18. [Records and Data Collection Procedures 68](#_bookmark21)
19. [Budget 68](#_bookmark22)
20. [References 69](#_bookmark23)

[Appendix I - MC1577 Patient Medication Diary 71](#_bookmark48)

[Appendix II - ECOG Performance Status 73](#_bookmark49)

[Appendix III - CTCAE 74](#_bookmark50)

[Appendix IV - Potential Drug-Drug Interactions for Ibrutinib 75](#_bookmark51)

[Appendix V - Inhibitors and Inducers of CYP3A 77](#_bookmark52)

[Appendix VI - Expected Adverse Reactions of Ibrutinib 78](#_bookmark53)

[Appendix VII - Child-Pugh Score 82](#_bookmark54)

[Appendix VIII – Ibrutinib Additional Safety Information from Pharmacyclics 83](#_bookmark55)

# Schema

| **Pre-registration** | |  | | |
| --- | --- | --- | --- | --- |
|  |  | | | |
|  |  | Failure to confirm histologically or cytologically unresectable stage III or metastatic melanoma |  | |
|  |  |  |  | **Off Study** |
|  |  |  |  |  |
|  |  |  |  | |
|  |  | | | |
| **Registration** | |  | | |
|  |  | | | |
| Pembrolizumab once every 3 weeks  Ibrutinib daily | |  | | |
|  |  | | | |
| Confirmed PD Unacceptable adverse events  or  Patient refusal to continue protocol treatment | |  | | |
|  |  | | | |
| **Event Monitoring** | |  | | |

If after problem case evaluation, the Data and Statistical Center find a patient to be ineligible or a cancel, please refer to Section 13.0 for follow-up information.

| Generic name: Ibrutinib Brand name(s): Imbruvica  Mayo Abbreviation: PCI-32765 Availability: Pharmacyclics LLC | Generic name: Pembrolizumab Brand name(s): Keytruda  Mayo Abbreviation: PEMBROLIZUMAB Availability: Commercial |
| --- | --- |

# Background

- 1. Immune checkpoint blockade in advanced melanoma

Melanoma is the fifth most common cancer in men and sixth in women in the United States. The survival rates decline steadily as the disease stage increases. In patients with metastatic melanoma, the 10-year overall survival is less than 10%, with an estimated 9,128 related deaths in the United States in 2011[.^1^](#_bookmark24) Chemotherapy, and later targeted therapy, had been the mainstay of treatment in the past, although surgery and radiation also play a role in management. Over the last decade, the understanding of the immune checkpoint pathway has paved the way for immunotherapy in metastatic melanoma. Over the last decade, the understanding of immune checkpoints and the effects of immune checkpoint inhibition has paved the way for recent success in cancer immunotherapy, including metastatic melanoma.

The importance of intact immune surveillance in controlling outgrowth of neoplastic transformation has been known for years. Immune checkpoints are crucial for maintenance of self-tolerance under normal physiological conditions[.^2-4^](#_bookmark25) However, this can be dysregulated in tumors as an important mechanism of tumor resistance.[^5^](#_bookmark26)[^,6^](#_bookmark27) Accumulating evidence shows that antitumor immune responses can be unleashed by immune checkpoint blockade, and blockade of cytotoxic T-lymphocyte antigen 4 (CTLA-4) and programmed death (PD-1) receptor have shown durable anti-melanoma effects.[^7^](#_bookmark28)[^,8^](#_bookmark29)

The PD-1 receptor-ligand interaction is a major pathway used by tumors to suppress immune control. PD-1 receptor (encoded by the gene Pdcd1) is an Ig superfamily member related to CD28 and CTLA-

4.[2](#_bookmark25) It is expressed on the cell surface of activated T-cells under normal conditions[.^2^](#_bookmark25) By binding to its ligand (PD-L1 and PD-L2), PD-1 down-regulates T-cell activation and therefore dampens unwarranted and excessive immune responses, including autoimmunity. Melanoma cells exploit the PD-1 checkpoint pathway to evade immune surveillance. The interaction between PD-L1 expressed on tumor and stromal cells and PD-1 on T cells can trigger inhibitory signaling pathways that reduce effector cell functions and T-cell-killing capacity. Blocking the PD-1/PD-L1 interaction has been shown to potentiate tumor-specific CD8+ T-cell infiltration and effector T-cell activation that promote tumor rejection[.^9^](#_bookmark30)[^,10^](#_bookmark31)

- 1. Pembrolizumab for advanced melanoma

Pembrolizumab is a human IgG4 PD-1 blocking antibody that is recently approved by the FDA for the treatment of patients with unresectable or metastatic melanoma and disease, including progression following ipilimumab and, if BRAF V600 mutation positive, a BRAF inhibitor. Pembrolizumab showed an unprecedented rate of durable clinical responses, with an overall response rate of 26% and median progression-free survival of 22 weeks (95% CI: 12-36 weeks)[.^8^](#_bookmark29) Treatment has generally been well-tolerated, and the most common drug-related adverse events (AEs) of any grade were fatigue (33%), pruritus (26%), and rash (18%). Patients responding to treatment showed proliferation of CD8 T cells in their tumors.[^10^](#_bookmark31)

Despite the promising data associated with pembrolizumab treatment, the majority of melanoma patients treated does not respond to pembrolizumab. Several possible mechanisms for pembrolizumab (and other PD-1 blockade) resistance exist, including additional mechanisms of immunosuppression in the tumor microenvironment and derangements in systemic immune competence[.^11^](#_bookmark32)[^,12^](#_bookmark33) Specifically, it has been demonstrated that patients with metastatic melanoma have increased regulatory T-cells and Th2 cells[.^13^](#_bookmark34)[^,14^](#_bookmark35) Our previous study has shown that patients with advanced melanoma have elevated Th2 cytokines that incapacitate anti-tumor immune responses[.^15^](#_bookmark36) Such a dysregulation can serve as a barrier for successful immunotherapy.

- 1. Ibrutinib

Ibrutinib is a first-in-class, potent, orally administered inhibitor of Bruton’s tyrosine kinase (BTK)[.^16^](#_bookmark37) BTK is essential in the B-cell antigen receptor (BCR) signalling cascade, which is constitutively activated in many B-cell malignancies. Inhibition of BTK blocks downstream B-cell receptor (BCR) signaling pathways and thus prevents B-cell proliferation. *In vitro* and preclinical studies have shown that ibrutinib covalently binds to the cysteine-481 amino acid of the BTK enzyme and inhibits numerous processes, including ERK signaling, NF-κB DNA binding, cytosine– phosphate–guanine (CpG)-mediated chronic lymphocytic leukemia (CLL)-cell proliferation, and tumor-cell migration.[^17^](#_bookmark38)[^,18^](#_bookmark39) At concentrations relevant to exposure levels in patients, ibrutinib demonstrates remarkable selectivity in the inhibition of B-cell receptor (BCR) signaling over T- cell receptor (TCR) signaling.[^17^](#_bookmark38)

The clinical benefit of ibrutinib was first demonstrated in a Phase 1 dose-escalation study of ibrutinib in subjects with recurrent B-cell lymphomas with ORRs ranging from 85.7% in subjects with CLL/SLL and mantel cell lymphoma (MCL) to 33.3% in subjects with diffuse large B cell lymphoma (DLBCL).[^19^](#_bookmark40) These findings were further demonstrated in subsequent Phase 2 studies in subjects with previously treated MCL or CLL/small lymphocytic lymphoma (SLL), and a randomized, comparator-controlled Phase 3 pivotal study in subjects with previously treated CLL/SLL.[^20-22^](#_bookmark41) Activity was also demonstrated in other histologies follicular lymphom (FL), DLBCL, Waldenström’s macroglobulinemia (WM), marginal zone lymphoma (MZL), multiple myeloma (MM) and with combination therapy (fludarabine, cyclophosphamide, rituximab; bendamustine + ritxumab; and ofatumumab).[^19^](#_bookmark40)[^,23^](#_bookmark42)[^,24^](#_bookmark43)

Based on positive early Phase 1 and 2 data, Ibrutinib (IMBRUVICA®) is approved by the U.S. Food and Drug Administration (FDA) for the treatment of : 1) mantle cell lymphoma (MCL) in patients who have received at least one prior therapy based on overall response rate, 2) chronic lymphocytic leukemia (CLL) in patients who have received at least one prior therapy, 3) CLL in patients with 17p deletion, 4) Waldenström’s macroglobulinemia (WM) , 5) patients with Marginal Zone Lymphoma (MZL) who require systemic therapy and have received at least one prior anti- CD20-based therapy, and 6) adult patients with cGVHD after failure of 1 or more lines of systemic therapy. For the most up to date and comprehensive nonclinical and clinical information regarding ibrutinib background, safety, efficacy, in vitro and in vivo preclinical activity, and toxicology of ibrutinib, always refer to the latest version of the ibrutinib Investigator's Brochure (IB) and/or the applicable regional labeling information.

Based on currently available data, ibrutinib has an acceptable safety profile as monotherapy and when combined with chemoimmunotherapy or immunotherapy. No maximum tolerated dose (MTD) was reached in the first-in-human (FIH) study (04753) using intermittent dosing cohorts up to 12.5 mg/kg/day and continuous dosing of 560 mg or with combination therapies (up to 560 mg).[16](#_bookmark37)[,19](#_bookmark40)

- 1. Ibrutinib and Th1/Th2 immune polarity

Chronic inflammation and proinflammatory process play important roles in malignancies, including melanoma. The abnormalities in systemic immune competence (homeostasis) have been well demonstrated in metastatic melanoma[.^13-15^](#_bookmark34)[^,25^](#_bookmark44) Successful tumor immune surveillance is mediated by Th1 cells associated with tumor-specific CD8+ T cells and proinflammatory cytokines. In contrast, a Th2-domintant immune response with production of “chronic inflammation” cytokines is related with malignancy progression and metastasis[.^26^](#_bookmark45) However, this balance between Th1 and Th2 T cells in metastatic melanoma is skewed, with a preponderance of the Th2 phenotype, which inhibits an effective anti-tumor response[.^14^](#_bookmark35)[^,15^](#_bookmark36) Overcoming this dysregulation may serve as the means for improving the success of immunotherapeutic approaches[._ENREF_27](#_bookmark46)

Recent studies revealed that in addition to BTK, ibrutinib can also specifically inhibit IL-2-inducible kinase (ITK). Ibrutinib irreversibly binds to ITK and inhibits Th2 cell differentiation and activation[.^27^](#_bookmark46)[^,28^](#_bookmark47) Given the redundant role of resting lymphocyte kinase (RLK) to ITK in Th1 and CD8 T-cells, inhibition of ITK provides a platform for preferential activation of Th1 and CD8 T-cells, and therefore skews immune polarity in favor of Th1-based immune responses. Th1/Th2 skewing as well as increased CD8 T cell responses was confirmed in mouse models treated with ibrutinib.[^27^](#_bookmark46) Ibrutinib treatment in murine models also showed ameliorated chronic graft-versus-host disease, a condition mediated by B cell, and specific CD4+ T cell subset, due to BTK and ITK inhibition^28^.

The immunomodulatory effects of ibrutinib suggest that it can potentially be used to enhance antitumor immune response in combination with other immunotherapy modality. In a recent study performed in a mouse model with ibrutinib insensitive lymphoma, the combination of anti–PD-L1 antibody and ibrutinib suppressed tumor growth^29^.

- 1. Rationale for the proposed study

Based on the observations that 1) melanoma patients frequently exhibit evidence of Th2 polarization of immune responses, 2)Th2 polarization can compromise the ability of the immune system to eradicate melanoma cells, and 3) ibrutinib skews T cell responses toward an anti-tumor Th1 phenotype; we propose to test whether the combination of ibrutinib and pembrolizumab immunotherapy leads to an improvement in the objective response rate of advanced melanoma patients over that expected by pembrolizumab alone. In so doing, we will determine whether ibrutinib therapy drives a change in Th1/Th2 immune polarity favoring Th1 anti-tumor immune responses.

As the current standard of care for metastatic melanoma, pembrolizumab is well-tolerated at 200mg/3 weeks with an acceptable adverse effects profile. Based on currently available data, ibrutinib has an acceptable safety profile as monotherapy and when combined with chemoimmunotherapy or immunotherapy. No MTD was reached in the Phase 1 study in which subjects received intermittent dosing up to 12.5 mg/kg/day (1400mg) and continuous dosing of 560 mg or with combination therapies (up to 560 mg). Based on the above safety evidence, ibrutinib 560mg continuous dosing in combination with pembrolizumab 200 mg/3 weeks was initially tested. Both of the first two patients enrolled experienced Grade 3 skin rash within a week of the first dose of pembrolizumab. This level of severity and incidence has not been observed in other malignancies treated with ibrutinib or pembrolizumab, We suspect that this is a result of immunomodulation from the combination therapy in the setting of the unique tumor microenviornent of metastatic melanoma. Based on this observation, we modified this protocol to a phase I study with an expansion cohort to find the maximum tolerated dose of ibrutinib in combination of pembrolizumab. In brief, we will use a 3+3 Phase I design with a starting dose for ibrutinib at 280 mg.

As metabolism of Ibrutinib is liver dependent, lower doses are needed for patients with pre- exsiting clinically significant liver impairment. Given the lower starting doses we have selected, the decision was made to exclude patients with pre-exsiting clinically significant liver impairment.

# Goals

- 1. Primary goals
  2. Phase I: To determine the maximum tolerated dose of ibrutinib in combination with pembrolizumab in patients with advanced melanoma.
  3. Dose Expansion Cohort: To estimate the overall response rate treated at the maximum tolerated dose of ibrutinib in combination with pembrolizumab in patients with advanced melanoma.
  4. Secondary goals
  5. To assess the safety and adverse-event profiles of combination of ibrutinib with pembrolizumab in patients with advanced melanoma.
  6. To evaluate the overall response rate (ORR) in patients with advanced melanoma receiving ibrutinib and pembrolizumab.
  7. To evaluate the duration of response, progression-free survival (PFS), and overall survival (OS) in patients with advanced melanoma receiving ibrutinib and pembrolizumab.
  8. To assess the effect of treatment with ibrutinib and pembrolizumab on Th1/Th2 immune polarity.
  9. Exploratory goals
  10. To assess the CD8 T cell response to multiple melanoma-associated antigens, and to correlate CD8 T cell responses with changes in Th1/Th2 immune polarity
  11. To assess changes in plasma cytokines induced by treatment with ibrutinib and pembrolizumab
  12. To assess the change in potential biomarkers, such as tumor-bound and soluble PD-L1 levels and tumor-infiltrating lymphocytes, that may correlate with treatment responses.

# Patient Eligibility

- 1. Pre-Registration – Inclusion Criteria
  2. Age ≥18 years of age on the day of pre-registration.
  3. Diagnosis of unresectable stage III or metastatic melanoma (stage IV) not amenable to local therapy.
  4. At least one non-nodal lesion considered measurable by RECIST criteria (that is, a lesion whose longest diameter can be accurately measured as ≥1.0 cm with CT scan, CT component of a PET/CT, or MRI) or at least one malignant lymph node is considered measurable by RECIST criteria (that is, its short axis is >1.5 cm when assessed by CT scan)

**NOTE:** Tumor lesions in a previously irradiated area are not considered measurable disease.

- 1. ECOG Performance Status (PS) 0, 1, or 2 (Appendix II)
  2. Provide informed written consent.
  3. Patient is willing to undergo treatment and monitoring at the enrolling institution.
  4. Willing to provide tissue and blood samples for correlative research purposes (see Sections 6.12, 14.0 and 17.0*).*
  5. Pre-Registration – Exclusion Criteria
  6. Any of the following:
- Pregnant women
- Nursing women
- Men or women of childbearing potential who are unwilling to employ adequate contraception within the projected duration of the study, starting with the screening visit through 120 days after the last dose of study medication. Adequate contraception is defined as 2 methods of birth control (e.g., hormonal contraceptives, intrauterine device, diaphragm with spermicide, cervical cap with spermicide, male condoms, or female condom with spermicide) or prior surgical sterilization, or abstinence from heterosexual activity
  1. Prior treatment with ibrutinib or prior exposure to BTK inhibitors.
  2. Uveal melanoma.
  3. Current use of warfarin or other Vitamin K antagonists.
  4. Require continuous treatment with a strong CYP3A inhibitor (see Appendix V).
  5. Currently participating or has participated in a study of an investigational cancer therapy agent or using an investigational device within 28 days prior to pre- registration.
  6. Live vaccines within 28 days prior to pre-registration.
  7. Invasive surgical procedure within 28 days prior to pre-registration.
  8. a History of clinically severe (e.g., requires chronic immunosuppressive therapy, [e.g., cyclosporine A, tacrolimus]) autoimmune disease (e.g., ulcerative colitis, lupus), or history of organ transplant.

3.29b Known history of HIV infection, active infection with hepatitis B virus or hepatitis C virus, or any uncontrolled active systemic infection.

3.29c Gastrointestinal disease that might inhibit ibrutinib absorption (e.g., malabsorption syndrome, resection of the stomach or a large portion of small bowel, or partial/complete bowel obstruction), or unable to swallow capsules.

3.29d Active central nervous system metastases and/or carcinomatous meningitis.

**Note**: Patients with untreated brain metastasis will be excluded. Patients with previously treated brain metastases may participate provided they meet the following criteria:

- Inactive (without evidence of progression which is documented by CT or MRI within 90 days prior to pre-registration), AND
- On ≤10 mg/day prednisone or equivalent for at least 28 days prior pre- registration.

3.29e Co-morbid systemic illnesses or other severe concurrent disease which, in the judgment of the investigator, would make the patient inappropriate for entry into this study or interfere significantly with the proper assessment of safety and toxicity of the prescribed regimens.

3.29f Clinically significant cardiovascular disease such as unstable angina, myocardial infarction, or acute coronary syndrome within ≤180 days prior to pre- registration, symptomatic or uncontrolled arrhythmia, congestive heart failure, or any Class 3 or 4 cardiac disease as defined by the New York Heart Association Functional Classification.

3.29g Other active malignancy ≤3 years prior to pre-registration. Note: If there is a history of prior malignancy, the patient must not be receiving other specific treatment for cancer. EXCEPTIONS: Non-melanotic skin cancer or carcinoma- in-situ of the cervix.

3.29h Currently active, clinically significant cardiovascular disease, such as uncontrolled arrhythmia or Class 3 or 4 congestive heart failure as defined by the New York Heart Association Functional Classification; or a history of myocardial infarction, unstable angina, or acute coronary syndrome ≤6 months prior to pre-randomization.

3.29i Known bleeding disorders (von Wilebrand’s disease or hemophilia)

3.29j History of ischemic stroke or intracranial hemorrhage ≤180 days prior to pre- registration.

3.29k Currently active, clinically significant hepatic impairment Child-Pugh class A, B or C according to the Child Pugh classification (see Appendix VII).

3.29l Unresolved toxicities from prior anti-cancer therapy, defined as not resolved to Common Terminology Criteria for Adverse Events (CTCAE, v4.0) Grade 0 or 1, or to the levels dictated in the inclusion/exclusion criteria with the exceptions of alopecia and peripheral neuropathy.

- 1. Registration – Inclusion Criteria
  2. Histologic or cytologic confirmation of unresectable stage III or metastatic melanoma (stage IV) not amenable to local therapy.
  3. Only if patient has had previous exposure to anti-PD-1 or anti-PD-L1 therapy:
     1. Patient had disease progression on or within 6 months after anti-PD-1/anti-PD- L1 therapy in the metastatic setting

**OR**

- - 1. Patient had disease progression within 6 months after the last dose of adjuvant/neoadjuvant anti-PD-1/anti-PD-L1 treatment
  1. The following laboratory values obtained ≤14 days prior to registration.
- Absolute neutrophil count (ANC) ≥1000/mm^3^
- Platelet count ≥75,000/mm^3^*
- Hemoglobin ≥9.0 g/dL
- Total bilirubin ≤1.5 X upper limit of normal (ULN); if total bilirubin >1.5 X ULN then direct bilirubin ≤ ULN
- Aspartate transaminase (AST) and alanine transaminase (ALT) ≤2.5 x ULN

**OR** ≤5 X ULN for patients with liver metastases

- Creatinine ≤1.5 X ULN and CrCL ≥30 ml/min per Cockcroft Gault formula


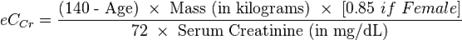


*Criteria must be met without a transfusion ≤four weeks prior to registration.

- 1. Patients of childbearing potential only: negative urine pregnancy test done

≤7 days prior to study registration.

- 1. Registration – Exclusion Criteria
  2. Failure to confirm histologically or cytologically unresectable stage III or metastatic melanoma (stage IV) not amenable to local therapy.
  3. Prior chemotherapy, immunotherapy, radioactive, or biological cancer therapy (including mAb) ≤28 days prior to registration.
  4. Received a strong cytochrome P450 (CYP) 3A inhibitor ≤7 days prior to registration
  5. Concurrent systemic immunosuppressant therapy ≤21 days prior to registration.
  6. Recent infection requiring systemic antibiotic treatment that was completed ≤14

days prior to registration.

# Test Schedule

| Treatment Cycle | Pre- registration | ≤14 days prior to  regist- ration | Active Monitoring Phase | | | | | |
| --- | --- | --- | --- | --- | --- | --- | --- | --- |
|  |  |  | Cycles 1-4^1^ | | | | Day 1 Subsequent cycles [21- day cycle] | At confirmed disease progression^2^ |
| Tests and procedures |  |  | C1D8 (±1  day) | C2D1 (±3  days) | C3D1 (±3  days) | C4D1 (±3  days) | (± 3 days) |  |
| HLA Class I Typing^R^ |  | X |  |  |  |  |  |  |
| ECOG performance status |  | X | X | X | X | X | X | X |
| History, physical exam, vital signs^3^, and weight |  | X | X | X | X | X | X | X |
| Child Pugh Score |  | X |  | X | X | X | X | X |
| Height |  | X |  |  |  |  |  |  |
| Adverse event assessment |  | X | X | X | X | X | X | X |
| Urine Pregnancy test |  | X4 |  |  |  |  |  |  |
| CBC with Differential:^5^ |  | X6 | X | X | X | X | X | X |
| Chemistry group: AST, ALT,  Bilirubin (total and direct), Creatinine, albumin, INR, LDH |  | X6 | X | X | X | X | X | X |
| TSH |  | X7 |  |  | X |  | X7 | X |
| Tumor imaging and measurement ^,8^ |  | X8 |  |  |  |  | X9 | X |
| Research blood specimens (see Section 14.0)^10,R^ |  | X | X | X | X | X | X10 | X |
| Tissue specimens (see Section 17.0)^,R^ | X11 |  |  |  |  |  | X12 | X12 |
| BRAF V600E and V600K  mutation testing |  | X |  |  |  |  |  |  |

- - 1. Cycle 1= 28 day, Subsequent Cycles=21 days.
    2. Confirmed progression is an objective status of progression on two consecutive evaluations at least 6 weeks apart.
    3. Vital signs must include blood pressure and heart rate.
    4. For women of childbearing potential only. Must be done ≤7 days prior to registration.
    5. CBC differential must include hemoglobin, platelets, absolute neutrophil count, absolute lymphocyte count, and absolute monocyte count
    6. CBC with differential and chemistry groups must be done ≤14 days prior to registration.
    7. TSH testing is to be performed ≤14 days prior to registration. And at the end of each even numbered cycle ( 2, 4, 6, 8, 10, etc.) until confirmed progression. If TSH is abnormal, T3 and free T4 testing should be performed.
    8. The initial tumor imaging (either CT or MRI, with preference for CT) will be performed ≤28 days prior to registration. The same imaging technique will be used in a patient throughout the study. If an excisional biopsy of a target lesion is to be done, baseline tumor imaging must be performed after the biopsy is done.
    9. Imaging is to be performed at the end of Cycle 4, 6, 8, 10, and 12. If a patient has not had a confirmed disease progression after Cycle 12, subsequent scans will take place at the end of every 3^rd^ cycle of treatment thereafter (that is, at the end of Cycle 15, 18, 21 until confirmed disease progression). See Section 11.
    10. Mandatory research blood specimen will be collected at Cycle 1 Day 8 and at the beginning of each cycle for Cycles 2-4, then at the beginning of every cycle when tumor imaging is performed until confirmed progression or at treatment discontinuation if not for PD.
    11. Mandatory core needle biopsy. A newly obtained tumor biopsy is required, unless tissue is

available for research use from a prior biopsy obtained ≤90 days prior to registration.

- - 1. Mandatory tissue/biopsy is required at the time of the first disease assessment (the evaluation at the end of Cycle 4). Optional tissue/biopsy will be obtained at the time of confirmed progression. Prior to and following biopsy hold ibrutinib per section 8.4.

R Research funded (see Section 19.0)

# Grouping Factors

None

# Registration Procedures

- 1. Pre-Registration

**Call the Registration Office (507-284-2753) prior to discussing protocol entry with the patient to ensure that a place on the protocol is open to the patient.**

The lag between pre-registration and registration should be no more than 28 days.

- 1. Pre-registration

To pre-register a patient, fax (507-284-0885) a completed eligibility checklist to the Mayo Clinic Cancer Center (MCCC) Registration Office between 8 a.m. and 4:30 p.m. central time Monday through Friday.

- 1. IRB approval(s) is required for each treating site.

In addition to submitting initial IRB approval documents, ongoing IRB approval documentation must be on file (no less than annually). If the necessary documentation is not submitted in advance of attempting patient registration, the registration will not be accepted and the patient may not be enrolled in the protocol until the situation is resolved.

When the study has been permanently closed to patient enrollment, submission of annual IRB approvals is no longer necessary.

- 1. Verifications

Prior to accepting the pre-registration, the registration/randomization application will verify the following:

- - - IRB approval at the registering institution
    - Patient pre-registration eligibility
    - Existence of a signed consent form
    - Existence of a signed authorization for use and disclosure of protected health information
  1. Pre-registration tests/procedures

Pre-registration tests/procedures (see Section 4.0) must be completed within the guidelines specified on the test schedule.

- 1. Correlative Research

A mandatory correlative research component (tissue and blood) is part of this study; the patient will be automatically pre-registered onto this component (see Sections 3.17 and 14.2).

An optional correlative research component (tissue) is part of this study; there will be an option to select if the patient is to be pre-registered onto this component (see Section 17.32).

- - - Patient has/has not given permission to give his/her tissue sample for research testing.
  1. Other permissions

At the time of pre-registration, the following will be recorded:

- - - Patient has/has not given permission to store and use his/her sample(s) for future research of melanoma at Mayo.
    - Patient has/has not given permission to store and use his/her sample(s) for future research to learn, prevent, or treat other health problems.
    - Patient has/has not given permission for MCCC to give his/her sample(s) to researchers at other institutions.
  1. Registration Procedures

**Prior to discussing protocol entry with the patient, call the MCCC Registration Office (507-284-2753) for dose level and to insure that a place on the protocol is open to the patient.**

- 1. To register a patient, fax (507-284-0885) a completed eligibility checklist to the Mayo Clinic Cancer Center (MCCC) Registration Office between 8 a.m. and 4:30 p.m. central time Monday through Friday.
  2. Documentation of IRB approval must be on file in the Registration Office before an investigator may register any patients.

In addition to submitting initial IRB approval documents, ongoing IRB approval documentation must be on file (no less than annually) at the Registration Office (fax: 507-284-0885). If the necessary documentation is not submitted in advance of attempting patient registration, the registration will not be accepted and the patient may not be enrolled in the protocol until the situation is resolved.

When the study has been permanently closed to patient enrollment, submission of annual IRB approvals to the Registration Office is no longer necessary.

- 1. Prior to accepting the registration, the registration/randomization application will verify the following:
     - IRB approval at the registering institution
     - Patient eligibility
     - Existence of a signed consent form
     - Existence of a signed authorization for use and disclosure of protected health information
  2. Treatment on this protocol must commence at Mayo Clinic Rochester under the supervision of a medical oncologist.
  3. Treatment cannot begin prior to registration.
  4. Pretreatment tests/procedures (see Section 4.0) must be completed within the guidelines specified on the test schedule.
  5. All required baseline symptoms (see Section 10.6) must be documented and graded.
  6. Study drug is available on site.

# Protocol Treatment

- 1. Treatment Schedule

Use actual weight or estimated dry weight if fluid retention Cycle 1 treatment = 28 days

All subsequent treatment cycles = 21 days (±3 days)

| Drug | Dose | Treatment | Retreatment |
| --- | --- | --- | --- |
| Ibrutinib | assigned at registration | PO daily (Day 1 to 28  of Cycle 1, then Day 1- 21* on subsequent cycles) | Every 21 days  (±3 days)  until confirmed progression |
| Pembrolizumab | 200 mg | IV injection on Day 8 of Cycle 1 and then Day 1 of each  subsequent cycle | Every 21 days (±3 days) until confirmed progression |

* Patients should receive a sufficient quantity of ibrutinib to last until the next scheduled treatment visit.

- 1. Determination of Maximum Tolerated Dose (MTD)
  2. Dose Escalation Scheme

| Dose level | Ibrutinib** |
| --- | --- |
| -1 | 140 mg |
| 0* | 280 mg |
| 1 | 420 mg |

* Starting dose level

** If the patient has clinical or laboratory evidence of liver failure during the treatment, then calculate the Child-Pugh score and adjust the ibrutinib dose (see Table 8.3).

NOTE: Doses will not be escalated in any individual patient.

- 1. MTD Determined

As of MCCC Amendment 6, the MTD has been determined to be Dose Level 1. Dose Level 1 will be used for the expansion cohort.

- 1. Dose Limiting Toxicities

Dose limiting toxicities (DLT) are defined to be:

| ***Toxicity*** | ***DLT Definition*** |
| --- | --- |
| Hematologic | Grade 4 ANC, Grade 4 Hgb, or PLT <25,000 |
| Renal | Serum creatinine ≥2 times baseline |
| Neurologic | ≥Grade 2 neurosensory or neuromotor toxicity |
| Other nonhematologic | ≥Grade 3 per NCI Common Terminology Criteria for Adverse Events (CTCAE) version 4.0* |

*Grade 3 rash or fever despite maximal supportive treatment(s) will be considered dose-limiting; all grade 4 rash and fever will be considered a DLT.

The maximum tolerated dose is defined as the highest dose level among those tested where at most one out of 6 patients develops a DLT prior to the start of their second cycle of treatment and the next highest dose level is such that 2 out of a maximum of 6 patients treated at this dose level developed a DLT prior to the start of their second cycle of treatment.

- 1. Dose escalation plan

NOTE: As of MCCC Amendment 6, the MTD has been determined to be Dose Level 1. A minimum of 2 or a maximum of 6 patients will be accrued to a given dose level.

For all dose levels, patients will be accrued so that at any given time no more than 2 patients will be receiving their first cycle of treatment **and** acute adverse event data over the first treatment cycle for all other patients treated at the current dose level is known.

If, at any time in the enrollment process, 2 patients treated at the current dose level develop a DLT during the first cycle of treatment, enrollment will be closed to that dose level. Enrollment will be re-opened to the next lower dose level if fewer than 6 patients have been treated at that dose level.

If none of the first 3 patients treated at a given dose level develops a DLT during the first cycle of treatment, enrollment to the dose level will be closed and enrollment will reopen at next higher dose level. If there are no other higher dose levels to be tested, three additional patients will enrolled at the current dose level to confirm MTD.

If one of the first 3 patients treated at a given dose level develops a DLT during the first cycle of treatment, three additional patients will enrolled (sequentially) on that dose level.

If, at any time in the enrollment of these 3 additional patients, a patient develops a DLT, enrollment will be closed to that dose level. Enrollment will be re-opened to the next lower dose level if fewer than 6 patients have been treated at that dose level.

If none of these 3 additional patients develops a DLT during the first cycle of treatment, enrollment to that dose level will be closed and enrollment will reopen at next higher dose level. If there are no other higher dose levels to be tested, this will be considered the MTD.

- 1. Replacement

If a patient fails to complete the first cycle of treatment for reasons other than toxicity, an additional patient will be enrolled to replace this patient. For these instances, a specific notation will be made for review by the Scientific Progress Review Committee.

- 1. Return to consenting institution

The patient must return to the consenting institution for evaluation and treatment at least every 21 days (± 3 days) during treatment.

# Dosage Modification Based on Adverse Events

Strictly follow the modifications in this table for the first cycle, until individual treatment tolerance can be ascertained. Thereafter, these modifications should be regarded as guidelines to produce mild-to-moderate, but not debilitating, side effects. If multiple adverse events are seen, administer dose based on greatest reduction required for any single adverse event observed. Reductions or increases apply to treatment given in the preceding cycle and are based on adverse events observed since the prior dose.

***ALERT:* ADR reporting may be required for some adverse events (See Section 10)**

- 1. Dose Levels

(Based on Adverse Events in Table 8.2)

| **Dose Level** | **Ibrutinib** | **Pembrolizumab** |
| --- | --- | --- |
| 1* | 420 mg PO once daily | 200 mg IV |
| -1 | 280 mg PO once daily | NA |
| -2 | 140 mg PO once daily | NA |

*Dose level 1 refers to the starting dose.

- 1. Dose Modifications Based on Adverse Events

NOTE: These modifications only apply if the adverse event is **possibly**, **probably**, or

**definitely** related to study treatment

| **System/Organ/Class (SOC)** | **ADVERSE EVENTS/SYMPTOMS** | **ACTIONS** | |
| --- | --- | --- | --- |
|  |  | **Pembrolizumab** | **Ibrutinib** |
| Blood and lymphatic | **Anemia** | | |
| system disorders | Grade 4 | Hold pembrolizumab until  ≤Grade 2 and etiology is  determined  If anemia is immune-mediated (at least possibly related to pembrolizumab), discontinue pembrolizumab | Discontinue ibrutinib |
|  | **Febrile neutropenia (See also Investigations/Neutrophil count decreased)** | | |
|  | Grade 3 | Hold until symptoms resolve to  ≤Grade 1 | Hold until symptoms resolve to  ≤Grade 1;  Resume at the next lower dose level (if occurs at 140 mg once daily, discontinue) |
|  | Grade 4 | Hold until symptoms resolve to  ≤Grade 1 | Discontinue ibrutinib |
| Cardiac disorders | **Cardiac arrythmias** | | |
|  | Grade 3 | Continue pembrolizumab | Hold until symptoms resolve to  ≤Grade 1, then resume Resume at the next lower dose level  (if occurs at 280 mg once daily or lower, discontinue ibrutinib) |
|  | Grade 4 | Discontinue and enter Event Monitoring. | Discontinue and enter Event Monitoring |

| **System/Organ/Class (SOC)** | **ADVERSE EVENTS/SYMPTOMS** | **ACTIONS** | |
| --- | --- | --- | --- |
|  |  | **Pembrolizumab** | **Ibrutinib** |
| Cardiac disorders | **Cardiac failure** (not due to immune-related myocarditis) | | |
|  | Grade 2 | Hold until symptoms resolve to  ≤Grade 1, then resume | Hold until symptoms resolve to  ≤Grade 1;  Resume at the next lower dose level (if occurs at 140 mg once  daily, discontinue) |
|  | Grade 3 or 4 | Discontinue and enter Event Monitoring. | Discontinue and enter Event Monitoring. |
| Cardiac disorders | **Myocarditis** | | |
|  | Grade 2+ | Discontinue and enter Event Monitoring. | Discontinue and enter Event Monitoring. |
| Endocrine Disorders | **Symptomatic Hypophysitis** | | |
|  | Grade 2 and 3 | Hold until symptoms resolved to ≤Grade 1, initiate systemic steroid (see Section 9.6) and physiologic replacement dose. Discontinue if does not resolve to ≤Grade 1 within 12 weeks after last dose of  pembrolizumab, and then enter Event Monitoring | Hold until pembrolizumab resumed. Resume at the next lower dose level (if occurs at 140 mg once daily, discontinue).  Discontinue if pembrolizumab is discontinued, and enter Event Monitoring. |
|  | Grade 4 | Discontinue and enter Event Monitoring. Initiate systemic steroid (see Section 9.6) and physiologic replacement dose | Discontinue and enter Event Monitoring. |
|  | **Hypothyroidism** | | |
|  |  | Isolated hypothyroidism may be managed with replacement therapy without treatment  interruption and without corticosteroids | Continue. |
|  | **Hyperthyroidism** | | |
|  | Grade 3 | Hold until symptoms resolved to ≤Grade 1, initiate systemic steroid (see Section 9.6) Discontinue if does not resolve to ≤ Grade 1 within 12 weeks after last dose administration, and enter Event Monitoring | Hold until pembrolizumab resumed  Resume at the next lower dose level (if occurs at 140 mg once daily, discontinue).  Discontinue if pembrolizumab is discontinued, and enter Event Monitoring |
|  | Grade 4 | Discontinue and enter Event Monitoring. Initiate systemic steroid treatment | Discontinue and enter Event Monitoring |
| Gastrointestinal Disorders | **Diarrhea** | | |
|  | Grade 2 with symptoms  <1 week | *Hold until symptoms resolved to ≤Grade 1. Discontinue if does not resolve to ≤Grade 1 within 12 weeks after last dose of pembrolizumab, and then enter Event Monitoring | Hold until pembrolizumab resumed  Resume at the next lower dose level (if occurs at 140 mg once daily, discontinue).  Discontinue if pembrolizumab  is discontinued, and enter Event Monitoring |

| **System/Organ/Class (SOC)** | **ADVERSE EVENTS/SYMPTOMS** | **ACTIONS** | |
| --- | --- | --- | --- |
|  |  | **Pembrolizumab** | **Ibrutinib** |
|  | Grade 2 with symptoms  >1 week or Grade 3 | *Hold until symptoms resolved to ≤Grade 1, initiate systemic steroid (see Section 9.6) Discontinue if does not resolve to ≤Grade 1 within 12 weeks after last dose administration, and enter Event Monitoring | Hold until pembrolizumab resumed  Resume at the next lower dose level (if occurs at 140 mg once daily, discontinue).  Discontinue if pembrolizumab is discontinued, and enter Event Monitoring |
|  | Grade 4 | Discontinue and enter Event Monitoring  Initiate systemic steroid treatment (see Section 9.6) | Discontinue and enter Event Monitoring |
|  | **Nausea and Vomiting** | | |
|  | Grade ≥3 despite optimal  anti-emetic therapy | Continue if Grade 3  Hold if >Grade 3 until resolves  to ≤Grade 1 | Hold  Resume according to Table 8.2 |
| Investigations | Hepatic Dysfunction (**AST** or **ALT** or total **bilirubin**) and immune-mediated hepatitis | | |
|  | Grade 2  (AST or ALT >3 × and  <5 × ULN  or total bilirubin >1.5 × and <3 × ULN) | *Hold until levels resolve to  ≤Grade 1  (AST or ALT ≤3× ULN,  total bilirubin ≤1.5 × ULN)  Consider initiating systemic steroid treatment (see Section 9.6)  Discontinue if does not resolve to ≤Grade 1 within 12 weeks after last dose administration,  and enter Event Monitoring | Hold until pembrolizumab resumed  Resume at the next lower dose level (if occurs at 140 mg once daily, discontinue).  Discontinue if pembrolizumab is discontinued, and enter Event Monitoring |
|  | Grade 3  (AST or ALT >5 × ULN or  total bilirubin >3 ×ULN) or for patients with liver metastasis who begin treatment with Grade 2 AST or ALT, if AST or ALT increases by greater than or equal to 50%  relative to baseline and lasts ≥1 week | Discontinue and enter Event Monitoring  Initiate systemic steroid treatment (see Section 9.6) | Discontinue and enter Event Monitoring. |
| Immune System Disorders | **Immune-related Adverse Events** | | |
|  | Grade 2 | *Hold until symptoms resolved to ≤Grade 1, initiate systemic steroid (see 9.6). Discontinue if does not resolve to ≤Grade 1 within 12 weeks after last dose administration, and enter Event Monitoring. | Hold until pembrolizumab resumed. Resume at the next lower dose level (if occurs at 140 mg once daily, discontinue).  Discontinue if pembrolizumab is discontinued, and enter Event Monitoring |
|  | Grade ≥3 (except gastrointestinal and endocrine disorders) | Discontinue and enter Event Monitoring  Initiate systemic steroid treatment | Discontinue and enter Event Monitoring |

| **System/Organ/Class (SOC)** | **ADVERSE EVENTS/SYMPTOMS** | **ACTIONS** | |
| --- | --- | --- | --- |
|  |  | **Pembrolizumab** | **Ibrutinib** |
| Infections and | **Infectious Disorders** | | |
| infestations | Grade ≥3 | *Hold  If ibrutinib is held resume if  ≤Grade 1 or baseline | Hold until ≤Grade 1 or  baseline  Resume at the next lower dose level (if occurs at 140 mg once  daily, discontinue). |
| Injury, poisoning and | **Infusion-related Reaction** | | |
| procedural complications | Grade ≥3 | Discontinue and enter Event Monitoring | Discontinue and enter Event Monitoring |
| Investigations | **Neutrophil count decreased (See also Blood and lymphatic systems disorders/Febrile neutropenia)** | | |
|  | Grade ≥3 neutropenia with  infection or  Grade 4 neutropenia | *Hold until ≤Grade 2 Discontinue if does not resolve to ≤Grade 2 within 12 weeks  after last dose administration, and enter Event Monitoring. | Hold until ≤Grade 1 or baseline  Resume at the next lower dose level (if occurs at 140 mg once  daily, discontinue). |
|  | **Platelet count decreased** | | |
|  | Platelet counts <25 x 10^9^/L or platelet counts <50 x 10^9^/L with clinically significant bleeding events | *Hold until ≤Grade 2 Discontinue if does not resolve to ≤Grade 2 within 12 weeks  after last dose administration, and enter Event Monitoring | Hold until ≤Grade 1 or baseline  Resume at the next lower dose  level (if occurs at 140 mg once daily, discontinue). |
| Renal and urinary disorders | **Acute kidney injury** | | |
|  | Nephritis Grade 2 | *Hold until symptoms resolved to ≤Grade 1, initiate systemic steroids (see 9.6). Discontinue if does not resolve to ≤Grade 1 within 12 weeks after last dose administration, and enter Event Monitoring | Hold until pembrolizumab is resumed  Resume at the next lower dose level (if occurs at 140 mg once daily, discontinue).  Discontinue if pembrolizumab is discontinued, and enter  Event Monitoring |
|  | Grade ≥3 | Discontinue and enter Event Monitoring | Discontinue and enter Event Monitoring |
| Respiratory, thoracic, and mediastinal disorders | **Pneumonitis** | | |
|  | Grade 2 | *Hold until symptoms resolved to ≤Grade 1, initiate systemic steroids  Discontinue if does not resolve to ≤Grade 1 within 12 weeks after last dose administration, and enter Event Monitoring | Hold until pembrolizumab resumed  Resume at the next lower dose level (if occurs at 140 mg once daily, discontinue).  Discontinue if pembrolizumab is discontinued, and enter Event Monitoring |
|  | Grade ≥3 | Discontinue and enter Event Monitoring | Discontinue and enter Event Monitoring |
| Skin and subcutaneous tissue disorders | **Rash maculo-papular** | | |
|  | Grade 2 | *Hold pembrolizumab until resolves to ≤Grade 1 Discontinue if does not resolve to ≤Grade 1 within 12 weeks after last dose administration | Hold until pembrolizumab is resumed  Resume at the next lower dose level (if occurs at 140 mg once daily, discontinue).  Discontinue if pembrolizumab  is discontinued, and enter Event Monitoring |

| **System/Organ/Class (SOC)** | **ADVERSE EVENTS/SYMPTOMS** | **ACTIONS** | |
| --- | --- | --- | --- |
|  |  | **Pembrolizumab** | **Ibrutinib** |
|  | Grade ≥3 | Hold pembrolizumab. If resolves to ≤Grade 1 within 72 hours resume treatment  If not, discontinue and enter Event Monitoring | Hold until pembrolizumab resumed  Resume at the next lower dose level (if occurs at 140 mg once daily, discontinue).  Discontinue if pembrolizumab is discontinued, and enter Event Monitoring |
| Other non-laboratory Adverse Events |  | | |
|  | Grade 3 | *Hold pembrolizumab until resolves to ≤Grade 1 Discontinue if does not resolve to ≤Grade 1 within 12 weeks after last dose administration | Hold until pembrolizumab resumed Resume at the next lower dose level (if occurs at 140 mg once daily, discontinue).  Discontinue if pembrolizumab is discontinued, and enter Event Monitoring |
|  | Grade 4 | Discontinue and enter Event Monitoring | Discontinue and enter Event Monitoring |

*If ibrutinib is discontinued according to Table 8.2, continue pembrolizumab until disease progression or intolerance develops or patient refusal.

- 1. Modifications Based on Concomitant Medications and Procedures for Ibrutinib
     - **Dose Modifications for Use with CYP3A Inhibitors and Inducers**

Ibrutinib is metabolized primarily by CYP3A. Avoid co-administration with strong CYP3A4 or moderate CYP3A inhibitors and consider alternative agents with less CYP3A inhibition.

- - - - If a strong CYP3A inhibitor (eg, ketoconazole, posaconazole, indinavir, nelfinavir, ritonavir, saquinavir, clarithromycin, telithromycin, itraconazole, nefazadone, or cobicistat) must be used, reduce ibrutinib to 140 mg for the duration of the inhibitor or withhold ibrutinib treatment temporarilty (for 7 days or less). Pembrolizumab may be continued at current dose. If unable to discontinue strong CYP3A inhibitor use within 12 weeks then permanently discontinue ibrutinib.
      - If a moderate CYP3A inhibitor (eg, erythromycin, amprenavir, aprepitant, atazanavir, ciprofloxacin, crizotinib, diltiazem, fluconazole, voriconazole, fosamprenavir, imatinib, verapamil, amiodarone, or dronedarone) must be used, reduce ibrutinib to 280 mg once daily for the duration of the inhibitor use. Avoid grapefruit and Seville oranges during ibrutinib treatment, as these are moderate inhibitors of CYP3A. (See Appendix V.)
      - No dose adjustment is required in combination with mild inhibitors.

Avoid concomitant use of system strong CYP3A inducers (e.g. carbamazepine, rifampin, phenytoin and St. John’s wort) should be avoided. Consider alternative medications with less CYP3A induction.

A list of common CYP3A inhibitors and inducers is provided in Appendix V. A comprehensive list of inhibitors, inducers, and substrates may be found at [http://medicine.iupui.edu/clinpharm/ddis/main-table/.](http://medicine.iupui.edu/clinpharm/ddis/main-table/) This website is continually revised and should be checked frequently for updates.

For the most comprehensive effect of CYP3A inhibitors or inducers on ibrutinib exposure, please refer to the current version of the IB.

- - - Concurrent use with QT Prolongation Agents

Ibrutinib is not known to prolong the QT interval, but it may affect the pharmacokinetics of other medications. Any medications known to cause QT prolongation should be used with caution; periodic ECG and electrolyte monitoring should be considered.

- - - **Dose modification for bleeding-related events (Antiplatelet Agents and Anticoagulants)**

Use ibrutinib with caution in subjects requiring anticoagulants or medications that inhibit platelet function. In an in vitro platelet function study, inhibitory effects of ibrutinib on collagen-induced platelet aggregation were observed. Supplements such as fish oil and vitamin E preparations should be avoided during treatment with ibrutinib. Bleeding events of any grade, including bruising and petechiae, occurred in subjects treated with ibrutinib. Ibrutinib should be held at least ~~3 to~~ 7 days pre- and post-surgery depending upon the type of surgery and the risk of bleeding (see section

on Dose modification for invasive procedures or surgery). Subjects with congenital bleeding diathesis have not been studied.

Subjects requiring the initiation of therapeutic anticoagulation therapy (eg, atrial fibrillation) should be monitored closely for signs and symptoms of bleeding and the risks and benefits of continuing ibrutinib treatment should be considered.

- - - **Dose modification for invasive procedures or surgery**

Ibrutinib may increase risk of bleeding with invasive procedures or surgery. The following guidance should be applied to the use of ibrutinib in the perioperative period for subjects who require surgical intervention or an invasive procedure while receiving ibrutinib:

- - - - For minor procedures (such as a central line placement, skin or needle biopsy, lumbar puncture [other than shunt reservoir access], thoracentesis, or paracentesis) ibrutinib should be held for at least 3 days prior to the procedure and should not be restarted at least 3 days after the procedure. For bone marrow biopsies, that are performed while the subject is on ibrutinib, it is not necessary to hold ibrutinib.
      - For any surgery or invasive procedure requiring sutures or staples for closure, ibrutinib should be held at least 7 days prior to the intervention (except for emergency procedures) and should be held at least 7 days after the procedure and restarted at the discretion of the investigator when the surgical site is reasonably healed without serosanguineous drainage or the need for drainage tubes.
    - **Dose modification for liver impairment subjects**

Ibrutinib is metabolized in the liver and therefore subjects with clinically significant hepatic impairment (Child- Pugh class B or C) are excluded from study participation in our initial phase 2 study, and lower starting dose was given in subjects with Child - Pugh Class A liver impairment (280 mg daily).

In this modified phase 1 study, patients with clinical evidence of liver impairement (Child – Pugh Class A, B, and C) at the time of screening are already excluded from particitipating, in addition to a lowered starting dose at 420 mg daily (compared to 520 mg in the initial study), to reduce any significant adverse effects. Therefore, for any liver impairment during the course of the treatment, the dose modification will follow the guideline in table 8.1 and 8.2, and no further modifications will be needed. It is critical for providers to monitor subjects for signs of toxicity and follow dose modification guidance as needed (Refer to Appendix 4 Table 8.1).

- For subjects with existing chronic mild hepatic impairment (Child-Pugh class A) at enrollment, the starting dose has to be adjusted to a level of 280 mg daily (two capsules).
- For subjects who develop mild hepatic impairment while on study (Child-Pugh class A), the recommended dose reduction for ibrutinib/placebo is to a level of 280 mg daily (two capsules) unless lower doses had already been implemented.
- For subjects who develop moderate hepatic impairment while on study (Child-Pugh class B), the recommended dose reduction is to a level of 140 mg daily (one capsule).
  1. Overdose
- Subjects who develop severe hepatic impairment (Child-Pugh class C) must hold study drug until resolved to moderate impairment (Child-Pugh class B) or better.

Subjects who develop acute hepatic toxicity with liver enzymes Grade 3 or higher while on study should be managed per standard dose modification guidelines in Section 8.

There is no specific experience in the management of ibrutinib overdose in patients. No maximum tolerated dose (MTD) was reached in the Phase 1 study in which subjects received up to 12.5 mg/kg/day (1400 mg/day). Healthy subjects were exposed up to single dose of 1680 mg. One healthy subject experienced reversible Grade 4 hepatic enzyme increases (AST and ALT) after a dose of 1680 mg. Subjects who ingest more than the recommended dosage should be closely monitored and given appropriate supportive treatment.

Refer to Section 10.0 for further information regarding AE reporting.

# Ancillary Treatment/Supportive Care

- 1. Full supportive care

Patients should receive full supportive care while on this study. This includes blood product support, antibiotic treatment, and treatment of other newly diagnosed or concurrent medical conditions. All blood products and concomitant medications such as antidiarrheals, analgesics, and/or antiemetics received from the first day of study treatment administration until 28 days after the final dose will be recorded in the medical records.

- 1. Hypersensitivity reactions

Patients do not require premedication prior to ibrutinib or Pembrolizumab treatment, as hypersensitivity reactions is not expected. In the unlikely event of a hypersensitivity reaction, treatment with antihistamines, H2 blockers, and corticosteroids is recommended. Patients should be pre-medicated with the typical regimen for subsequent cycles.

- 1. Concurrent enrollment in other trials

Patients may not enroll in a different clinical study, including Cancer Control studies, in which investigational procedures or agents are being used, while participating in this study.

- 1. Palliative radiation

Patients must terminate study treatment if they are to receive radiation therapy for palliative reasons as it impacts upon assessing response.

- 1. General patient monitoring and supportive care guidelines
  2. Patients should be carefully monitored during the treatment phase and then followed appropriately. Decisions for retreatment or dose modifications/interruption should follow the guidelines in Sections 8.2.
  3. Patients who have an ongoing study agent-related serious adverse event upon study completion or at discontinuation from the study will be contacted by the treating physician or his/her designee at least every 2 weeks until the event is resolved or determined to be irreversible.
  4. Side effect management for **PEMBROLIZUMAB** toxicities

These are to be regarded as guidelines for managing toxicity that occurs with pembrolizumab therapy and should not replace clinical judgement (i.e. - patients with Trade 1 rash may require systemic steroids).

- 1. Diarrhea

9.611 Grade 1- without abdominal pain/or blood in stool and symptoms, may be caused either by pembrolizumab or by ibrutinib. Infectious etiologies should be ruled out. Patients may be managed symptomatically, including the use of loperamide. Instruct patients to report any increase in stools.

9.612 Grade 2- without abdominal pain/or blood in stool and symptoms **< 1 week**, and resolve to grade 0 or 1- continue to monitor. Infectious etiologies should be ruled out.

9.613 Grade 2- symptoms **>1 week**, should be started on steroid therapy – first choice is budesonide at 12 mg once daily (if unable to obtain budesonide and/or patent continues to have diarrhea after 72 hours of use start systemic steroids at0.5 mg/kg/day prednisone or equivalent- can be given in two doses- especially for patients that have nocturnal diarrhea).** Infectious etiologies should be ruled out.

9.614 Grade 3 or greater- **who have other etiologies ruled out** should be started on systemic steroids at 1-2 mg/kg/day prednisone or equivalent (may be given in two daily doses-especially for patients that have nocturnal diarrhea).** Assess for dehydration. Patients may require hospitalization for IV steroids (1-2 mg/kg/day methylprednisolone).

** Once patients have improvement of symptoms to grade 0 or 1 taper of steroids should occur over at least 1 month- if patients have been started on budesonide in addition to systemic steroids, start tapering the prednisone **FIRST**.

**Do NOT administer loperamide in patients with ≥ Grade 2 diarrhea as this**

**may cause toxic megacolon and/or perforation.**

**If at any time patients experience diarrhea with the following symptoms: fever or abdominal pain patients should have a CT scan of the abdomen to rule out perforation. Emergent surgical evaluation should be performed if perforation is found. If a patient has bloody diarrhea, a Gastroenterology consult should be obtained. A Gastroenterology consult should be obtained if provider is considering infliximab for treatment of colitis.**

**For all patients- assess hydration status and monitor electrolytes, including** **magnesium.**

- 1. Rash

9.621 Grade 1 or 2- start oral non-sedating daily antihistamine (i.e Zyrtec® (cetirizine) or Claritin® (loratadine)) and use topical hydrocortisone cream to areas of rash with pruritis. If rash continues to progress, start systemic steroids at 0.5 mg/kg daily.

9.622 Grade 3 or greater. Initiate systemic steroid therapy at 1-2 mg/kg/day of prednisone or equivalent.

- 1. Hyperthyroidism

9.631 Grade 3- Initiate systemic steroid treatment at 1-2 mg/kg/day of prednisone or equivalent.

9.632 Grade 4 - Initiate systemic steroid therapy at 1-2 mg/kg/day of prednisone or equivalent.

- 1. Immune-Mediated Hypophysitis

9.641 Grade 2 and 3 – Initiate systemic steroid at 0.5-1mg/kg/day of prednisone or equivalent and physiologic replacement dose.

9.642 Grade 4 – Initiate systemic steroid at 1-2 mg/kg/day of prednisone or equivalent and physiologic replacement dose.

- 1. Pneumonitis

9.651 Grade 1- No change in treatment

9.652 Grade 2- Initiate steroids at 0.5-1mg/kg/day of prednisone or equivalent. 9.653 Grade 3 or greater- Consider hospitalization, and initiate systemic

steroids

- 1. Liver Dysfunction (AST or ALT)

9.661 AST or ALT ≤3 ULN- No change in treatment

9.662 AST or ALT >3 but <5 ULN or Grade 2- Consider initiating systemic steroids at 0.5-1mg/kg/day of prednisone or equivalent.

9.663 AST or ALT ≥Grade 3- Discontinue pembrolizumab. Initiate systemic steroid therapy.

- 1. Liver Dysfunction (Bilirubin)

9.671 Grade 1- No change in treatment

9.672 Grade 2- Consider initiating systemic steroids at 0.5-1mg/kg/day of prednisone or equivalent.

9.673 Grade 3 or greater- Monitor levels until resolved to ≤Grade 1. Initiate therapy with steroids at 1-2 mg/kg/day of prednisone or equivalent.

- 1. Side effect management for side effects attributable to ibrutinib

9.71 Fever

9.711 Grade 1 and 2- Manage symptomatically with antipyretics (acetaminophen or ibuprofen). Hold ibrutinib until fever <Grade 1.

9.712 Grade 3- Hold Ibrutinib until fever <Grade1. Manage symptomatically with antipyretics as above. Add prednisone at 10 mg daily. Rule out infectious etiology. If Grade 3 >24 hours proceed to management for Grade 4 pyrexia.

9.713 Grade 4- Discontinue Ibrutinib. Hospitalize patient until fever <Grade 2.

Rule out infectious etiology and start prednisone at 10-20 mg daily.

# Adverse Event (AE) Monitoring and Reporting

The site principal investigator is responsible for reporting any/all serious adverse events to the sponsor as described within the protocol, regardless of attribution to study agent or treatment procedure.

The sponsor/sponsor-investigator is responsible for notifying FDA and all participating investigators in a written safety report of any of the following:

- - - Any suspected adverse reaction that is both serious and unexpected.
    - Any findings from laboratory animal or *in vitro* testing that suggest a significant risk for human subjects, including reports of mutagenicity, teratogenicity, or carcinogenicity.
    - Any findings from epidemiological studies, pooled analysis of multiple studies, or clinical studies, whether or not conducted under an IND and whether or not conducted by the sponsor, that suggest a significant risk in humans exposed to the drug
    - Any clinically important increase in the rate of a serious suspected adverse reaction over the rate stated in the protocol or Investigator’s Brochure (IB).

Summary of SAE Reporting for this study (please read entire section for specific instructions):

| WHO: | WHAT form: | WHERE to send: |
| --- | --- | --- |
| All sites | Pregnancy Reporting [http://ctep.cancer.gov/protocolDevelopment/electronic_a](http://ctep.cancer.gov/protocolDevelopment/electronic_applications/docs/PregnancyReportFormUpdated.pdf)  [pplications/docs/PregnancyReportFormUpdated.pdf](http://ctep.cancer.gov/protocolDevelopment/electronic_applications/docs/PregnancyReportFormUpdated.pdf) | Mayo Sites – attach to MCCC Electronic SAE Reporting Form  Non Mayo sites – complete and  forward to [CancerCROSafetyIN@Mayo.edu](mailto:CancerCROSafetyIN@Mayo.edu) |
| Mayo Clinic Sites | Mayo Clinic Cancer Center SAE Reporting Form: [http://livecycle2.mayo.edu/workspace/?startEndpoint=](http://livecycle2.mayo.edu/workspace/?startEndpoint=MC4158-56/Processes/MC4158-56-Process.MC4158-56) [MC4158-56/Processes/MC4158-56-Process.MC4158-](http://livecycle2.mayo.edu/workspace/?startEndpoint=MC4158-56/Processes/MC4158-56-Process.MC4158-56)  [56](http://livecycle2.mayo.edu/workspace/?startEndpoint=MC4158-56/Processes/MC4158-56-Process.MC4158-56)  AND attach MedWatch 3500A:  [http://www.fda.gov/downloads/AboutFDA/ReportsMa](http://www.fda.gov/downloads/AboutFDA/ReportsManualsForms/Forms/UCM048334.pdf) [nualsForms/Forms/UCM048334.pdf](http://www.fda.gov/downloads/AboutFDA/ReportsManualsForms/Forms/UCM048334.pdf) | Will automatically be sent to [CANCERCROSAFETYIN@mayo](mailto:CANCERCROSAFETYIN@mayo.edu)  [.edu](mailto:CANCERCROSAFETYIN@mayo.edu) |

Definitions

*Adverse Event*

Any untoward medical occurrence associated with the use of a drug in humans, whether or not considered drug related.

*Suspected Adverse Reaction*

Any adverse event for which there is a reasonable possibility that the drug caused the adverse event.

*Expedited Reporting*

Events reported to sponsor within 24 hours, 5 days or 10 days of study team becoming aware of the event.

*Routine Reporting*

Events reported to sponsor via case report forms

*Events of Interest*

Events that would not typically be considered to meet the criteria for expedited reporting, but that for a specific protocol are being reported via expedited means in order to facilitate the review of safety data (may be requested by the FDA or the sponsor).

*Unanticipated Adverse Device Event (UADE)*

Any serious adverse effect on health or safety or any life-threatening problem or death caused by, or associated with, a device, if that effect, problem, or death was not previously identified in nature, severity, or degree of incidence in the investigational plan or application (including a supplementary plan or application), or any other unanticipated serious problem associated with a device that relates to the rights, safety, or welfare of subjects

- 1. Adverse Event Characteristics

**CTCAE term (AE description) and grade:** The descriptions and grading scales found in the revised NCI Common Terminology Criteria for Adverse Events (CTCAE) version

4.0 will be utilized for AE reporting. All appropriate treatment areas should have access to a copy of the CTCAE version 4.0. A copy of the CTCAE version 4.0 can be downloaded from the CTEP web site: (<http://ctep.cancer.gov/protocolDevelopment/electronic_applications/ctc.htm>)

1. Identify the grade and severity of the event using the CTCAE version 4.0.
2. Determine whether the event is expected or unexpected (see Section 10.2).
3. Determine if the adverse event is related to the study intervention (agent, treatment or procedure) (see Section 10.3).
4. Determine whether the event must be reported as an expedited report. If yes, determine the timeframe/mechanism (see Section 10.4).
5. Determine if other reporting is required (see Section 10.5).
6. Note: All AEs reported via expedited mechanisms must also be reported via the routine data reporting mechanisms defined by the protocol (see Sections 10.6 and 18.0).

NOTE: A severe AE is NOT the same as a serious AE, which is defined in Section 10.4.

- 1. Expected vs. Unexpected Events

*Expected events* - are those described within the Section 15.0 of the protocol, the study specific consent form, package insert (if applicable), and/or the investigator brochure, (if an investigator brochure is not required, otherwise described in the general investigational plan).

*Unexpected adverse events* or suspected adverse reactions are those not listed in Section

15.0 of the protocol, the study specific consent form, package insert (if applicable), or in the investigator brochure (or are not listed at the specificity or severity that has been observed); if an investigator brochure is not required or available, is not consistent with the risk information described in the general investigational plan.

*Unexpected* also refers to adverse events or suspected adverse reactions that are mentioned in the investigator brochure as occurring with a class of drugs but have not been observed with the drug under investigation.

An investigational agent/intervention might exacerbate the expected AEs associated with a commercial agent. Therefore, if an expected AE (for the commercial agent) occurs with a higher degree of severity or specificity, expedited reporting is required.

NOTE: *The consent form may contain study specific information at the discretion of the Principal Investigator; it is possible that this information may NOT be included in the protocol or the investigator brochure. Refer to protocol or IB for reporting needs.

- 1. Attribution to agent(s) or procedure

When assessing whether an adverse event (AE) is related to a medical agent(s) medical or procedure, the following attribution categories are utilized:

Definite - The AE *is clearly related* to the agent(s)/procedure. Probable - The AE *is likely related* to the agent(s)/procedure. Possible - The AE *may be related* to the agent(s)/procedure. Unlikely - The AE *is doubtfully related* to the agent(s)/procedure. Unrelated - The AE *is clearly NOT related* to the agent(s)/procedure.

- 1. **AEs Experienced Utilizing Investigational Agents and Commercial Agent(s) on the SAME (Combination) Arm**

**NOTE:** When a commercial agent(s) is (are) used on the same treatment arm as the investigational agent/intervention (also, investigational drug, biologic, cellular product, or other investigational therapy under an IND), the **entire combination (arm) is then considered an investigational intervention for reporting.**

- An AE that occurs on a combination study must be assessed in accordance with the guidelines for **investigational** agents/interventions.
- An AE that occurs prior to administration of the investigational agent/intervention must be assessed as specified in the protocol. In general, only Grade 4 and 5 AEs that are unexpected with at least possible attribution to the commercial agent require an expedited report, unless hospitalization is required. Refer to Section 10.4 for specific AE reporting requirements or exceptions.

An investigational agent/intervention might exacerbate the expected AEs associated with a commercial agent. Therefore, if an expected AE (for the commercial agent) occurs with a higher degree of severity or specificity, expedited reporting is required.

- An increased incidence of an expected adverse event (AE) is based on the patients treated for this study at their site. A list of known/expected AEs is reported in the package insert or the literature, including AEs resulting from a drug overdose.
- Commercial agent expedited reports must be submitted to the FDA via MedWatch 3500A for Health Professionals (complete all three pages of the form).

[http://www.fda.gov/downloads/AboutFDA/ReportsManualsForms/Forms/UCM0](http://www.fda.gov/downloads/AboutFDA/ReportsManualsForms/Forms/UCM048334.pdf) [48334.pdf](http://www.fda.gov/downloads/AboutFDA/ReportsManualsForms/Forms/UCM048334.pdf)

or [http://www.fda.gov/AboutFDA/ReportsManualsForms/Forms/ListFormsAlphabe](http://www.fda.gov/AboutFDA/ReportsManualsForms/Forms/ListFormsAlphabetically/default.htm)

[tically/default.htm](http://www.fda.gov/AboutFDA/ReportsManualsForms/Forms/ListFormsAlphabetically/default.htm)

Instructions for completing the MedWatch 3500A: [http://www.fda.gov/downloads/Safety/MedWatch/HowToReport/DownloadFor](http://www.fda.gov/downloads/Safety/MedWatch/HowToReport/DownloadForms/UCM387002.pdf) [ms/UCM387002.pdf](http://www.fda.gov/downloads/Safety/MedWatch/HowToReport/DownloadForms/UCM387002.pdf)

- 1. **EXPECTED Serious Adverse Events: Protocol Specific Exceptions to Expedited Reporting**

For this protocol only, the following Adverse Events/Grades are expected to occur within this population and do not require Expedited Reporting. These events must still be reported via Routine Reporting (see Section 10.6).*

*Report any clinically important increase in the rate of a serious suspected adverse reaction (at your study site) over that which is listed in the protocol or investigator brochure as an expedited event.

*Report an expected event that is greater in severity or specificity than expected as an expedited event.

| **System Organ Class**  **(SOC)** | **Adverse event/ Symptoms** | **CTCAE Grade at which the event**  **will not be expeditedly reported^1^** |
| --- | --- | --- |
| Blood and lymphatic system disorders | Anemia | ≤Grade 3 |
|  | Febrile neutropenia | ≤Grade 3 |
| Cardiac Disorders | Myocardial infarction | ≤Grade 3 |
| Endocrine disorders | Hypothyroidism | ≤Grade 3 |
|  | Hyperthyroidism | ≤Grade 3 |
|  | Hypophysitis | ≤Grade 3 |
| Gastrointestinal disorders | Nausea | ≤Grade 3 |
|  | Vomiting | ≤Grade 3 |
|  | Gastric hemorrhage | ≤Grade 3 |
|  | Abdominal pain | ≤Grade 3 |
|  | Diarrhea | ≤Grade 3 |
|  | Hepatitis | ≤Grade 3 |
| General disorders and  administrations site conditions | Fatigue | ≤Grade 3 |
|  | Fever | ≤Grade 3 |
| Immune system disorders | Allergic reaction/anaphylaxis | ≤Grade 3 |
| Injury, poisoning, and procedural complications | Wound dehiscence | ≤Grade 3 |
| Investigations | Neutrophil count decreased | ≤Grade 4 |
|  | Platelet count decreased | ≤Grade 3 |
|  | White blood cell decreased | ≤Grade 4 |
|  | AST | ≤Grade 3 |
|  | Alkaline Phosphatase | ≤Grade 3 |
|  | Total Bilirubin | ≤Grade 3 |
| Musculoskeletal and connective tissue disorders | Arthralgia | ≤Grade 3 |
|  | Myalgia | ≤Grade 3 |
| Respiratory disorders | Pneumonitis | ≤Grade 3 |
| Nervous system disorders | Intracranial hemorrhage | ≤Grade 3 |
|  | Leukoencephalopathy | ≤Grade 3 |

| **System Organ Class (SOC)** | **Adverse event/ Symptoms** | **CTCAE Grade at which the event will not be expeditedly reported^1^** |
| --- | --- | --- |
|  | Peripheral sensory neuropathy | ≤Grade 3 |
| Renal and urinary disorders | Proteinuria | ≤Grade 4 |
| Respiratory, thoracic and mediastinal disorders | Bronchopulmonary hemorrhage | ≤Grade 3 |
| Skin and subcutaneous  tissue disorders | Rash maculo-papular | ≤Grade 4 |
| Vascular disorders | Hypertension | ≤Grade 3 |
|  | Thromboembolic event | ≤Grade 3 |

^1^ These exceptions only apply if the adverse event does not result in hospitalization. If the adverse event results in hospitalization, then the standard expedited adverse events reporting requirements must be followed.

Specific protocol exceptions to expedited reporting should be reported expeditiously by investigators **ONLY** if they exceed the expected grade of the event.

The following hospitalizations are not considered to be SAEs because there is no “adverse event” (*i.e.*, there is no untoward medical occurrence) associated with the hospitalization:

- Hospitalizations for respite care
- Planned hospitalizations required by the protocol
- Hospitalization planned before informed consent (where the condition requiring the

hospitalization has not changed post study drug administration)

- Hospitalization for elective procedures unrelated to the current disease and/or

treatment on this trial

- Hospitalization for administration of study drug or insertion of access for

administration of study drug

- Hospitalization for routine maintenance of a device (*e.g.*, battery replacement) that was in place before study entry
- Hospitalization, or other serious outcomes for signs and symptoms of progression of

the cancer.

- 1. Expedited Reporting Requirements for IND/IDE Agents
  2. **Phase 1 and Early Phase 2 Studies: Expedited Reporting Requirements for Adverse Events that Occur on Studies under an IND/IDE within 30 Days of the Last Administration of the Investigational Agent/Intervention** ^1,^ ^2^

| **FDA REPORTING REQUIREMENTS FOR SERIOUS ADVERSE EVENTS (21 CFR Part 312)**  **NOTE:** Investigators **MUST** immediately report to the sponsor **ANY** Serious Adverse Events, whether or not they are considered related to the investigational agent(s)/intervention (21 CFR 312.64)  An adverse event is considered serious if it results in **ANY** of the following outcomes:   1. Death 2. A life-threatening adverse event 3. An adverse event that results in inpatient hospitalization or prolongation of existing hospitalization for ≥ 24   hours   1. A persistent or significant incapacity or substantial disruption of the ability to conduct normal life functions 2. A congenital anomaly/birth defect. 3. Important Medical Events (IME) that may not result in death, be life threatening, or require hospitalization may be considered serious when, based upon medical judgment, they may jeopardize the patient or subject and may require medical or surgical intervention to prevent one of the outcomes listed in this definition. (FDA, 21 CFR 312.32; ICH E2A and ICH E6). | | |
| --- | --- | --- |
| **ALL SERIOUS** adverse events that meet the above criteria MUST be immediately reported to the sponsor within the timeframes detailed in the table below. | | |
| **Hospitalization** | **Grade 1 and Grade 2 Timeframes** | **Grade 3-5 Timeframes** |
| Resulting in Hospitalization  ≥24 hrs | 7 Calendar Days | 24-Hour 3 Calendar Days |
| Not resulting in Hospitalization  ≥24 hrs | Not required |  |
| **Expedited AE reporting timelines are defined as:**   - “24-Hour; 3 Calendar Days” - The AE must initially be reported within 24 hours of learning of the AE, followed by a complete expedited report within 3 calendar days of the initial 24-hour report. - “7 Calendar Days” - A complete expedited report on the AE must be submitted within 7 calendar days of learning of the AE. | | |
| 1Serious adverse events that occur more than 30 days after the last administration of investigational agent/intervention and have an attribution of possible, probable, or definite require reporting as follows: **Expedited 24-hour notification followed by complete report within 3 calendar days for:**   - All Grade 3, 4, and Grade 5 AEs   **Expedited 7 calendar day reports for:**   - Grade 2 AEs resulting in hospitalization or prolongation of hospitalization   2 For studies using PET or SPECT IND agents, the AE reporting period is limited to 10 radioactive half-lives, rounded UP to the nearest whole day, after the agent/intervention was last administered. Footnote “1” above applies after this reporting period.  Effective Date: May 5, 2011 | | |

NOTE: Refer to Section 10.32 for exceptions to Expedited Reporting

**Adverse Events of Special Interest (AESI)**

Specific adverse events, or groups of adverse events, will be followed as part of standard safety monitoring activities by the Sponsor. These events (regardless of seriousness) should be reported to on the Serious Adverse Event Report Form and sent via email or fax to Pharmacyclics Drug Safety, or designee, within 15 days of awareness.

**Major Hemorrhage**

Major hemorrhage is defined as any of the following:

- Any treatment-emergent hemorrhagic adverse events of Grade 3 or higher*. Any treatment-emergent serious adverse events of bleeding of any grade
- Any treatment-emergent central nervous system hemorrhage/hematoma of any grade

*All hemorrhagic events requiring transfusion of red blood cells should be reported as Grade 3 or higher AE per CTCAE vX.X.

Events meeting the definition of major hemorrhage will be captured as an event of special interest according to Section 11.4.6 above.

- 1. General reporting instructions

The Mayo IND Coordinator will assist the sponsor-investigator in the processing of expedited adverse events and forwarding of suspected unexpected serious adverse reactions (SUSARs) to the FDA and IRB.

Use Mayo Expedited Event Report form [http://livecycle2.mayo.edu/workspace/?startEndpoint=MC4158-](http://livecycle2.mayo.edu/workspace/?startEndpoint=MC4158-56/Processes/MC4158-56-Process.MC4158-56) [56/Processes/MC4158-56-Process.MC4158-56](http://livecycle2.mayo.edu/workspace/?startEndpoint=MC4158-56/Processes/MC4158-56-Process.MC4158-56) for investigational agents or commercial/investigational agents on the same arm.

All serious adverse events and AESIs (initial and follow-up information) will be reported on FDA Medwatch (Form 3500A) or Suspect Adverse Event Report (CIOMS Form 1) IRB Reporting Form) and sent via email ([AEintakeCT@pcyc.com](mailto:AEintakeCT@pcyc.com)) or fax ((408) 215-3500) to Pharmacyclics Drug Safety, or designee, within 15 days of the event. Pharmacyclics may request follow-up and other additional information from the Sponsor Investigator.

For commercial agents:

Submit form MedWatch 3500A to the FDA, 5600 Fishers Lane, Rockville, MD 20852-9787, by fax at 1-800-332-0178 or online at [http://www.fda.gov/Safety/MedWatch/HowToReport/default.htm.](http://www.fda.gov/Safety/MedWatch/HowToReport/default.htm)

**Mayo Clinic Cancer Center (MCCC) Institutions**:

Submit copies, along with the Event Reporting coversheet using the Mayo Clinic Cancer Center Expedited Event Report form [http://livecycle2.mayo.edu/workspace/?startEndpoint=MC4158-](http://livecycle2.mayo.edu/workspace/?startEndpoint=MC4158-56/Processes/MC4158-56-Process.MC4158-56) [56/Processes/MC4158-56-Process.MC4158-56,](http://livecycle2.mayo.edu/workspace/?startEndpoint=MC4158-56/Processes/MC4158-56-Process.MC4158-56) which will send a copy to the following email address: [CANCERCROSAFETYIN@mayo.edu.](mailto:CANCERCROSAFETYIN@mayo.edu) This email will be managed by the SAE, IND and Safety Reporting Coordinators.

- 1. Reporting of re-occurring SAEs

ALL SERIOUS adverse events that meet the criteria outlined in table10.41 MUST be immediately reported to the sponsor within the timeframes detailed in the corresponding table. This reporting includes, but is not limited to SAEs that re-occur again after resolution.

- 1. Other Required Reporting
  2. Unanticipated Problems Involving Risks to Subjects or Others (UPIRTSOS)

Unanticipated Problems Involving Risks to Subjects or Others (UPIRTSOS) in general, include any incident, experience, or outcome that meets **all** of the following criteria:

1. Unexpected (in terms of nature, severity, or frequency) given (a) the research procedures that are described in the protocol-related documents, such as the IRB-approved research protocol and informed consent document; and (b) the characteristics of the subject population being studied;
2. Related or possibly related to participation in the research (in this guidance document, possibly related means there is a reasonable possibility that the incident, experience, or outcome may have been caused by the procedures involved in the research); and
3. Suggests that the research places subjects or others at a greater risk of harm (including physical, psychological, economic, or social harm) than was previously known or recognized.

Some unanticipated problems involve social or economic harm instead of the physical or psychological harm associated with adverse events. In other cases, unanticipated problems place subjects or others at increased *risk* of harm, but no harm occurs.

Note: If there is no language in the protocol indicating that pregnancy is not considered an adverse experience for this trial, and if the consent form does not indicate that subjects should not get pregnant/impregnate others, then any pregnancy in a subject/patient or a male patient’s partner (spontaneously reported) which occurs during the study or within 120 days of completing the study should be reported as a UPIRTSO.

**Mayo Clinic Cancer Center (MCCC) Institutions:**

If the event meets the criteria for IRB submission as a Reportable Event/UPIRTSO, provide the Reportable Event coversheet and appropriate documentation to [CANCERCROSAFETYIN@mayo.edu.](mailto:CANCERCROSAFETYIN@mayo.edu) The Mayo Regulatory Affairs Office will review and process the submission to the Mayo Clinic IRB.

- 1. Death

**Note: A death on study requires both routine and expedited reporting regardless of causality, unless as noted below. Attribution to treatment or other cause must be provided.**

Any death occurring within 30 days of the last dose, regardless of attribution to an agent/intervention under an IND/IDE requires expedited reporting within 24-hours.

Any death occurring greater than 30 days with an attribution of possible, probable, or definite to an agent/intervention under an IND/IDE requires expedited reporting within 24-hours.

**Reportable categories of Death**

- - - Death attributable to a CTCAE term.
    - Death Neonatal: A disorder characterized by cessation of life during the first 28 days of life.
    - Death NOS: A cessation of life that cannot be attributed to a CTCAE term associated with Grade 5.
    - Sudden death NOS: A sudden (defined as instant or within one hour of the onset of symptoms) or an unobserved cessation of life that cannot be attributed to a CTCAE term associated with Grade 5.
    - Death due to progressive disease should be reported as **Grade 5 “Neoplasms benign, malignant and unspecified (including cysts and polyps) – Other (Progressive Disease)”** under the system organ class (SOC) of the same name. Evidence that the death was a manifestation of underlying disease (e.g., radiological changes suggesting tumor growth or progression: clinical deterioration associated with a disease process) should be submitted.
  1. Secondary Malignancy
     - A ***secondary malignancy*** is a cancer caused by treatment for a previous malignancy (e.g., treatment with investigational agent/intervention, radiation or chemotherapy). A secondary malignancy is not considered a metastasis of the initial neoplasm.
     - All secondary malignancies that occur following treatment with an agent under an IND/IDE will be reported. Three options are available to describe the event:
       - Leukemia secondary to oncology chemotherapy (e.g., Acute Myeloctyic Leukemia [AML])
       - Myelodysplastic syndrome (MDS)
       - Treatment-related secondary malignancy
     - Any malignancy possibly related to cancer treatment (including AML/MDS) should also be reported via the routine reporting mechanisms outlined in each protocol.
  2. Second Malignancy

A second malignancy is one unrelated to the treatment of a prior malignancy (and is NOT a metastasis from the initial malignancy). Second malignancies require ONLY routine reporting unless otherwise specified.

- 1. Other Malignancies

All new malignant tumors including solid tumors, skin malignancies and hematologic malignancies will be reported for the duration of study treatment and during any protocol-specified follow-up periods including post-progression follow-up for overall survival. If observed, enter data in the corresponding eCRF.

- 1. Pregnancy, Fetal Death, and Death Neonatal

Before study enrollment, subjects must agree to take appropriate measures to avoid pregnancy. However, should a pregnancy occur in a female study subject, consent to provide follow-up information regarding the outcome of the pregnancy and the health of the infant until 30 days old will be requested.

A female subject or female partner of a male subject must immediately inform the investigator if she becomes pregnant from the time of consent to 90 days after the last dose of study drug(s). Any female subjects receiving study drug(s) who become pregnant must immediately discontinue study drug. The investigator should counsel the subject, discussing any risks of continuing the pregnancy and any possible effects on the fetus.

Although pregnancy itself is not regarded as an AE, the outcome will need to be documented. Any pregnancy occurring in a female subject or female partner of a male subject must be reported from the time of first dose up until 90 days after the last dose of study drug(s). Any occurrence of pregnancy must be recorded on the Pregnancy Report Form Part I and sent via email or fax to Pharmacyclics Drug Safety, or designee, per SAE reporting timelinesof learning of the event.

All pregnancies will be followed for outcome, which is defined as elective termination of the pregnancy, miscarriage, or delivery of the fetus. For pregnancies with an outcome of live birth, the newborn infant will be followed until 30 days old by completing the Pregnancy Report Form Part II. Any congenital anomaly/birth defect noted in the infant must be reported as a SAE.

If a female subject (or female partner of a male subject) taking investigational product becomes pregnant, the subject taking should notify the Investigator, and the pregnant female should be advised to call her healthcare provider immediately. The patient should have appropriate follow-up as deemed necessary by her physician. If the baby is born with a birth defect or anomaly, a second expedited report is required.

Prior to obtaining private information about a pregnant woman and her infant, the investigator must obtain consent from the pregnant woman and the newborn infant’s parent or legal guardian before any data collection can occur. A consent form will need to be submitted to the IRB for these subjects if a pregnancy occurs. If informed consent is not obtained, no information may be collected.

In cases of fetal death, miscarriage or abortion, the mother is the patient. In cases where the child/fetus experiences a serious adverse event other than fetal death, the child/fetus is the patient.

NOTE: When submitting Mayo Expedited Adverse Event Report reports for “Pregnancy”, “Pregnancy loss”, or “Neonatal loss”, the potential risk of exposure of the fetus to the investigational agent(s) or chemotherapy agent(s) should be documented in the “Description of Event” section. Include any available medical documentation. Include this form:

[http://ctep.cancer.gov/protocolDevelopment/electronic_applications/docs/Pregna](http://ctep.cancer.gov/protocolDevelopment/electronic_applications/docs/PregnancyReportFormUpdated.pdf) [ncyReportFormUpdated.pdf](http://ctep.cancer.gov/protocolDevelopment/electronic_applications/docs/PregnancyReportFormUpdated.pdf)

10.561 Pregnancy

Pregnancy should be reported in an expedited manner as

**Grade 3 “Pregnancy, puerperium and perinatal conditions**

**- Other (pregnancy)”** under the Pregnancy, puerperium and perinatal conditions SOC. Pregnancy should be followed until the outcome is known.

10.562 Fetal Death

Fetal death is defined in CTCAE as “A disorder characterized by death in utero; failure of the product of conception to show evidence of respiration, heartbeat, or definite movement of a voluntary muscle after expulsion from the uterus, without possibility of resuscitation.”

Any fetal death should be reported expeditiously, as **Grade 4 “Pregnancy, puerperium and perinatal conditions - Other (pregnancy loss)”** under the Pregnancy, puerperium and perinatal conditions SOC.

10.563 Death Neonatal

Neonatal death, defined in CTCAE as “A disorder characterized by cessation of life occurring during the first 28 days of life” that is felt by the investigator to be at least possibly due to the investigational agent/intervention, should be reported expeditiously.

A neonatal death should be reported expeditiously as **Grade 4 “General disorders and administration - Other (neonatal loss)”** under the General disorders and administration SOC.

- 1. Required Routine Reporting
  2. Baseline and Adverse Events Evaluations

Pretreatment symptoms/conditions to be graded at baseline and adverse events to be graded at each evaluation.

Grading is per CTCAE v4.0 **unless** alternate grading is indicated in the table below:

| System Organ Class (SOC) | Adverse event/Symptoms | Baseline | Each evaluation |
| --- | --- | --- | --- |
| Blood and lymphatic system disorders | Anemia | X | X |
| Gastrointestinal disorders | Baseline # stools | X |  |
|  | Diarrhea |  | X |
| General disorders | Fatigue | X | X |
|  | Fever |  | X |
| Immune system disorders | Allergic reaction |  | X |
|  | Anaphylaxis |  | X |
| Investigations | Blood bilirubin increased |  | X |
| Respiratory system disorders | Dyspnea | X | X |

| System Organ Class (SOC) | Adverse event/Symptoms | Baseline | Each evaluation |
| --- | --- | --- | --- |
| Skin and subcutaneous tissue disorders | Rash, maculo-papular | X | X |

- 1. Other AEs

Submit via appropriate MCCC Case Report Forms (i.e., paper or electronic, as applicable) the following AEs experienced by a patient and not specified in Section 10.6:

10.621 Grade 2 AEs deemed *possibly, probably, or definitely*

related to the study treatment or procedure.

10.622 Grade 3 and 4 AEs regardless of attribution to the study treatment or procedure.

10.623 Grade 5 AEs (Deaths)

10.6231 Any death within 30 days of the patient’s last study treatment or procedure regardless of attribution to the study treatment or procedure.

10.6232 Any death more than 30 days after the patient’s last study treatment or procedure that is felt to be at least possibly treatment related must also be submitted as a Grade 5 AE, with a CTCAE type and attribution assigned.

- 1. Late Occurring Adverse Events

Refer to the instructions in the Forms Packet (or electronic data entry screens, as applicable) regarding the submission of late occurring AEs following completion of the Active Monitoring Phase (i.e., compliance with Test Schedule in Section 4.0)

- 1. Pharmacyclics Additional Event Reporting Instructions Definitions

Adverse Events

An AE is any untoward medical occurrence in a subject administered a pharmaceutical product and which does not necessarily have a causal relationship with this treatment. An AE can therefore be any unfavorable and unintended sign (including a clinically significant abnormal laboratory finding, for example), symptom, or disease temporally associated with the use of an investigational study drug, whether or not considered related to the study drug (ICH-E2A 1995).

For the purposes of this clinical study, AEs include events which are either new or represent detectable exacerbations of pre-existing conditions.

The term “disease progression” should not be reported as an AE term. As an example, "worsening of underlying disease" or the clinical diagnosis that is associated with disease progression should be reported.

Adverse events may include, but are not limited to:

- - - Subjective or objective symptoms provided by the subject and/or observed by the investigator or study staff including laboratory abnormalities of clinical significance.
    - Any AEs experienced by the subject through the completion of final study procedures.
    - AEs not previously observed in the subject that emerge during the protocol- specified AE reporting period, including signs or symptoms associated with the underlying disease that were not present before the AE reporting period
    - Complications that occur as a result of protocol-mandated interventions (eg, invasive procedures such as biopsies).

The following are NOT considered AEs:

- - - **Pre-existing condition:** A pre-existing condition (documented on the medical history CRF) is not considered an AE unless the severity, frequency, or character of the event worsens during the study period.
    - **Pre-planned or elective hospitalization:** A hospitalization planned before signing the ICF is not considered an SAE, but rather a therapeutic intervention. However, if during the pre-planned hospitalization an event occurs, which prolongs the hospitalization or meets any other SAE criteria, the event will be considered an SAE. Surgeries or interventions that were under consideration, but not performed before enrollment in the study, will not be considered serious if they are performed after enrollment in the study for a condition that has not changed from its baseline level. Elective hospitalizations for social reasons, solely for the administration of chemotherapy, or due to long travel distances are also not SAEs.
    - **Diagnostic Testing and Procedures:** Testing and procedures should not to be reported as AEs or SAEs, but rather the cause for the test or procedure should be reported.

A serious adverse event (SAE) based on International Conference on Harmonisation (ICH) and EU Guidelines on Pharmacovigilance for Medicinal Products for Human Use is any untoward medical occurrence that at any dose:

- - - Results in death (ie, the AE actually causes or leads to death).
    - Is life-threatening. Life-threatening is defined as an AE in which the subject was at risk of death at the time of the event. It does not refer to an event which hypothetically might have caused death if it were more severe. If either the investigator or the Sponsor believes that an AE meets the definition of life- threatening, it will be considered life-threatening.
    - Requires in-patient hospitalization >24 hours or prolongation of existing hospitalization.
    - Results in persistent or significant disability/incapacity (ie, the AE results in substantial disruption of the subject’s ability to conduct normal life functions).
    - Is a congenital anomaly/birth defect.
    - Is an important medical event that may not result in death, be immediately

life-threatening or require hospitalization, but may be considered an SAE when,

based upon appropriate medical judgment, the event may jeopardize the subject or subject may require intervention to prevent one of the other outcomes listed in this definition. Examples of such events are intensive treatment in an emergency department or at home for allergic bronchospasm, blood dyscrasias, or convulsion that does not result in hospitalization; or development of drug dependency or drug abuse.

All serious adverse events and AESIs (initial and follow-up information) will be reported on FDA Medwatch (Form 3500A) or Suspect Adverse Event Report (CIOMS Form 1) IRB Reporting Form and sent via email ([AEintakeCT@pcyc.com](mailto:AEintakeCT@pcyc.com)) or fax ((408) 215- 3500) to Pharmacyclics Drug Safety, or designee , within 24 hours of the event.

Pharmacyclics may request follow-up and other additional information from the Sponsor Investigator.

All SAEs that have not resolved by the end of the study, or that have not resolved upon discontinuation of the subject's participation in the study, must be followed until any of the following occurs:

- The event resolves
- The event stabilizes
- The event returns to baseline, if a baseline value/status is available
- The event can be attributed to agents other than the study drug or to factors unrelated to study conduct.
- It becomes unlikely that any additional information can be obtained (subject or health care practitioner refusal to provide additional information, lost to follow up after demonstration of due diligence with follow-up efforts)

The Sponsor assumes responsibility for appropriate reporting of AEs to the regulatory authorities and governing bodies according to the local regulations.

The investigator (or Sponsor where required) must report these events to the appropriate Independent Ethics Committee/Institutional Review Board (IEC/IRB) that approved the protocol unless otherwise required and documented by the IEC/IRB.

# Treatment Evaluation Using RECIST Guideline

- 1. Requirements

The first scan after treatment initiation will be performed at the completion of Cycle 4 and will be then performed at the complete of every other cycle of treatment until disease progression is confirmed or 12 cycles of treatment have been administered without a confirmed disease progression (that is, imaging is to be done at the end of Cycles 4, 6, 8, 10, and 12). If a patient has not had disease progression after Cycle 12, subsequent scans will take place at the end of every 3^rd^ cycle of treatment thereafter (that is, at the end of Cycle 15, 18, 21 until confirmed disease progression)

Disease progression for this protocol is defined as meeting the RECIST criteria for disease progression on two consecutive evaluations at least 6 weeks apart.

- 1. Definitions of Measurable and Non-Measurable Disease
  2. Measurable Disease
  3. A non-nodal lesion is considered measurable if its longest diameter can be accurately measured as ≥1.0 cm with CT scan, CT component of a PET/CT, or MRI.
  4. A malignant lymph node is considered measurable if its short axis is

>1.5 cm when assessed by CT scan (CT scan slice thickness recommended to be no greater than 5 mm).

**NOTE:** *Tumor lesions in a previously irradiated area are not considered measurable disease.*

- 1. Non-Measurable Disease

All other lesions (or sites of disease) are considered non-measurable disease, including pathological nodes (those with a short axis ≥1.0 to <1.5 cm). Bone lesions, leptomeningeal disease, ascites, pleural/pericardial effusions, lymphangitis cutis/pulmonis, inflammatory breast disease, and abdominal masses (not followed by CT or MRI), are considered as non-measurable as well.

**NOTE:** ‘Cystic lesions’ thought to represent cystic metastases can be considered as measurable lesions, if they meet the definition of measurability described above. However, if non-cystic lesions are present in the same patient, these are preferred for selection as target lesions.

Lymph nodes that have a short axis <1.0 cm are considered non- pathological (i.e., normal) and should not be recorded or followed.

- 1. Guidelines for Evaluation of Measurable Disease
  2. Measurement Methods:
- All measurements should be recorded in metric notation (i.e., decimal fractions of centimeters) using a ruler or calipers.
- The same method of assessment and the same technique must be used to characterize each identified and reported lesion at baseline and during follow-up. For patients having only lesions measuring at least 1 cm to less than 2 cm must use CT imaging for both pre- and post-treatment tumor assessments.
  1. Acceptable Modalities for Measurable Disease

Conventional CT and MRI: This guideline has defined measurability of lesions on CT scan based on the assumption that CT slice thickness is 5 mm or less. If CT scans have slice thickness greater than 5 mm, the minimum size for a measurable lesion should be twice the slice thickness

- - - As with CT, if an MRI is performed, the technical specifications of the scanning sequences used should be optimized for the evaluation of the type and site of disease. The lesions should be measured on the same pulse sequence. Ideally, the same type of scanner should be used and the image acquisition protocol should be followed as closely as possible to prior scans. Body scans should be performed with breath-hold scanning techniques, if possible.
- PET-CT: If the site can document that the CT performed as part of a PET-CT is of identical diagnostic quality to a diagnostic CT (with IV and oral contrast), then the CT portion of the PET-CT can be used for RECIST measurements and can be used interchangeably with conventional CT in accurately measuring cancer lesions over time.
- FDG-PET: FDG-PET scanning is allowed to complement CT scanning in assessment of progressive disease [PD] and particularly possible 'new' disease. A ‘positive’ FDG-PET scanned lesion is defined as one which is FDG avid with an update greater than twice that of the surrounding tissue on the attenuation corrected image; otherwise, an FDG-PET scanned lesion is considered ‘negative.’ New lesions on the basis of FDG-PET imaging can be identified according to the following algorithm:
  1. Negative FDG-PET at baseline with a positive FDG-PET at follow-up is a sign of PD based on a new lesion.
  2. No FDG-PET at baseline and a positive FDG-PET at follow-up:
     1. If the positive FDG-PET at follow-up corresponds to a new site of disease confirmed by CT, this is PD.
     2. If the positive FDG-PET at follow-up is not confirmed as a new site of disease on CT at the same evaluation, additional follow-up CT scans (i.e., additional follow-up scans at least 4 weeks later) are needed to determine if there is truly progression occurring at that site. In this situation, the date of PD will be the date of the initial abnormal PDG-PET scan.

iii If the positive FDG-PET at follow-up corresponds to a pre-existing site of disease on CT that is not progressing on the basis of the anatomic images, it is not classified as PD.

- 1. Measurement at Follow-up Evaluation:
     - A subsequent scan must be obtained 6 weeks following initial documentation of an objective status of either complete response (CR) or partial response (PR).
     - In the case of stable disease (SD), follow-up measurements must have met the SD criteria at least once after study entry at a minimum interval of

6 weeks until Cycle 12, thereafter every 12 weeks (see Section 11.44).

- - - The cytological confirmation of the neoplastic origin of any effusion that appears or worsens during treatment when the measurable tumor has met criteria for response or stable disease is mandatory to differentiate between response or stable disease (an effusion may be a side effect of the treatment) and progressive disease.
    - Cytologic and histologic techniques can be used to differentiate between PR and CR in rare cases (e.g., residual lesions in tumor types such as germ cell tumors, where known residual benign tumors can remain.)
  1. Measurement of Effect
  2. Target Lesions & Target Lymph Nodes
- Measurable lesions (as defined in Section 11.21) up to a maximum of 5 lesions, representative of all involved organs, should be identified as “Target Lesions” and recorded and measured at baseline. These lesions can be non- nodal or nodal (as defined in 11.21), where no more than 2 lesions are from the same organ and no more than 2 malignant nodal lesions are selected.

**Note:** If fewer than 5 target lesions and target lymph nodes are identified (as there often will be), there is no reason to perform additional studies beyond those specified in the protocol to discover new lesions.

- Target lesions and target lymph nodes should be selected on the basis of their size, be representative of all involved sites of disease, but in addition should be those that lend themselves to reproducible repeated measurements. It may be the case that, on occasion, the largest lesion (or malignant lymph node) does not lend itself to reproducible measurements in which circumstance the next largest lesion (or malignant lymph node) which can be measured reproducibly should be selected.
- Baseline Sum of Dimensions (BSD): A sum of the longest diameter for all target lesions plus the sum of the short axis of all the target lymph nodes will be calculated and reported as the baseline sum of dimensions (BSD). The BSD will be used as reference to further characterize any objective tumor response in the measurable dimension of the disease.
- Post-Baseline Sum of the Dimensions (PBSD): A sum of the longest diameter for all target lesions plus the sum of the short axis of all the target lymph nodes will be calculated and reported as the post-baseline sum of dimensions (PBSD). If the radiologist is able to provide an actual measure for the target lesion (or target lymph node), that should be recorded, even if it is below 0.5 cm. If the target lesion (or target lymph node) is believed to be present and is faintly seen but too small to measure, a default value of 0.5 cm should be assigned. If it is the opinion of the radiologist that the target lesion or target lymph node has likely disappeared, the measurement should be recorded as 0 cm.
- The minimum sum of the dimensions (MSD) is the minimum of the BSD and the PBSD.
  1. Non-Target Lesions & Non-Target Lymph Nodes

Non-measurable sites of disease (Section 11.22) are classified as non- target lesions or non-target lymph nodes and should also be recorded at base line. These lesions and lymph nodes should be followed in accord with 11.433.

- 1. Response Criteria

11.431 All target lesions and target lymph nodes followed by CT/MRI/PET-CT must be measured on re-evaluation at evaluation times specified in Section 11.1. Specifically, a change in objective status to either a PR or CR cannot be done without re-measuring target lesions and target lymph nodes.

**Note:** Non-target lesions and non-target lymph nodes should be evaluated at each assessment, especially in the case of first response or confirmation of response. In selected circumstances, certain non-target organs may be evaluated less frequently. For example, bone scans may need to be repeated only when complete response is identified in target disease or when progression in bone is suspected.

11.432 Evaluation of Target Lesions

Complete Response (CR): All of the following must be true:

1. Disappearance of all target lesions.
2. Each target lymph node must have reduction in short axis to <1.0 cm.

Partial Response (PR): At least a 30% decrease in PBSD (sum of

the longest diameter for all target lesions plus the sum of the short axis of all the target lymph nodes at current evaluation) taking as reference the BSD (*see* Section 11.41).

Progression (PD): At least one of the following must be

true:

1. At least one new malignant lesion, which also includes any lymph node that was normal at baseline (<1.0 cm short axis) and increased to ≥1.0 cm short axis during follow-up.
2. At least a 20% increase in PBSD (sum of the longest diameter for all target lesions plus the sum of the short axis of all the target lymph nodes at current evaluation) taking as reference the MSD (Section 11.41). In addition, the PBSD must also demonstrate an absolute increase of at least 0.5 cm from the MSD.
3. See Section 11.32 for details in regards to the requirements for PD via FDG-PET imaging.

Stable Disease (SD): Neither sufficient shrinkage to qualify for

PR, nor sufficient increase to qualify for PD taking as reference the MSD.

11.433 Evaluation of Non-Target Lesions & Non-target Lymph Nodes Complete Response (CR): All of the following must be true:

1. Disappearance of all non-target

lesions.

1. Each non-target lymph node must have a reduction in short axis to

<1.0 cm.

Non-CR/Non-PD: Persistence of one or more non-target

lesions or non-target lymph nodes.

Progression (PD): At least one of the following must be

true:

1. At least one new malignant lesion, which also includes any lymph node that was normal at baseline

(<1.0 cm short axis) and increased to ≥1.0 cm short axis during follow- up.

1. Unequivocal progression of existing non-target lesions and non-target lymph nodes. (NOTE: Unequivocal progression should not normally trump target lesion and target lymph node status. It must be representative of overall disease status change.)
2. See Section 11.32 for details in regards to the requirements for PD via FDG-PET imaging.
   1. Overall Objective Status

The overall objective status for an evaluation is determined by combining the patient’s status on target lesions, target lymph nodes, non-target lesions, non- target lymph nodes, and new disease as defined in the following table:

| **Target Lesions & Target Lymph Nodes** | **Non-Target Lesions & Non-Target Lymph Nodes** | **New**  **Sites of Disease** | **Overall Objective Status** |
| --- | --- | --- | --- |
| CR | CR | No | CR |
| CR | Non-CR/Non-PD | No | PR |
| PR | CR  Non-CR/Non-PD | No | PR |
| CR/PR | Not All Evaluated* | No | PR |
| SD | CR  Non-CR/Non-PD Not All Evaluated* | No | SD |

| **Target Lesions & Target Lymph Nodes** | **Non-Target Lesions & Non-Target Lymph Nodes** | **New**  **Sites of Disease** | **Overall Objective Status** |
| --- | --- | --- | --- |
| Not all Evaluated | CR  Non-CR/Non-PD Not All Evaluated* | No | Not Evaluated (NE) |
| PD | Unequivocal PD CR  Non-CR/Non-PD  Not All Evaluated* | Yes or No | PD |
| CR/PR/SD/PD/Not all Evaluated | Unequivocal PD | Yes or No | PD |
| CR/PR/SD/PD/Not all Evaluated | CR  Non-CR/Non-PD Not All Evaluated* | Yes | PD |

*See Section 11.431

- 1. Symptomatic Deterioration

Patients with global deterioration of health status requiring discontinuation of treatment without objective evidence of disease progression at that time, and not either related to study treatment or other medical conditions, should be reported as PD due to “symptomatic deterioration.” Every effort should be made to document the objective progression even after discontinuation of treatment due to symptomatic deterioration. A patient is classified as having PD due to “symptomatic deterioration” if any of the following occur that are not either related to study treatment or other medical conditions:

- - - Weight loss >10% of body weight.
    - Worsening of tumor-related symptoms.
    - Decline in performance status of >1 level on ECOG scale.

# Descriptive Factors

- 1. Dose level as assigned by Registration Office: -1 vs. dose level 0 vs. dose level 1 vs. expansion

# Treatment/Follow–up Decision at Evaluation of Patient

- 1. Continuation of therapy

Patients who have not had disease progression and have experienced acceptable toxicity are to continue treatment per protocol until confirmed PD, unacceptable toxicity or refusal. And then they will go to event monitoring where patient and disease status will be reported every 6 months until death or a maximum of 5 years post-registration.

- 1. Progressive disease

The first instance in which a patient’s disease status meets the RECIST criteria for PD will continue two additional cycles of treatment per protocol and then undergo disease revaluation. If the initial documented progression is not confirmed by this scan, patients will continue treatment per protocol. If the initial documented progression is confirmed by this scan, patients will go to the event-monitoring phase where patient and disease status will be reported every 6 months until death or a maximum of 5 years post- registration. ***Exception: Patients who develop PD in the CNS should discontinue study treatment and go to Event Monitoring.***

- 1. For those on treatment at least one year and in complete response (CR)

At investigator’s discretion patients who have completed at least one year of protocol therapy and have had a complete response (CR) may discontinue all protocol therapy when the duration of the complete response has been at least 6 months (that is, at least 3 consecutive scans showing CR).

- 1. Discontinuation of protocol therapy

Criteria for discontinuation of protocol therapy include:

- - - Disease progression: Where disease progression is: the development of a new metastatic lesion or an objective status of disease progression (as defined by RECIST criteria) on two consecutive evaluations at least 6 weeks apart (section 13.2). ***Exception: Patients who develop PD in the CNS should discontinue study treatment.***
    - Request by patient to discontinue study treatment
    - Unacceptable toxicity
    - Intercurrent illness that would, in the judgment of the investigator, affect assessments of clinical status to a significant degree or require discontinuation of drug
    - Administration of radiotherapy, non-protocol chemotherapy, immunotherapy, biological agents, or an experimental drug during the trial
    - Development of new primary cancer
    - Ineligibility

Patients who discontinue treatment due to progression or development of a second primary, desire for non-protocol treatment, intolerability, patient request, physician decision, or inter-current illness preventing further administration of protocol treatment will proceed to event monitoring phase of the trial where patient and disease status until death or a maximum of 5 years post-registration

- 1. Ineligible

A patient is deemed ineligible if after registration, it is determined that at the time of registration, the patient did not satisfy each and every eligibility criteria for study entry.

- - - If the patient received any protocol treatment, patient should discontinue study treatment and all data (except biospecimens) up until the point of discontinuation must be submitted. Patients will proceed to event monitoring phase of the trial where patient and disease status will be reported every 6 months until death or a maximum of 5 years post-registration.
    - If the patient never received any protocol treatment, on-study material (except biospecimens) and the End of Active Treatment/Cancel Notification Form must be submitted. No further No further data submission is necessary.
  1. Cancel

A patient who withdraws consent before any study treatment is given. On-study material (except biospecimens) and the End of Active Treatment/Cancel Notification Form must be submitted. No further data submission is necessary.

- 1. Treatment after PD

Subsequent treatment after confirmed PD is at the discretion of their attending physician.

- 1. PD in CNS

Patients who develop PD in the CNS only should discontinue study treatment and proceed to event monitoring phase of the trial where patient and disease status will be reported every 6 months until death or a maximum of 5 years post-registration.

MC1577 50 MCCC Amendment 8

# Body Fluid Biospecimens

- 1. Summary Table of Research Blood and Body Fluid Specimens to be Collected for this Protocol

| **Correlative Study**  **(Section 14.2 for more information)** | **Mandatory or Optional** | **Blood or Body Fluid**  **being Collected** | **Type of Collection**  **Tube (color of tube top)** | **Volume to collect per tube (# of**  **tubes to be collected)** | **After consenting to pre- registration**  **phase** | **Cycle 1,**  **Day 8 (±1day)** | **All other cycles*, Day 1**  **(±3 days)** | **At**  **confirmed progression** | **Process at site?**  **(Yes or No)** | **Temperature Conditions for Storage**  **/Shipping** |
| --- | --- | --- | --- | --- | --- | --- | --- | --- | --- | --- |
| Th1/Th2 polarity assays, immunophenotyping assays, plasma cytokine assays, and T cell and myeloid- derived suppressor cells (MDSCs) functional assays | Mandatory | Plasma, Peripheral blood mononuclear cells (PBMCs),  and whole blood | Sodium Heparin (Green) | 10 mL(7) | X | X | X | X | Yes | Ambient for initial transport, frozen liquid nitrogen for storage |
|  | Mandatory | Plasma, Peripheral blood mononuclear cells (PBMCs),  and whole blood | EDTA  (Pink) | 6mL (1) | X | X | X | X | Yes | Ambient for initial transport, frozen liquid nitrogen for storage |

^*^C2-C4, then at the beginning of each cycle when tumor imaging is performed. Please see table in Section 4.0, research blood specimen.

- 1. Collection and Processing

Peripheral blood will be collected in green top Vacutainer tubes. A special study refer card will be generated to identify these specimens. The samples will be kept at room temperature until processing. Blood samples will be collected at the clinical phlebotomy areas in the Mayo Clinic.

Seventy-six mL of collected blood will be sent in an expedited fashion to Dr. Markovic’s laboratory c/o Wendy K Nevala. Samples will be processed within 12 hours of receipt.

Blood samples will be layered onto Ficoll and centrifuged to isolate plasma and peripheral blood mononuclear cells (PBMCs). Isolated plasma and cells will be frozen at

-80 °C and in liquid nitrogen, respectively, for later use.

The six mL EDTA collected whole blood will be sent in an expedited fashion to Dr. Michael Gustafson’s lab. Blood samples will be immediately used for designed correlative studies in Human Cellular Therapy Lab.

| Samples to be collected | Peripheral whole blood (70 mL) |
| --- | --- |
| Tubes for collection | Green top Vacutainer tubes |
| Location for collection | Clinical phlebotomy areas in the Mayo Clinic |
| Delivery for processing | At room temperature |
|  | Dr. Markovic’s lab, GU-323 |
|  | c/o Wendy Nevala |
| Nature of processing | Ficoll centrifugation within 12 hours of receipt |
| Storage after processing | Liquid Nitrogen (PBMCs), -80 °C (plasma) |

| Samples to be collected | Peripheral whole blood (6 mL) |
| --- | --- |
| Tubes for collection | Pink top Vacutainer tubes |
| Location for collection | Clinical phlebotomy areas in the Mayo Clinic |
| Delivery for processing | At room temperature |
|  | Human Cellular Therapy Lab (Hilton 2) |
|  | c/o Michael Deeds |
| Nature of processing | Immediate processing per research experiment |
| Storage after processing | N/A |

- 1. Background and Methodology
  2. Th1/Th2 polarity assays

Frozen PBMCs from pre-treatment, post-cycle 1, and post-cycle 2 will be thawed and cultured for four hours with paramagnetic beads (Dynal, Oslo, Norway) coated with anti-CD3 and anti-CD28 antibodies (R and D Systems Minneapolis, MN). Following stimulation, cultured cells will be assessed via flow cytometry by measuring intracellular interferon gamma and interleukin (IL)-4. Brefeldin A will be purchased from Sigma-Aldrich (St. Louis, MO). Fluorochrome-labeled antibodies to IFNγ, IL-4, CD8, CD4, CD14, CD16, and CD19 will be purchased from Becton Dickinson (San Jose, CA). The stained samples will be analyzed by flow cytometry (FACScan and CellQuest software, Becton-Dickinson).

- 1. Immunophenotyping assays

The systemic impact of ibrutinib and pembrolizumab on immune cell subsets will be ascertained by immunophenotypic analysis of frozen PBMC for subsets of T helper cells, macrophages, and DC. To do this, we will label PBMC with antibodies to CD3, CD4, CD11c, CD14, C16, CD19, CD123, CD197, CD206,

CD294, HLA-DR, (BD Biosciences, San Jose, CA) and TIM-3 (R&D Systems, Inc., Minneapolis, MN). Immunophenotyping will be performed using manufacturer’s instruction in batch samples of the same patients analyzed on the same day. The stained samples will be analyzed by flow cytometry (FACScan and CellQuest software (BD Biosciences, San Jose, CA). Samples will be

assessed for the ratio of Th1 to Th2 cells (CD4+TIM-3+/CD4+CD294+), DC1 to DC2 cells (CD3-CD14-CD16-CD19-HLADR+CD11c+ to CD3-CD14-CD16- CD19-HLADR+CD123-), and M1 to M2 cells (CD14+CD197+ to

CD14+CD206+). Changes in Th1/Th2 ratio, DC1/DC2 ratio, and M1/M2 ratio between the pre-treatment sample and each other sample will be used to determine the systemic impact of treatment on immune cell subsets.

- 1. Plasma cytokine assay

We will profile the plasma cytokine changes as a result of vaccine administration. The BioRad human 27-plex cytokine panel will be used (Cat # 171-A11127, Bio-Rad, San Diego CA) for the measurements of plasma concentrations of IL-1β, IL-1rα, IL-2, IL-4, IL-5, IL-6, IL-7, IL-8, IL-9, IL-10, IL-12(p70), IL-13, IL-15, IL-17, basic FGF, eotaxin, G-CSF, GM-CSF, IFN-γ,

IP-10, MCP-1, MIP-1α, MIP-1β, PDGF, RANTES, TNF-α, and VEGF. The

assay will be performed as per the manufacturer’s directions. Briefly, 100 mcL of Bio-Plex assay buffer will be added to each well of a MultiScreen MABVN 1.2 um microfiltration plate followed by the addition of 50 mcL of the multiplex bead preparation. Following washing of the beads with the addition of 100 mcL of wash buffer, 50 mcL of the samples or the standards will be added to each well and incubated with shaking for 30 minutes at room temperature. The plasma (1:3 dilution) and standards will be diluted using the Bio-Plex human serum diluent kit and plated in duplicate. Standard curves will be generated with a mixture of 27 cytokine standards and eight serial dilutions ranging from 0-32,000 pg/mL. The plate will then be washed 3 times followed by incubation of each well in 25 mcL of pre-mixed detection antibodies for 30 minutes with shaking.

The plate will further be washed and 50 mcL of streptavidin solution were added to each well and incubated for 10 minutes at room temperature with shaking. The beads will be given a final washing and resuspension in 125 UL of Bio-Plex assay buffer. Cytokine levels in the sera will be quantified by analyzing 100 mcL of each well on a Bio-Plex using Bio-Plex Manager software version 4.0. Normal values for plasma cytokine concentrations were generated by analyzing 30 plasma samples from healthy donors (blood donors at the Mayo Clinic Dept. of Transfusion Medicine). A set of five normal plasma samples (standards) will be run alongside all batches of plasma analysis in this study. If the cytokine concentrations of the “standard” samples differ by more than 20%, results will be rejected and the plasma samples re-analyzed.

- 1. Tetramer assays

We will compare pre- and post-treatment frequencies of melanoma-specific CD8 T cells via tetramer staining in HLA-A2-positive patients. PBMCs will be labeled with HLA-A2 tetramers complexed with melanoma-associated antigen peptides (MART-127-35, GP100209-217, survivin-- ELTLGEFLKL, and tyrosinase368-376), and control peptides. PBMCs will be counterstained with anti-CD8 (BD Biosciences, San Jose, CA). The stained samples will be analyzed by flow cytometry (FACScan and CellQuest software, Becton-Dickinson).

- 1. T cell functional assays

One biopsy core from a pre-treatment metastatic lesion will be assessed for core volume (length of core x 3.14 x radius^2^) and percent tumor cells. The core will then undergo freeze thaw to make a tumor cell lysate. PBMCs will be sorted using CD14 antibody-coated beads. CD14+ cells will be cultured with GM-CSF and IL-4 for 5 days to make immature DCs (iDCs). The iDCs will be cultured with tumor lysates or media (negative control) and maturation factors to make loaded and unloaded mature DCs (mDCs). The loaded and unloaded mDCs will be incubated with CD14- cells. ELIspot assays for IFNg will be performed for CD4 and CD8 T cells.

- 1. Peripheral blood T cell Bim levels

PBMCs will be labeled with antibodies to CD8, CD11a, PD-1, and intracellular BIM. BIM levels will be quantitated in tumor-related (CD8+CD11a+PD-1+) cells. BIM levels will be compared in pre-versus post-treatment cells.

- 1. Characterization of tumor-infiltrating leukocytes

Tumor biopsy cores will be fixed in formalin and embedded in paraffin, then sectioned for immunohistochemistry (IHC). Sections will be stained for CD4, CD8, FoxP3, CD20, PD-1, and PD-L1. The number of cells bearing each marker will be quantitated.

# Drug Information

- 1. Ibrutinib (IMBRUVICA^®^, PCI-32765)
  2. **Background**

Ibrutinib is an antieoplastic agent that is an inhibitor of Bruton’s tyrosine kinase.

- 1. **Formulation**

Capsules: 140 mg capsules. The capsules also contain the following compendial excipients: microcrystalline cellulose, croscarmellose sodium, sodium lauryl sulfate, and magnesium stearate. Capsules are packaged in high-density polyethylene bottles with an induction seal and a child resistant screw-top cap.

- 1. **Preparation, storage, and stability**

Ibrutinib capsules: 140mg store at 15°C to 25°C (59°F to 77°F) with excursions permitted to 30°C (86°F). The compound is not light-sensitive.

- 1. **Dose and Administration**

Ibrutinib X mg (X x 140-mg capsules) is administered orally once daily. The capsules are to be taken around the same time each day with 8 ounces (approximately 240 mL) of water. The capsules should be swallowed intact and subjects should not attempt to open capsules or dissolve them in water. The use of strong CYP3A inhibitors/inducers, and grapefruit and Seville oranges should be avoided for the duration of the study (Appendix V).

If a dose is not taken at the scheduled time, it can be taken as soon as possible on the same day with a return to the normal schedule the following day. The subject should not take extra capsules to make up the missed dose.

The first dose will be delivered in the clinic on Day 1, after which subsequent dosing is typically on an outpatient basis. Ibrutinib will be dispensed to subjects in bottles at each visit. Study drug may not be shipped to the subject without approval from PCYC and may not be dispensed to anyone other than the subject. Unused ibrutinib dispensed during previous visits must be returned to the site and drug accountability records updated at each visit. Returned capsules must not be redispensed to anyone

- 1. **Pharmacokinetic information**

Following oral administration of ibrutinib at doses ranging from 420 to 840 mg/day, exposure to ibrutinib increased proportionally with substantial

intersubject variability. The mean terminal plasma elimination half life (t1/2) of ibrutinib ranged from 4 to 6 hours, with a median time to maximum plasma concentration (Tmax) of 1 to 2 hours. Despite the doubling in mean systemic exposure when dosed with food, the favorable safety profile of ibrutinib allows dosing with or without food. Ibrutinib is extensively metabolized primarily by CYP 3A-mediated metabolic pathways. The on-target effects of metabolite PCI- 45227 are not considered clinically relevant. Steady-state exposure of ibrutinib and PCI-45227 was less than 2-fold of first dose exposure implying

non-clinically relevant accumulation. About 8% of ibrutinib is excreted in the urine. Ibrutinib exposure is not altered in patients with creatinine clearance (CrCl) > 30 mL/min. Patients with severe renal impairment or patients on dialysis have not been studied. Following single dose administration, the AUC of ibrutinib increased 2.7-, 8.2- and 9.8-fold in subjects with mild (Child-Pugh class

A), moderate (Child-Pugh class B), and severe (Child-Pugh class C) hepatic impairment compared to subjects with normal liver function. A higher proportion of Grade 3 or higher adverse reactions were reported in patients with B-cell malignancies (CLL, MCL and WM) with mild hepatic impairment based on NCI organ dysfunction working group (NCI-ODWG) criteria for hepatic dysfunction compared to patients with normal hepatic function.

For the most up to date and comprehensive pharmacokinetics (PK) and product metabolism information regarding ibrutinib, please refer to the current IB.

- 1. **Potential Drug Interaction**

Ibrutinib is primarily metabolized by cytochrome P450 enzyme 3A4/5. Avoid concomitant use of ibrutinib with any of the following: CYP3A4 inducers (strong), CYP3A4 inhibitors (strong or moderate), and herbs that are CYP3A4 inducers. (See Appendix IV)

Grapefruit juice and Seville oranges moderately inhibit 3A4 and may increase ibrutinib exposure.

- 1. **Known potential adverse events**
     - A list of expected adverse reactions is provided in Appendix VI.
     - Please refer to the latest version of Investigator Brochure for a summary of clinical safety data.

**Bleeding-related Events**

There have been reports of hemorrhagic events in subjects treated with ibrutinib, both with and without thrombocytopenia. These include minor hemorrhagic events such as contusion, epistaxis, and petechiae; and major hemorrhagic events, some fatal, including gastrointestinal bleeding, intracranial hemorrhage, and hematuria. In an in vitro platelet function study, inhibitory effects of ibrutinib on collagen-induced platelet aggregation were observed, refer to Section 8.34.

Use of ibrutinib in subjects requiring other anticoagulants or medications that inhibit platelet function may increase the risk of bleeding. See Section 8.34 for guidance on concomitant use of anticoagulants, antiplatelet therapy and/or supplements. See Section 8.34 for guidance on ibrutinib management with surgeries or procedures. Patients with congenital bleeding diathesis have not been studied.

**Infections**

Infections (including sepsis, bacterial, viral, or fungal infections) were observed in subjects treated with ibrutinib therapy. Some of these infections have been associated with hospitalization and death. Consider prophylaxis according to standard of care in subjects who are at increased risk for opportunistic. Although causality has not been established, cases of progressive multifocal leukoencephalopathy (PML) and hepatitis B reactivation have occurred in subjects treated with ibrutinib. Subjects should be monitored for signs and symptoms (fever, chills, weakness, confusion, vomiting and jaundice) and appropriate therapy should be instituted as indicated.

**Cytopenias**

Treatment-emergent Grade 3 or 4 cytopenias (neutropenia, thrombocytopenia, and anemia) were reported in subjects treated with ibrutinib. Subjects should be monitored for fever, weakness, or easy bruising and/or bleeding. Monitor complete blood counts monthly.

**Interstitial Lung Disease (ILD)**

Cases of interstitial lung disease (ILD) have been reported in subjects treated with ibrutinib. Monitor subjects for pulmonary symptoms indicative of ILD. Should symptoms develop follow the protocol dose modification guidelines (see Section 8.1).

**Cardiac Arrhythmias**

Atrial fibrillation, atrial flutter, and cases of ventricular tachyarrhythmia including some fatal events, have been reported in subjects treated with ibrutinib, particularly in subjects with cardiac risk factors, hypertension, acute infections, and a previous history of cardiac arrhythmia. Subjects who develop arrhythmic symptoms (eg, palpitations, lightheadedness, syncope, chest discomfort or new onset of dyspnea) should be evaluated clinically, and if indicated, have an ECG performed. For cardiac arrhythmia which persist, consider the risks and benefits of ibrutinib treatment and follow the protocol dose modification guidelines (see Section 8.1).

**Tumor Lysis Syndrome**

Tumor lysis syndrome has been reported with ibrutinib therapy. Subjects at risk of tumor lysis syndrome are those with high tumor burden prior to treatment.

Monitor subjects closely and take appropriate precautions.

**Non-melanoma Skin Cancer**

Non-melanoma skin cancers have occurred in subjects treated with ibrutinib. Monitor subjects for the appearance of non-melanoma skin cancer.

**Diarrhea**

Diarrhea is the most frequently reported non-hematologic AE with ibrutinib monotherapy and combination therapy. Other frequently reported gastrointestinal events include nausea, vomiting, and constipation. These events are rarely severe severe and are generally managed with supportive therapies including antidiarrheals and antiemetics. Subjects should be monitored carefully for gastrointestinal AEs and cautioned to maintain fluid intake to avoid dehydration. Medical evaluation should be made to rule out other etiologies such as Clostridium difficile or other infectious agents. Should symptoms be severe or prolonged follow the protocol dose modification guidelines (see Section 8.1).

**Rash**

Rash has been commonly reported in subjects treated with either single agent ibrutinib or in combination with chemotherapy. Most rashes were mild to moderate in severity. Isolated cases of severe cutaneous adverse reactions (SCARs) including Stevens-Johnson syndrome (SJS) have been reported in subjects treated with ibrutinib. Subjects should be closely monitored for signs and symptoms suggestive of SCAR including SJS. Subjects receiving ibrutinib should be observed closely for rashes and treated symptomatically, including interruption of the suspected agent as appropriate. In addition, hypersensitivity- related events including erythema, urticaria, and angioedema have been reported.

**Hypertension**

Hypertension has been commonly reported in subjects treated with ibrutinib. Monitor subjects for new onset of hypertension or hypertension that is not adequately controlled after starting ibrutinib. Adjust existing anti-hypertensive medications and/or initiate anti-hypertensive treatment as appropriate.

- 1. **Drug procurement**

Drug will be provided free of charge to study participants by Pharmacyclics, Inc.

- 1. **Nursing guidelines**
- There are numerous drug to drug interactions. Record all of patient’s medications including OTC, and herbal use. Avoid concomitant use with agents as listed in Section 8.2, and Appendix IV.
- Ibrutinib should be taken with water at approximately the same time each day and can be taken with or without food. Capsules should be swallowed whole.
- Patients should be instructed to avoid eating grapefruit (including juice) and Seville oranges while on ibrutinib.
- Peripheral edema is common. Instruct patients to report this to the study team.
- Gastrointestinal side effects are common (diarrhea, nausea, constipation, abdominal pain, vomiting, etc). Treat symptomatically and monitor for effectiveness of intervention.
- Monitor CBC w/diff. Instruct patients in energy conserving lifestyle (anemia) and to report any unusual bruising or bleeding and/or signs or symptoms of infection to study team.
- Arthralgias, Myalgias, and muscle spasm can be seen. Treat symptomatically and monitor for effectiveness.
- Monitor renal function/uric acid levels, especially in patients who may be experiencing dehydration.
- Respiratory symptoms may include, cough, SOB, and URI. Instruct patients to report these symptoms to the study team.
- Rarely patients can experience secondary skin cancers. Instruct patients to report any new skin lesions to the study team.
- Rash can be seen. Instruct patient to report to study team.
  1. Pembrolizumab (KEYTRUDA^®^)
  2. Background

Pembrolizumab is a highly selective anti-PD-1 humanized monoclonal antibody which inhibits programmed cell death-1 (PD-1) activity by binding to the PD-1 receptor on T-cells to block PD-1 ligands (PD-L1 and PD-L2) from binding.

Blocking the PD-1 pathway inhibits the negative immune regulation caused by PD-1 receptor signaling (Hamid, 2013). Anti-PD-1 antibodies (including pembrolizumab) reverse T-cell suppression and induce antitumor responses

- 1. Formulation
     - Solution, Intravenous [preservative free]: 100 mg/4 mL (4 mL)
     - Lyophilized powder in single –use vial for reconstitution, 50 mg
  2. Preparation, storage, and stability:

Pembrolizumab will be obtained from commercial supply.

- 1. Administration:

IV: Infuse over 30 minutes through a 0.2 to 5 micron sterile, nonpyrogenic, low- protein binding inline or add-on filter. Do not infuse other medications through the same infusion line.

- 1. Pharmacokinetic information

Half-lift elimination: Half-life elimination: 26 days

- 1. Potential Drug Interaction:

There are no known significant interactions.

- 1. Known potential adverse events:

Consult the package insert for the most current and complete information.

**Common known potential toxicities, >10%:**

Cardiovascular: Peripheral edema

Central nervous system: Fatigue, headache, chills, insomnia, dizziness Dermatologic: Pruritus, skin rash, vitiligo

Endocrine & metabolic: Hyperglycemia, hyponatremia, hypoalbuminemia, hypertriglyceridemia, hypocalcemia

Gastrointestinal: Nausea, decreased appetite, constipation, diarrhea, vomiting, abdominal pain

Hematologic & oncologic: Anemia Hepatic: Increased serum AST

Neuromuscular & skeletal: Arthralgia, limb pain, myalgia, back pain Respiratory: Cough, dyspnea, upper respiratory tract infection Miscellaneous: Fever

**Less common known potential toxicities, 1% - 10%**: Dermatologic: Cellulitis

Endocrine & metabolic: Hypothyroidism, hyperthyroidism Gastrointestinal: Colitis

Infection: Sepsis Renal: Renal failure

Respiratory: Pneumonitis, pneumonia

**Rare known potential toxicities, <1% (Limited to important or life- threatening):** Adrenocortical insufficiency (immune-mediated), arthritis (immune-mediated), exfoliative dermatitis (immune-mediated), hemolytic anemia (immune-mediated), hepatitis (including autoimmune hepatitis; grade 4), hypophysitis, interstitial nephritis, Lambert-Eaton syndrome (immune-mediated), myositis (immune-mediated), nephritis (grade 2 autoimmune), optic neuritis (immune-mediated), pancreatitis (immune-mediated), partial epilepsy (immune- mediated; in a patient with inflammatory foci in brain parenchyma), rhabdomyolysis (immune-mediated), uveitis (immune-mediated)

- 1. Drug Procurement

Commercial supplies. Pharmacies or clinics shall obtain supplies from normal commercial supply chain or wholesaler.

- 1. Nursing guidelines
  2. Pembrolizumab side effects vary greatly from those of traditional chemotherapy and can vary in severity from mild to life threatening. Instruct patients to report any side effects to the study team immediately. Side effects may be immediate or delayed up to months after discontinuation of therapy. Most side effects are reversible with prompt intervention of corticosteroids.
  3. Diarrhea can be seen however is less common than that seen with anti- CTLA-4 agents. However it can be severe, leading to colonic perforation. Instruct patients to report ANY increase in the number of stools and/or change in baseline, blood in the stool, abdominal pain to the study team immediately.
  4. Rash/pruirits/dermatitis is seen. Patients should report any rash to the study team. Treat per Section 9.0 and monitor for effectiveness.
  5. Monitor LFTs closely as elevations in these levels could indicate early onset autoimmune hepatitis. Patients should also be instructed to report any jaundice, or right upper quadrant pain to the study team immediately.
  6. Pneumonitis can be seen and may be mild (only seen on imaging) to severe. Patients should be instructed to report any SOB, dyspnea, cough, chest pain, etc. to the study team immediately. Patients reporting these symptoms should have a pulse ox checked and consider immediate imaging per the treating MD.
  7. Endocrinopathies (including hypopituitarism, hypothyroidism, hypophysistis, and adrenal insufficiency) are seen with this agent. Patients may present only with the vague sense of fatigue and “not feeling well” . Additional symptoms may be that of nausea, sweating and decreased activity tolerance. Instruct patients to report these signs or symptoms immediately and obtain appropriate labs as ordered by MD.
  8. Patients who are started on steroid therapy for any side effects of pembrolizimab toxicity should be instructed to take the steroids as ordered, and not to discontinue abruptly as symptoms may return and be severe. Patients may be on steroid therapy for weeks. Instruct patients to report any increase or change in side effects with any dosage decrease as patients may need a slower taper.
  9. Fatigue is common and may or may not be associated with immune related side effects. Assess patient’s fatigue level prior to each cycle of therapy and report any changes to the study team.
  10. Patients should not receive live vaccines within 28 days before starting study drug or during study drug administration.

# Statistical Considerations and Methodology

- 1. Background

A 3+3 phase I clinical trial design will be used to determine the maximum tolerated dose of Ibrutinib when used in combination with Pembrolizumab in patients with metastatic melanoma who are Ipilimumab-naïve in the metastatic setting. Once determined, a dose expansion cohort will be used to determine an early signal of promising anti-tumor activity measured by tumor response.

- 1. Study Design – Phase I
  2. Definition of Maximum Tolerated Dose (MTD)

The maximum tolerated dose is defined as the highest dose level among those tested where at most one out of 6 patients develops a DLT prior to the start of their second cycle of treatment and the next highest dose level is such that 2 out of a maximum of 6 patients treated at this dose level developed a DLT prior to the start of their second cycle of treatment.

- 1. Enrollment and determination of MTD

16.221 A minimum of 2 or a maximum of 6 patients will be accrued to a given dose level.

For each dose level, patients will be accrued so that at any given time no more than 2 patients will be receiving their first cycle of treatment **and** acute adverse event data over the first treatment cycle for all other patients treated at the current dose level is known.

16.222 If, at any time in the enrollment process, 2 patients treated at the current dose level develop a DLT during the first cycle of treatment, enrollment will be closed to that dose level. Enrollment will be re-opened to the next lower dose level if fewer than 6 patients have been treated at that dose level in order to establish whether it is the MTD.

If none of the first 3 patients treated at a given dose level develops a DLT during the first cycle of treatment, enrollment to the dose level will be closed and enrollment will reopen at next higher dose level. If there are no other higher dose levels to be tested, three additional patients will enrolled at the current dose level to confirm MTD.

If one of the first 3 patients treated at a given dose level develops a DLT during the first cycle of treatment, three additional patients will enrolled (sequentially) onto the current dose level.

- If, at any time in the enrollment of these 3 additional patients, a patient develops a DLT, enrollment will be closed to this dose level. Enrollment will be re-opened to the next lower dose level if fewer than 6 patients have been treated at that dose level.
- If none of these 3 additional patients develops a DLT during the first cycle of treatment, enrollment to this dose level will be closed and enrollment will reopen at next higher dose level. If there are no other higher dose levels to be tested, this will be considered the MTD
  1. Study Design – Expansion Cohort (Treated at MTD)

Acknowledging both the short term aspect of the primary endpoint of the study and that 18 patients may be enrolled, we have chosen a phase II study design proposed by Gehan30. Two (2) responses in 14 eligible patients having initiate treatment will be considered indicative of promising activity and further study in a phase 2 study. According to Gehan, if a drug were 20 percent effective or more, there would be a 95% chance that one or more successes would be obtained in 14 patients^30^.

Gehan’s approach calculated cumulative probability of observing at most x successes in N patients (i.e., assuming a binomial distribution) assuming the true success rate is P.

Assuming true success (response) proportion in 14 patients is P=20%:

| If the number of responses (x) observed… | Then the probability of observing  ≤ x responses is less than or equal  to… |
| --- | --- |
| 0 | 0.044 |
| 1 | 0.198 |
| 2 | 0.448 |
| 3 | 0.698 |

Assuming true success (response) proportion in 14 patients is P=30%:

| If the number of responses observed… | Then the probability of observing  ≤ x responses is less than or equal  to… |
| --- | --- |
| 0 | 0.007 |
| 1 | 0.047 |
| 2 | 0.161 |
| 3 | 0.355 |

Two planned safety checks will occur after 3 and 6 patients have completed their first cycle of treatment. If we observe DLT(s) at either review of the data (1 DLT in the first 3 patients or 2 DLTs in the first 6 patients), we will drop the starting dose level to 280 mg and continue with the remainder of the study. Only one reduction in the starting dose level will be allowed. All data will be summarized by assigned starting dose level and by prior ICI therapy status.

- 1. Sample Size

Enrollment and anticipated number of patients, phase of study:

- - - Prior to MCCC Amendment #2, two patients were enrolled and the study was stopped due to toxicity. The study reopened was modified to a phase I (vs phase II).
    - Phase I - During the dose escalation phase, a minimum of 9 and a maximum of 18 patients is anticipated. Enrollment was completed prior to MCCC Amendment #6 (11 patients).
    - Phase I Dose Expansion: At the time of MCCC Amendment #6, 6 patients were enrolled at the MTD (Dose Level 1). Fourteen (14) patients are required for the dose expansion cohort component of this study. Assuming that these 6 patients

are included in the expansion cohort, an additional 8 patients are required for the design. Accounting for 2 non-evaluable patients (e.g, ineligible, cancellations), an additional 10 (i.e., 8 + 2) patients will be enrolled at the MTD to gain additional insight into the safety profile and clinical benefit of this treatment strategy (i.e., dose expansion cohort). That is, maximum enrollment is 16 patients.

Final enrollment is expected to be 2 (original phase II design) plus 5 treated at Dose Level 0 (phase I design), plus 16 [6 treated at dose level 2 of phase I design, plus the expansion cohort mentioned above (10)]. That is, final enrollment for the entire study is expected to be a maximum of 23 patients.

- 1. Accrual Duration:

We anticipate that approximately 10% of the patients who pre-register will fail to meet the criteria for registration or met the criteria for registration but refuse to continue.

2 patients will be enrolled per month during the dose expansion.

Given the nature of the accrual scheme, it will take 11-24 months to complete enrollment to the dose escalation phase (2 pts per month and a 1 month gap between each set of 2 pts) and then an additional 5-6 months to enroll the dose expansion cohort and associated with MCCC Amendment 6.

- 1. Definition of end points and analysis plan:

Patient evaluability: All patients meeting the eligibility criteria who have signed a consent form and have begun treatment will be included in the analysis of the safety and clinical outcome data.

The primary endpoints:

- - - Phase I/Dose Seeking: The endpoint is the maximum tolerated dose (MTD). The maximum tolerated dose (MTD) is defined as the highest dose level among those under consideration where at most 1 of 6 patients develops a dose limiting toxicity and 2 or more of the 3-6 patients treated at the next higher dose level develop a dose limiting toxicity.
    - Dose Expansion Cohort: The endpoint is tumor response. Patients will be classified with respect to their best tumor response during treatment (e.g., CR, PR, SD, confirmed PD, Death, Too Early, or Not Evaluable). Estimates of tumor response and binomial confidence intervals will be reported. A patient whose tumor has meet the RECIST criteria for CR or PR on two consecutive evaluations at least 8 weeks apart is considered to have had a tumor response.

Adverse events: The maximum grade of each type of toxicity will be recorded for each patient. For each toxicity reported by dose level, the percentage of patients developing any degree of that toxicity as well as the percentage of patients developing a severe degree (Grade 3 or higher) will be determined.

Secondary end points include tumor response (for the Phase I/Dose Seeking Cohort), progression-free survival (PFS) and overall survival (OS) time: A patient whose tumor has meet the RECIST criteria for CR or PR on two consecutive evaluations at least 8 weeks apart is considered to have had a tumor response. Survival time is defined as the time from study entry to death due to any cause. Progression-free survival time is the time from study entry to the documentation of disease progression. If a patient dies

without a recurrence documented, the patient will be considered to have progressed on their death date.

All the study observations pertaining to adverse events, MTD, response, time to event points and laboratory correlates will be examined in an exploratory and hypothesis- generating fashion. The small sample size and the heterogeneous patient population associated with Phase I studies restricts the generalizability of the results. Any notable statistical finding will be viewed as an impetus for further study in Phase II trials rather than a definitive finding in and of itself.

- 1. Monitoring of the trial

This clinical trial will be monitored by the Mayo Clinic Cancer Center Data and Safety Monitoring Board (MCCC DSMB). The study statistician will prepare a report containing accrual and adverse event that will be submitted to MCCC DSMB every 3 months.

16.71 Safety Stopping rules

Dose limiting toxicities and other acute adverse events will be monitored by the study team on a monthly basis. If during the course of enrolling the first 6 patients on trial, 2 patients develop a grade 3+ maculo-papular rash, pneumonitis, nephritis, fever, allergic reaction, anaphylaxis, dyspnea, or increased blood bilirubin or grade 4 hyperthyroidism, hypophysitis or diarrhea, which are considered possibly, probably, or definitely related to treatment, any time during treatment enrollment will be temporarily closed.

Any time after the first 6 patients have been enrolled, if 30% or more of the patient enrolled develop one (or more) of the toxicities listed in the table below during treatment or within 30 days of discontinuing protocol treatment and the toxicity is considered to be possibly, probably, or definitely related to treatment, enrollment will be temporarily closed.

| **Toxicity** | **Severity/duration** |
| --- | --- |
| Neurologic | Grade 2 and not resolved to ≤ Grade 1 within 14 days  ≥ Grade 3 regardless of time to resolution |
| Hematologic | Grade 4 and not resolved to ≤ Grade 1 within 14 days  Grade 3 with Grade 3+ febrile neutropenia or hemorrhage, regardless of time to resolution |
| Other | Grade 3 and not resolved to ≤ Grade 1 within 14 days  Grade 4 regardless of time to resolution  Any toxicity that leads to treatment discontinuation due to toxicity |

- 1. Inclusion of Women and Minorities

| **Accrual Targets** | | | |
| --- | --- | --- | --- |
| **Ethnic Category** | **Sex/Gender** | | |
|  | **Females** | **Males** | **Total** |
| Hispanic or Latino | 0 | 1 | 1 |
| Not Hispanic or Latino | 10 | 12 | 22 |
| **Ethnic Category: Total of all subjects*** | **10** | **13** | **23** |
| **Racial Category** | | | |
| American Indian or Alaskan Native |  |  |  |
| Asian |  |  |  |
| Black or African American |  |  |  |
| Native Hawaiian or other Pacific Islander |  |  |  |
| White | 10 | 13 | 23 |
| **Racial Category: Total of all subjects*** | **10** | **13** | **23** |

| **Ethnic Categories:** | **Hispanic or Latino –** a person of Cuban, Mexican, Puerto Rican, South or Central American, or other Spanish culture or origin, regardless of race. The term “Spanish origin” can also be used in addition to “Hispanic or Latino.”  **Not Hispanic or Latino** |
| --- | --- |
| **Racial Categories:** | **American Indian or Alaskan Native –** a person having origins in any of the original peoples of North, Central, or South America, and who maintains tribal affiliations or community attachment.  **Asian –** a person having origins in any of the original peoples of the Far East, Southeast Asia, or the Indian subcontinent including, for example, Cambodia, China, India, Japan, Korea, Malaysia, Pakistan, the Philippine Islands, Thailand, and Vietnam. (Note: Individuals from the Philippine Islands have been recorded as Pacific Islanders in previous data collection strategies.)  **Black or African American –** a person having origins in any of the black racial groups of Africa.  **Native Hawaiian or other Pacific Islander –** a person having origins in any of the original peoples of Hawaii, Guam, Samoa, or other Pacific Islands.  **White –** a person having origins in any of the original peoples of Europe, the Middle East, or North Africa. |

MC1577 66 MCCC Amendment 8

# Pathology Considerations/Tissue Biospecimens

- 1. Summary Table of Research Tissue Specimens to be collected for this protocol

| **Correlative Study** | **Mandatory or Optional** | **Type of Tissue to Collect** | **Block, Slides, Core, etc. (# of**  **each to submit)** | **After consenting to pre- registration**  **phase** | **At most 21 days after Cycle 4**  **end date** | **At Confirmed**  **Progression** | **Process at site? (Yes**  **or No)** | **Temperature Conditions for Storage**  **/Shipping** |
| --- | --- | --- | --- | --- | --- | --- | --- | --- |
| Tumor Biopsy for immunological and biomarker studies | Mandatory^1^ | Formalin Fixed Paraffin (2 cores). Fresh frozen tumor  biopsy (1 core) | FFPE Block or 10 unstained FFPE slides (5 micron),  2 unstained FFPE  slides (10  micron), 1 FFPE slide stained with hematoxylin and eosin | X | --- | --- | Yes | FFPE:  Ambient Frozen: Liquid nitrogen or -  80˚C |
|  | Mandatory | Formalin Fixed Paraffin |  | --- | X | --- | Yes | Ambient |
|  | Optional | Formalin Fixed Paraffin |  | --- | --- | X | Yes | Ambient |

1. Unless tissue from a biopsy obtained ≤90 days prior to pre-registration is available for research use.

- 1. Paraffin Embedded Tissue
  2. Submit one formalin fixed paraffin-embedded (FFPE) tumor tissue block with largest amount of invasive tumor (at least 1 cm of tumor for cases of surgical resection) from metastatic tumor biopsy.
  3. If a specimen is to be submitted the following materials below are mandatory (unless indicated otherwise) and required for shipment:
     - Paraffin embedded tissue blocks with corresponding H&E slide (OR 10 unstained slides with corresponding H&E).
     - Specimen Submission:Tissue (Research) Form
     - Surgical Pathology Report
     - Operative Report (optional)
  4. Study Methodology and Storage Information
  5. FFPE tumor tissue blocks/slides (10 unstained slides) will be collected in order to assess correlation of tumor bound PD-L1 expression and level of tumor infiltrating T-cells (CD3, CD4, and CD8) in response to treatment. Both measurements will be semi-quantitatively assessed by established immunohistochemistry (IHC) methodology in Dr. Matthew Block’s laboratory (GU 3-26).
  6. Definitive immunohistochemical analysis using optional tumor biopsies will be performed at the time of confirmed progression, comparing pre-treatment measurements with changes at time of tumor progression on therapy.
  7. Unused material

At the completion of the study, any unused/remaining material will be stored in the Block laboratory for future research according to the patient consent permission (see Section 6.15). Potential future research may include immunohistochemistry (IHC) analyses to analyze predictive biomarkers, changes in expression pattern with therapy, and correlation with response and/or adverse events. When a protocol is developed, it will be presented for IRB review and approval.

- 1. Banking

Banking of tumor tissue, according to the patient consent permission (see Section 6.15), is for future research. As protocols are developed, they will be present for IRB review and approval.

Blocks requested to accommodate individual patient management will be returned promptly upon request.

Protocol Version: 16Sep2022

# Records and Data Collection Procedures

- 1. Submission Timetable

Data submission instructions for this study can be found in the Data Submission Schedule.

- 1. Event monitoring

See Section 4.0 and data submission table for the event monitoring schedule.

- 1. CRF completion

This study will use Medidata Rave for remote data capture (rdc) of all study data.

- 1. Site responsibilities

Each site will be responsible for insuring that all materials contain the patient’s initials, MCCC registration number, and MCCC protocol number. Patient’s name must be removed.

- 1. Supporting documentation

This study requires supporting documentation for evidence of response to study therapy and progression after study therapy (CR, PR, PD).

- 1. Labelling of materials

Each site will be responsible for insuring that all materials contain the patient’s initials, MCCC registration number, and MCCC protocol number. Patient’s name must be removed.

- 1. Incomplete materials

Any materials deemed incomplete by the MCCC Operations Office will be considered “not received” and will not be edited or otherwise processed until the missing information is received. A list of the missing documents will be made available to the appropriate co-sponsor/participant.

- 1. Overdue lists

A list of overdue materials and forms for study patients will be generated monthly. The listings will be sorted by location and will include the patient study registration number. The appropriate co-sponsor/participant will be responsible to obtain the overdue material.

- 1. Corrections forms

If a correction is necessary the QAS will query the site. The query will be sent to the appropriate site to make the correction and return the query and documentation of correction back to the QAS.

# Budget

- 1. Costs charged to patient: routine clinical care, including pembrolizumab, provider visits, CBC with differential, Chemistry Group, TSH, and tumor imaging.
  2. Tests to be research funded: HLA class I typing, tumor biopsies, and research blood tests.

# References

- - 1. PD-1 Inhibitors Effective in Hodgkin Lymphoma. *Cancer discovery* **5**, 102-103 (2015).
    2. Ishida, Y., Agata, Y., Shibahara, K. & Honjo, T. Induced expression of PD-1, a novel member of the immunoglobulin gene superfamily, upon programmed cell death. *The EMBO journal* **11**, 3887-3895 (1992).
    3. Nishimura, H.*, et al.* Autoimmune dilated cardiomyopathy in PD-1 receptor-deficient mice.

*Science* **291**, 319-322 (2001).

- - 1. Nishimura, H., Nose, M., Hiai, H., Minato, N. & Honjo, T. Development of lupus-like autoimmune diseases by disruption of the PD-1 gene encoding an ITIM motif-carrying immunoreceptor. *Immunity* **11**, 141-151 (1999).
    2. Dong, H.*, et al.* Tumor-associated B7-H1 promotes T-cell apoptosis: a potential mechanism of immune evasion. *Nature medicine* **8**, 793-800 (2002).
    3. Zou, W. & Chen, L. Inhibitory B7-family molecules in the tumour microenvironment. *Nature reviews. Immunology* **8**, 467-477 (2008).
    4. Hodi, F.S.*, et al.* Improved survival with ipilimumab in patients with metastatic melanoma. *N* *Engl J Med* **363**, 711-723 (2010).
    5. Robert, C.*, et al.* Anti-programmed-death-receptor-1 treatment with pembrolizumab in ipilimumab-refractory advanced melanoma: a randomised dose-comparison cohort of a phase 1 trial. *Lancet* **384**, 1109-1117 (2014).
    6. Pardoll, D.M. The blockade of immune checkpoints in cancer immunotherapy. *Nature reviews. Cancer* **12**, 252-264 (2012).
    7. Tumeh, P.C.*, et al.* PD-1 blockade induces responses by inhibiting adaptive immune resistance. *Nature* **515**, 568-571 (2014).
    8. Bose, A., Chakraborty, T., Chakraborty, K., Pal, S. & Baral, R. Dysregulation in immune functions is reflected in tumor cell cytotoxicity by peripheral blood mononuclear cells from head and neck squamous cell carcinoma patients. *Cancer immunity* **8**, 10 (2008).
    9. Gajewski, T.F.*, et al.* Immune resistance orchestrated by the tumor microenvironment.

*Immunol Rev* **213**, 131-145 (2006).

- - 1. Tatsumi, T.*, et al.* Disease-associated bias in T helper type 1 (Th1)/Th2 CD4(+) T cell responses against MAGE-6 in HLA-DRB10401(+) patients with renal cell carcinoma or melanoma. *J Exp Med* **196**, 619-628 (2002).
    2. Terheyden, P.*, et al.* Longitudinal analysis of MART-1/HLA-A2-reactive T cells over the course of melanoma progression. *Scand J Immunol* **58**, 566-571 (2003).
    3. Nevala, W.K.*, et al.* Evidence of systemic Th2-driven chronic inflammation in patients with metastatic melanoma. *Clinical cancer research : an official journal of the American* *Association for Cancer Research* **15**, 1931-1939 (2009).
    4. Honigberg, L.A.*, et al.* The Bruton tyrosine kinase inhibitor PCI-32765 blocks B-cell activation and is efficacious in models of autoimmune disease and B-cell malignancy. *Proceedings of the National Academy of Sciences of the United States of America* **107**, 13075-13080 (2010).
    5. Herman, S.E.*, et al.* Bruton tyrosine kinase represents a promising therapeutic target for treatment of chronic lymphocytic leukemia and is effectively targeted by PCI-32765. *Blood* **117**, 6287-6296 (2011).
    6. Ponader, S.*, et al.* The Bruton tyrosine kinase inhibitor PCI-32765 thwarts chronic lymphocytic leukemia cell survival and tissue homing in vitro and in vivo. *Blood* **119**, 1182- 1189 (2012).
    7. Advani, R.H.*, et al.* Bruton tyrosine kinase inhibitor ibrutinib (PCI-32765) has significant activity in patients with relapsed/refractory B-cell malignancies. *Journal of clinical oncology*

*: official journal of the American Society of Clinical Oncology* **31**, 88-94 (2013).

- - 1. Byrd, J.C.*, et al.* Targeting BTK with ibrutinib in relapsed chronic lymphocytic leukemia.

*The New England journal of medicine* **369**, 32-42 (2013).

- - 1. Byrd, J.C.*, et al.* Ibrutinib versus ofatumumab in previously treated chronic lymphoid leukemia. *The New England journal of medicine* **371**, 213-223 (2014).
    2. Wang, M.L.*, et al.* Targeting BTK with ibrutinib in relapsed or refractory mantle-cell lymphoma. *The New England journal of medicine* **369**, 507-516 (2013).
    3. Cameron, F. & Sanford, M. Ibrutinib: first global approval. *Drugs* **74**, 263-271 (2014).
    4. Treon, S.P.*, et al.* Ibrutinib in Previously Treated Waldenstrom's Macroglobulinemia. *New Engl J Med* **372**, 1430-1440 (2015).
    5. Bunt, S.K.*, et al.* Reduced inflammation in the tumor microenvironment delays the accumulation of myeloid-derived suppressor cells and limits tumor progression. *Cancer Res* **67**, 10019-10026 (2007).
    6. Haabeth, O.A.*, et al.* Inflammation driven by tumour-specific Th1 cells protects against B- cell cancer. *Nat Commun* **2**, 240 (2011).
    7. Dubovsky, J.A.*, et al.* Ibrutinib is an irreversible molecular inhibitor of ITK driving a Th1- selective pressure in T lymphocytes. *Blood* **122**, 2539-2549 (2013).
    8. Dubovsky, J.A.*, et al.* Ibrutinib treatment ameliorates murine chronic graft-versus-host disease. *The Journal of clinical investigation* **124**, 4867-4876 (2014).
    9. [Sagiv-Barfi I,](https://www.ncbi.nlm.nih.gov/pubmed/?term=Sagiv-Barfi%20I%5BAuthor%5D&cauthor=true&cauthor_uid=25730880) [Kohrt HE,](https://www.ncbi.nlm.nih.gov/pubmed/?term=Kohrt%20HE%5BAuthor%5D&cauthor=true&cauthor_uid=25730880) [Czerwinski DK,](https://www.ncbi.nlm.nih.gov/pubmed/?term=Czerwinski%20DK%5BAuthor%5D&cauthor=true&cauthor_uid=25730880) [Ng PP,](https://www.ncbi.nlm.nih.gov/pubmed/?term=Ng%20PP%5BAuthor%5D&cauthor=true&cauthor_uid=25730880) [Chang BY,](https://www.ncbi.nlm.nih.gov/pubmed/?term=Chang%20BY%5BAuthor%5D&cauthor=true&cauthor_uid=25730880) [Levy R.](https://www.ncbi.nlm.nih.gov/pubmed/?term=Levy%20R%5BAuthor%5D&cauthor=true&cauthor_uid=25730880) Therapeutic antitumor immunity by checkpoint blockade is enhanced by ibrutinib, an inhibitor of both BTK and ITK. Proc Natl Acad Sci U S A. 2015 Mar 3;112(9):E966-72. doi: 10.1073/pnas.1500712112.

Epub 2015 Feb 17.

- - 1. Gehan EA. The determination of number of patients in a follow-up trial of a new chemotherapeutic agent. J. Chronic. Dis. 1961;13:346-353.

# Appendix I - MC1577 Patient Medication Diary

**Cycle 1 Only**

**Name _ Study ID Number**

Please complete this diary on a daily basis. Write in the amount of the dose of ibrutinib that you took in the appropriate “Day” box.

On the days that you do not take any study drug, please write in “0”. If you forget to take your daily dose, please write in “0”, but remember to take your prescribed dose at the next regularly scheduled time.

Week of:

| ***Study Drug*** | ***Day 1*** | ***Day 2*** | ***Day 3*** | ***Day 4*** | ***Day 5*** | ***Day 6*** | ***Day 7*** |
| --- | --- | --- | --- | --- | --- | --- | --- |
| ibrutinib |  |  |  |  |  |  |  |
|  |  |  |  |  |  |  |  |
|  |  |  |  |  |  |  |  |

Week of:

| ***Study Drug*** | ***Day 8*** | ***Day 9*** | ***Day 10*** | ***Day 11*** | ***Day 12*** | ***Day 13*** | ***Day 14*** |
| --- | --- | --- | --- | --- | --- | --- | --- |
| ibrutinib |  |  |  |  |  |  |  |
|  |  |  |  |  |  |  |  |
|  |  |  |  |  |  |  |  |

Week of:

| ***Study Drug*** | ***Day 15*** | ***Day 16*** | ***Day 17*** | ***Day 18*** | ***Day 19*** | ***Day 20*** | ***Day 21*** |
| --- | --- | --- | --- | --- | --- | --- | --- |
| ibrutinib |  |  |  |  |  |  |  |
|  |  |  |  |  |  |  |  |
|  |  |  |  |  |  |  |  |

Week of:

| ***Study Drug*** | ***Day 22*** | ***Day 23*** | ***Day 24*** | ***Day 25*** | ***Day 26*** | ***Day 27*** | ***Day 28*** |
| --- | --- | --- | --- | --- | --- | --- | --- |
| ibrutinib |  |  |  |  |  |  |  |
|  |  |  |  |  |  |  |  |
|  |  |  |  |  |  |  |  |

Week of:

| ***Study Drug*** | ***Day 29*** | ***Day 30*** | ***Day 31*** |
| --- | --- | --- | --- |
| ibrutinib |  |  |  |
|  |  |  |  |
|  |  |  |  |

Patient signature:

**My next scheduled visit is:**

If you have any questions, please call:

**Study Coordinator Use Only**

**Verified by**

**Date**

**MC1577 Patient Medication Diary – Cycle 2 and subsequent cycles**

**Name _ Study ID Number**

Please complete this diary on a daily basis. Write in the amount of the dose of that you took in the appropriate “Day” box.

On the days that you do not take any study drug, please write in “0”. If you forget to take your daily dose, please write in “0”, but remember to take your prescribed dose at the next regularly scheduled time.

Week of:

| ***Study Drug*** | ***Day 1*** | ***Day 2*** | ***Day 3*** | ***Day 4*** | ***Day 5*** | ***Day 6*** | ***Day 7*** |
| --- | --- | --- | --- | --- | --- | --- | --- |
|  |  |  |  |  |  |  |  |
|  |  |  |  |  |  |  |  |
|  |  |  |  |  |  |  |  |

Week of:

| ***Study Drug*** | ***Day 8*** | ***Day 9*** | ***Day 10*** | ***Day 11*** | ***Day 12*** | ***Day 13*** | ***Day 14*** |
| --- | --- | --- | --- | --- | --- | --- | --- |
|  |  |  |  |  |  |  |  |
|  |  |  |  |  |  |  |  |
|  |  |  |  |  |  |  |  |

Week of:

| ***Study Drug*** | ***Day 15*** | ***Day 16*** | ***Day 17*** | ***Day 18*** | ***Day 19*** | ***Day 20*** | ***Day 21*** |
| --- | --- | --- | --- | --- | --- | --- | --- |
|  |  |  |  |  |  |  |  |
|  |  |  |  |  |  |  |  |
|  |  |  |  |  |  |  |  |

Week of:

| ***Study Drug*** | ***Day 22*** | ***Day 23*** | ***Day 24*** |
| --- | --- | --- | --- |
|  |  |  |  |
|  |  |  |  |
|  |  |  |  |

Patient signature:

**My next scheduled visit is:**

If you have any questions, please call:

**Study Coordinator Use Only**

**Verified by**

**Date**

# Appendix II - ECOG Performance Status

| **ECOG PERFORMANCE STATUS*** | |
| --- | --- |
| **Grade** | **ECOG** |
| 0 | Fully active, able to carry on all pre-disease performance without restriction |
| 1 | Restricted in physically strenuous activity but ambulatory and able to carry out work of a light or sedentary nature, e.g., light house work, office work |
| 2 | Ambulatory and capable of all selfcare but unable to carry out any work activities. Up and about more than 50% of waking hours |
| 3 | Capable of only limited selfcare, confined to bed or chair more than 50% of waking hours. |
| 4 | Completely disabled. Cannot carry on any selfcare. Totally confined to bed or chair. |
| 5 | Dead |

*As published in Am. J. Clin. Oncol.:

*Oken, M.M., Creech, R.H., Tormey, D.C., Horton, J., Davis, T.E., McFadden, E.T., Carbone, P.P.: Toxicity And Response Criteria Of The Eastern Cooperative Oncology Group. Am J Clin Oncol 5:649- 655, 1982.*

The ECOG Performance Status is in the public domain therefore available for public use. To duplicate the scale, please cite the reference above and credit the Eastern Cooperative Oncology Group, Robert Comis M.D., Group Chair.

From <http://www.ecog.org/general/perf_stat.html>

# Appendix III - CTCAE

**Common Terminology Criteria for Adverse Events v4.0 (CTCAE)**

The descriptions and grading scales found in the revised NCI Common Terminology Criteria for Adverse Events (CTCAE) version 4.0 will be utilized for adverse event reporting. (<http://ctep.cancer.gov/reporting/ctc.html>)

# Appendix IV - Potential Drug-Drug Interactions for Ibrutinib

Ibrutinib is primarily metabolized by cytochrome P450 enzyme 3A.

**Agents That May Increase ibrutinib Plasma Concentrations (CYP3A Inhibitors)** Concomitant use of ibrutinib and drugs that strongly or moderately inhibit CYP3A can increase ibrutinib exposure and therefore strong CYP3A inhibitors should be avoided.

- If a strong CYP3A inhibitor (eg, ketoconazole, indinavir, nelfinavir, ritonavir, saquinavir, clarithromycin, telithromycin, itraconazole, nefazodone, cobicistat, and posaconazole) must be used, reduce ibrutinib to 140 mg for the duration of the inhibitor use or withhold ibrutinib treatment temporarily (for 7 days or less). Subjects should be monitored for signs of ibrutinib toxicity.
- If a moderate CYP3A inhibitor (eg, fluconazole, voriconazole, erythromycin, amprenavir, aprepitant, atazanavir, ciprofloxacin, crizotinib, diltiazem, fosamprenavir, imatinib, verapamil, amiodarone, and dronedarone) is indicated, reduce ibrutinib dose to 140 mg for the duration of the inhibitor use.
- No dose adjustment is required in combination with mild inhibitors.
- Avoid grapefruit and Seville oranges during ibrutinib treatment as these contain moderate inhibitors of CYP3A (see Section 5.3.1.2).

Avoid concomitant use of systemic strong CYP3A inducers (eg, carbamazepine, rifampin, phenytoin, and St. John’s Wort). Consider alternative agents with less CYP3A induction.

A list of common CYP3A inhibitors and inducers is provided in Appendix V. For further information, please refer to the current version of the IB and examples of inhibitors, inducers, and substrates can be found at [http://medicine.iupui.edu/clinpharm/ddis/main-table/.](http://medicine.iupui.edu/clinpharm/ddis/main-table/) This website is continually revised and should be checked frequently for updates.

Co-administration of ketoconazole, a strong CYP3A inhibitor, in 18 healthy subjects, increased exposure (Cmax and AUClast) of ibrutinib by 29- and 24-fold, respectively. The maximal observed ibrutinib exposure (AUC) was ≤ 2-fold in 37 subjects treated with mild and/or moderate CYP3A inhibitors when compared with the ibrutinib exposure in 76 subjects not treated concomitantly with CYP3A inhibitors. Clinical safety data in 66 subjects treated with moderate (n=47) or strong CYP3A inhibitors (n=19) did not reveal meaningful increases in toxicities. Strong inhibitors of CYP3A (e.g., ketoconazole, voriconazole, posaconazole, indinavir, nelfinavir, ritonavir, saquinavir, clarithromycin, telithromycin, itraconazole, nefazadone and cobicistat) and moderate inhibitors (e.g., erythromycin, amprenavir, aprepitant, atazanavir, ciprofloxacin, crizotinib, darunavir/ritonavir, diltiazem, fluconazole, fosamprenavir, imatinib, verapamil) should be avoided (See Section 8.4).

**Agents That May Decrease ibrutinib Plasma Concentrations (CYP3A Inducers)** Administration of ibrutinib with rifampin, a strong CYP3A inducer, decreases ibrutinib plasma concentrations by approximately 90%. Avoid concomitant use of strong CYP3A inducers (eg, carbamazepine, rifampin, phenytoin and St. John’s Wort). Consider alternative agents with less CYP3A induction.

**Physiologically-based pharmacokinetic guided DDI simulations**

Physiologically-based pharmacokinetic (PBPK) guided DDI simulations were performed for 11 CYP3A inhibitors and inducers. Simulated interactions for coadministered ketoconazole and rifampin, a strong inhibitor and inducer, respectively, were in line with the observed data, thus validating the usefulness of the PBPK model for predicting CYP3A-mediated drug interactions.

Simulations were then performed for the inhibitors clarithromycin, voriconazole, erythromycin, diltiazem, grapefruit juice, fluvoxamine and azithromycin, as well as the inducers carbamazepine and efavirenz. Clarithromycin, a strong inhibitor, administered at 500 mg bid may cause an ibrutinib AUC increase of 14-fold under fasted conditions. Moderate and strong CYP3A inducers efavirenz and carbamazepine may decrease the AUC of ibrutinib by 2.5-, and 5.6- fold, respectively.

**Drugs that may have their plasma concentrations altered by ibrutinib**

In vitro studies indicated that ibrutinib is a weak inhibitor toward CYP2B6, CYP2C8, CYP2C9, CYP2C19, CYP2D6, and CYP3A4/5. The dihydrodiol metabolite of ibrutinib is a weak inhibitor toward CYP2B6, CYP2C8, CYP2C9, and CYP2D6. Both ibrutinib and the dihydrodiol metabolite are at most weak inducers of CYP450 isoenzymes in vitro. Therefore, it is unlikely that ibrutinib has any clinically relevant drug-drug interactions with drugs that may be metabolized by the CYP450 enzymes.

In vitro studies indicated that ibrutinib is not a substrate of P-gp, but is a mild inhibitor. Ibrutinib is not expected to have systemic drug-drug interactions with P-gp substrates. However, it cannot be excluded that ibrutinib could inhibit intestinal P-gp and BCRP after a therapeutic dose. There are no clinical data available. To avoid a potential interaction in the GI tract, narrow therapeutic range P-gp substrates such as digoxin or methotrexate should be taken at least 6 hours before or after ibrutinib.

# Appendix V - Inhibitors and Inducers of CYP3A

Inhibitors of CYP3A are defined as follows. A comprehensive list of inhibitors can be found at the following website: <http://medicine.iupui.edu/clinpharm/ddis/table.aspx> . The general categorization into strong, moderate, and weak inhibitors according to the website is displayed below. Refer to Section 8.1 on instructions for concomitant use of CYP3A inhibitors and inducers with ibrutinib.

| **Inhibitors of CYP3A** | **Inducers of CYP3A** |
| --- | --- |
| **Strong inhibitors:** | Carbamazepine |
| INDINAVIR | Efavirenz |
| NELFINAVIR | Nevirapine |
| RITONAVIR | Barbiturates |
| CLARITHROMYCIN | Glucocorticoids |
| ITRACONAZOLE | Modafinil |
| KETOCONAZOLE | Oxcarbarzepine |
| NEFAZODONE | Phenobarbital |
| SAQUINAVIR | Phenytoin |
| SUBOXONE | Pioglitazone |
| TELITHROMYCIN | Rifabutin |
| VORICONAZOLE | Rifampin |
| POSACONAZOLE | St. John’s Wort |
|  | Troglitazone |
| **Moderate inhibitors:** |  |
| Aprepitant |  |
| Erythromycin |  |
| diltiazem |  |
| Fluconazole |  |
| grapefruit juice |  |
| Seville orange juice |  |
| Verapamil |  |
| **Weak inhibitors:** |  |
| Cimetidine |  |
| **All other inhibitors:** |  |
| Amiodarone |  |
| NOT azithromycin |  |
| Chloramphenicol |  |
| Boceprevir |  |
| Ciprofloxacin |  |
| Delaviridine |  |
| diethyl-dithiocarbamate |  |
| Fluvoxamine |  |
| Gestodene |  |
| Imatinib |  |
| Mibefradil |  |
| Mifepristone |  |
| Norfloxacin |  |
| Norfluoxetine |  |
| star fruit |  |
| Telaprevir |  |
| Troleandomycin |  |

Source:[http://medicine.iupui.edu/clinpharm/ddis/table.aspx.](http://medicine.iupui.edu/clinpharm/ddis/table.aspx)

# Appendix VI - Expected Adverse Reactions of Ibrutinib

| **IB Edition 10 study pool = 17 studies Data cut off Date 31 May 2016** | **All Grades Frequency n (%)**  **N = 1741** | **Grade ≥ 3 Frequency n (%)**  **N = 1741** | **Serious Frequency n (%)**  **N = 1741** |
| --- | --- | --- | --- |
| **SERIOUS EXPECTED TERMS ↓** |  |  |  |
| **System Organ Class**  Preferred Term |  |  |  |
| **Blood and lymphatic system disorders** |  |  |  |
| Anaemia+ | 338 (19.41) | 88 (5.05) | 18 (1.03) |
| Febrile neutropenia#* | 83 (4.77) | 80 (4.60) | 68 (3.91) |
| Leukocytosis# | 41 (2.35) | 27 (1.55) | 4 (0.23) |
| Leukostasis syndrome# | 2 (0.11) | 2 (0.11) | 1 (0.06) |
| Lymphadenitis+ | 8 (0.46) | 4 (0.23) | 3 (0.17) |
| Lymphocytosis# | 23 (1.32) | 10 (0.57) | 2 (0.11) |
| Neutropenia# | 453 (26.02) | 387 (22.23) | 15 (0.86) |
| Pancytopenia+ | 4 (0.23) | 4 (0.23) | 3 (0.17) |
| Thrombocytopenia# | 319 (18.32) | 146 (8.39) | 15 (0.86) |
| **Cardiac disorders** |  |  |  |
| Atrial fibrillation#* | 111 (6.38) | 51 (2.93) | 54 (3.10) |
| Atrial flutter+ | 9 (0.52) | 2 (0.11) | 5 (0.29) |
| Cardiac failure congestive+ | 5 (0.29) | 4 (0.23) | 3 (0.17) |
| Myocardial infarction+* | 7 (0.40) | 6 (0.34) | 6 (0.34) |
| **Eye disorders** |  |  |  |
| Vision blurred# | 101 (5.80) | 1 (0.06) | 1 (0.06) |
| **Gastrointestinal disorders** |  |  |  |
| Colitis+ | 12 (0.69) | 5 (0.29) | 5 (0.29) |
| Constipation# | 276 (15.85) | 4 (0.23) | 1 (0.06) |
| Diarrhoea# | 735 (42.22) | 56 (3.22) | 16 (0.92) |
| Gastritis+ | 27 (1.55) | 4 (0.23) | 5 (0.29) |
| Intestinal obstruction+ | 2 (0.11) | 2 (0.11) | 2 (0.11) |
| Nausea# | 467 (26.82) | 11 (0.63) | 8 (0.46) |
| Oral mucosal blistering# | 8 (0.46) | 1 (0.06) | 1 (0.06) |
| Stomatitis# | 167 (9.59) | 12 (0.69) | 3 (0.17) |
| Vomiting# | 252 (14.47) | 10 (0.57) | 7 (0.40) |
| **General disorders and administration site conditions** |  |  |  |
| Asthenia+ | 109 (6.26) | 14 (0.80) | 5 (0.29) |
| Fatigue+ | 507 (29.12) | 49 (2.81) | 8 (0.46) |
| Oedema peripheral# | 258 (14.82) | 11 (0.63) | 7 (0.40) |
| Pyrexia# | 339 (19.47) | 30 (1.72) | 39 (2.24) |
| Systemic inflammatory response syndrome+* | 3 (0.17) | 3 (0.17) | 2 (0.11) |
| **Infections and infestations** |  |  |  |
| Atypical pneumonia# | 5 (0.29) | 1 (0.06) | 2 (0.11) |
| Bacteraemia# | 12 (0.69) | 10 (0.57) | 8 (0.46) |
| Bacteroides bacteraemia# | 1 (0.06) | 1 (0.06) | 1 (0.06) |
| Breast cellulitis# | 2 (0.11) | 0 | 1 (0.06) |
| Bronchitis+ | 98 (5.63) | 19 (1.09) | 12 (0.69) |
| Bronchopulmonary aspergillosis# | 3 (0.17) | 3 (0.17) | 3 (0.17) |

**+ Expected serious adverse events (SAEs).** Criteria for inclusion: 2 or more related serious events reported

**# Expected serious Adverse Drug Reactions (ADRs).** Criteria for inclusion: 1 or more serious ADRs reported

*** Fatal outcome reported.** Fatal events and ADRs are not expected unless marked with an asterisk

**Reference Safety Information (RSI) 2**

**Serious Expected Terms Associated with Ibrutinib Coded by MedDRA (version 19.0)**

|  | **Overall Frequencies** | | |
| --- | --- | --- | --- |
| **IB edition 10.1 study pool = 17 studies Data cutoff date = 31-May-2016**  **SERIOUS EXPECTED TERMS ↓** | **All Grades Frequency n (%)**  **N = 1741** | **Grade ≥ 3 Frequency n (%)**  **N = 1741** | **Serious Frequency n (%)**  **N = 1741** |
| **System Organ Class**  Preferred Term |  |  |  |
| Cellulitis orbital# | 1 (0.06) | 1 (0.06) | 1 (0.06) |
| Cellulitis staphylococcal# | 1 (0.06) | 1 (0.06) | 1 (0.06) |
| Cellulitis# | 81 (4.65) | 34 (1.95) | 28 (1.61) |
| Chronic sinusitis# | 13 (0.75) | 2 (0.11) | 2 (0.11) |
| Enterocolitis infectious+ | 4 (0.23) | 3 (0.17) | 2 (0.11) |
| Escherichia bacteraemia# | 1 (0.06) | 1 (0.06) | 1 (0.06) |
| Escherichia sepsis# | 2 (0.11) | 2 (0.11) | 2 (0.11) |
| Haemophilus bacteraemia# | 1 (0.06) | 1 (0.06) | 1 (0.06) |
| Haemophilus sepsis# | 1 (0.06) | 1 (0.06) | 1 (0.06) |
| Infection+ | 19 (1.09) | 5 (0.29) | 4 (0.23) |
| Lower respiratory tract infection viral# | 3 (0.17) | 2 (0.11) | 2 (0.11) |
| Lower respiratory tract infection# | 29 (1.67) | 9 (0.52) | 10 (0.57) |
| Lung infection pseudomonal# | 1 (0.06) | 1 (0.06) | 1 (0.06) |
| Lung infection# | 35 (2.01) | 25 (1.44) | 19 (1.09) |
| Neutropenic sepsis#* | 5 (0.29) | 4 (0.23) | 4 (0.23) |
| Periorbital cellulitis# | 4 (0.23) | 2 (0.11) | 2 (0.11) |
| Pneumococcal sepsis# | 2 (0.11) | 2 (0.11) | 2 (0.11) |
| Pneumocystis jirovecii pneumonia#* | 6 (0.34) | 6 (0.34) | 6 (0.34) |
| Pneumonia bacterial# | 6 (0.34) | 5 (0.29) | 4 (0.23) |
| Pneumonia cryptococcal#* | 2 (0.11) | 1 (0.06) | 1 (0.06) |
| Pneumonia fungal# | 3 (0.17) | 2 (0.11) | 1 (0.06) |
| Pneumonia haemophilus# | 2 (0.11) | 1 (0.06) | 1 (0.06) |
| Pneumonia influenzal#* | 2 (0.11) | 2 (0.11) | 2 (0.11) |
| Pneumonia klebsiella# | 1 (0.06) | 1 (0.06) | 1 (0.06) |
| Pneumonia legionella# | 1 (0.06) | 1 (0.06) | 1 (0.06) |
| Pneumonia parainfluenzae viral# | 1 (0.06) | 1 (0.06) | 1 (0.06) |
| Pneumonia pseudomonal# | 2 (0.11) | 1 (0.06) | 1 (0.06) |
| Pneumonia streptococcal# | 1 (0.06) | 1 (0.06) | 1 (0.06) |
| Pneumonia viral# | 6 (0.34) | 2 (0.11) | 4 (0.23) |
| Pneumonia#* | 215 (12.35) | 149 (8.56) | 152 (8.73) |
| Rash pustular# | 10 (0.57) | 1 (0.06) | 1 (0.06) |
| Respiratory tract infection+ | 32 (1.84) | 7 (0.40) | 5 (0.29) |
| Sepsis#* | 35 (2.01) | 34 (1.95) | 31 (1.78) |
| Septic shock#* | 8 (0.46) | 8 (0.46) | 8 (0.46) |
| Sinusitis fungal# | 2 (0.11) | 2 (0.11) | 1 (0.06) |
| Sinusitis# | 164 (9.42) | 13 (0.75) | 6 (0.34) |
| Skin infection# | 37 (2.13) | 1 (0.06) | 2 (0.11) |
| Staphylococcal infection# | 12 (0.69) | 2 (0.11) | 3 (0.17) |
| Staphylococcal skin infection# | 5 (0.29) | 2 (0.11) | 2 (0.11) |
| Upper respiratory tract infection# | 315 (18.09) | 20 (1.15) | 7 (0.40) |
| Urinary tract infection# | 157 (9.02) | 34 (1.95) | 25 (1.44) |

**+ Expected serious adverse events (SAEs).** Criteria for inclusion: 2 or more related serious events reported

**# Expected serious Adverse Drug Reactions (ADRs).** Criteria for inclusion: 1 or more serious ADRs reported

*** Fatal outcome reported.** Fatal events and ADRs are not expected unless marked with an asterisk

**Reference Safety Information (RSI) 3**

**Serious Expected Terms Associated with Ibrutinib Coded by MedDRA (version 19.0)**

|  | **Overall Frequencies** | | |
| --- | --- | --- | --- |
| **IB edition 10.1 study pool = 17 studies Data cutoff date = 31-May-2016**  **SERIOUS EXPECTED TERMS ↓** | **All Grades Frequency n (%)**  **N = 1741** | **Grade ≥ 3 Frequency n (%)**  **N = 1741** | **Serious Frequency n (%)**  **N = 1741** |
| **System Organ Class**  Preferred Term |  |  |  |
| Urosepsis# | 4 (0.23) | 3 (0.17) | 3 (0.17) |
| **Injury, poisoning and procedural complications** |  |  |  |
| Post procedural haemorrhage+ | 17 (0.98) | 5 (0.29) | 5 (0.29) |
| Subdural haematoma#* | 11 (0.63) | 7 (0.40) | 9 (0.52) |
| **Investigations** |  |  |  |
| Neutrophil count decreased+ | 77 (4.42) | 56 (3.22) | 3 (0.17) |
| Platelet count decreased+ | 90 (5.17) | 24 (1.38) | 5 (0.29) |
| **Metabolism and nutrition disorders** |  |  |  |
| Decreased appetite+ | 213 (12.23) | 15 (0.86) | 2 (0.11) |
| Dehydration+ | 63 (3.62) | 24 (1.38) | 16 (0.92) |
| Hyperuricaemia# | 122 (7.01) | 34 (1.95) | 3 (0.17) |
| Tumour lysis syndrome# | 15 (0.86) | 15 (0.86) | 9 (0.52) |
| **Musculoskeletal and connective tissue disorders** |  |  |  |
| Arthralgia# | 245 (14.07) | 13 (0.75) | 2 (0.11) |
| Arthritis+ | 22 (1.26) | 2 (0.11) | 3 (0.17) |
| Back pain# | 177 (10.17) | 18 (1.03) | 5 (0.29) |
| Bone pain# | 38 (2.18) | 3 (0.17) | 1 (0.06) |
| Flank pain# | 19 (1.09) | 3 (0.17) | 2 (0.11) |
| Groin pain# | 10 (0.57) | 1 (0.06) | 1 (0.06) |
| Myalgia# | 155 (8.90) | 4 (0.23) | 2 (0.11) |
| Pain in extremity# | 138 (7.93) | 9 (0.52) | 2 (0.11) |
| **Neoplasms benign, malignant and unspecified (inclcysts and polyps)** |  |  |  |
| Basal cell carcinoma# | 54 (3.10) | 3 (0.17) | 9 (0.52) |
| Basosquamous carcinoma of skin# | 1 (0.06) | 0 | 1 (0.06) |
| Squamous cell carcinoma of skin# | 8 (0.46) | 1 (0.06) | 2 (0.11) |
| Squamous cell carcinoma# | 45 (2.58) | 9 (0.52) | 5 (0.29) |
| **Nervous system disorders** |  |  |  |
| Dizziness# | 159 (9.13) | 3 (0.17) | 2 (0.11) |
| Haemorrhage intracranial+ | 4 (0.23) | 1 (0.06) | 4 (0.23) |
| Headache# | 229 (13.15) | 12 (0.69) | 4 (0.23) |
| Syncope+ | 29 (1.67) | 19 (1.09) | 7 (0.40) |
| **Renal and urinary disorders** |  |  |  |
| Acute kidney injury+ | 32 (1.84) | 18 (1.03) | 16 (0.92) |
| Haematuria+ | 53 (3.04) | 3 (0.17) | 4 (0.23) |
| **Reproductive system and breast disorders** |  |  |  |
| Pelvic pain# | 6 (0.34) | 2 (0.11) | 1 (0.06) |
| **Respiratory, thoracic and mediastinal disorders** |  |  |  |
| Alveolitis allergic# | 1 (0.06) | 1 (0.06) | 1 (0.06) |
| Cough+ | 345 (19.82) | 2 (0.11) | 4 (0.23) |

**+ Expected serious adverse events (SAEs).** Criteria for inclusion: 2 or more related serious events reported

**# Expected serious Adverse Drug Reactions (ADRs).** Criteria for inclusion: 1 or more serious ADRs reported

*** Fatal outcome reported.** Fatal events and ADRs are not expected unless marked with an asterisk

**Reference Safety Information (RSI) 4**

**Serious Expected Terms Associated with Ibrutinib Coded by MedDRA (version 19.0)**

|  | **Overall Frequencies** | | |
| --- | --- | --- | --- |
| **IB edition 10.1 study pool = 17 studies Data cutoff date = 31-May-2016**  **SERIOUS EXPECTED TERMS ↓** | **All Grades Frequency n (%)**  **N = 1741** | **Grade ≥ 3 Frequency n (%)**  **N = 1741** | **Serious Frequency n (%)**  **N = 1741** |
| **System Organ Class**  Preferred Term |  |  |  |
| Dyspnoea+ | 192 (11.03) | 26 (1.49) | 21 (1.21) |
| Epistaxis# | 143 (8.21) | 1 (0.06) | 2 (0.11) |
| Interstitial lung disease# | 2 (0.11) | 0 | 1 (0.06) |
| Lung infiltration#* | 10 (0.57) | 1 (0.06) | 1 (0.06) |
| Organising pneumonia#* | 1 (0.06) | 1 (0.06) | 1 (0.06) |
| Pleural effusion+ | 57 (3.27) | 19 (1.09) | 17 (0.98) |
| Pneumonitis# | 11 (0.63) | 4 (0.23) | 4 (0.23) |
| Respiratory failure+* | 20 (1.15) | 19 (1.09) | 15 (0.86) |
| **Skin and subcutaneous tissue disorders** |  |  |  |
| Angioedema# | 4 (0.23) | 2 (0.11) | 1 (0.06) |
| Rash erythematous# | 54 (3.10) | 1 (0.06) | 1 (0.06) |
| Rash macular# | 27 (1.55) | 2 (0.11) | 1 (0.06) |
| Rash maculo-papular# | 90 (5.17) | 18 (1.03) | 2 (0.11) |
| Rash# | 211 (12.12) | 6 (0.34) | 1 (0.06) |
| Stevens-Johnson syndrome# | 1 (0.06) | 1 (0.06) | 1 (0.06) |
| **Vascular disorders** |  |  |  |
| Haematoma#* | 44 (2.53) | 2 (0.11) | 1 (0.06) |
| Hypertension# | 172 (9.88) | 63 (3.62) | 5 (0.29) |
| Hypertensive crisis# | 3 (0.17) | 3 (0.17) | 3 (0.17) |
| Hypotension+ | 64 (3.68) | 13 (0.75) | 7 (0.40) |

**+ Expected serious adverse events (SAEs).** Criteria for inclusion: 2 or more related serious events reported

**# Expected serious Adverse Drug Reactions (ADRs).** Criteria for inclusion: 1 or more serious ADRs reported

*** Fatal outcome reported.** Fatal events and ADRs are not expected unless marked with an asterisk

# Appendix VII - Child-Pugh Score

| **Measure** | **1 point** | **2 points** | **3 points** |
| --- | --- | --- | --- |
| Total bilirubin, μmol/L (mg/dL) | <34 (<2) | 34-50 (2-3) | >50 (>3) |
| Serum albumin, g/L (g/dL) | >35 (>3.5) | 28-35 (2.8-3.5) | <28 (<2.8) |
| PT INR | <1.7 | 1.71-2.30 | >2.30 |
| Ascites | None | Mild | Moderate to Severe |
| Hepatic encephalopathy | None | Grade I-II (or suppressed with medication) | Grade III-IV (or refractory) |

| **Points** | **Class** |
| --- | --- |
| 5-6 | A |
| 7-9 | B |
| 10-15 | C |

Source:

1. Child CG, Turcotte JG. “Surgery and portal hypertension”. In Child CG. *The liver and portal hypertension.*

Philadelphia:Saunders. 1964. pp. 50-64.

1. Pugh RN, Murray-Lyon IM, Dawson L, Pietroni MC, Williams R . “Transection of te oesophagus for bleeding oesophageal varices”. *The British journal of surgery*, 1973;60: 646-9.

# Appendix VIII – Ibrutinib Additional Safety Information from Pharmacyclics

*Combination Studies*

Integrated safety data from a total of 422 subjects with B-cell malignancies from 4 combination therapy studies that have completed primary analysis or final analysis included in the CSR as of 31 July 2017 are briefly summarized below. Therapies used in combination with ibrutinib in these studies, included BR (bendamustine and rituximab), FCR (fludarabine, cyclophosphamide, and rituximab), ofatumumab, and R-CHOP (rituximab, cyclophosphamide, doxorubicin, vincristine, and prednisone).

The most frequently reported TEAEs in subjects receiving ibrutinib in combination therapy (N = 422):

| Most frequently reported TEAEs > 10% ^a^ | Most frequently reported Grade 3 or 4 TEAEs > 5% ^a^ | Most frequently reported Serious TEAEs > 2% ^a^ |
| --- | --- | --- |
| Neutropenia | Neutropenia | Neutropenia |
| Diarrhea | Diarrhea | Diarrhea |
| Nausea | Nausea | Thrombocytopenia |
| Thrombocytopenia | Thrombocytopenia | Fatigue |
| Fatigue | Fatigue | Anemia |
| Anemia | Anemia | Pyrexia |
| Pyrexia | Pyrexia | Pneumonia |
| Infusion related reaction | Upper respiratory tract infection | Febrile neutropenia |
| Upper respiratory tract infection | Constipation | Atrial fibrillation |
| Constipation | Vomiting | Cellulitis |
| Vomiting | Rash | Hypertension |
| Rash | Headache | Hyperuricaemia |
| Headache | Pneumonia | Leukopenia |
| Cough | Decreased appetite | Neutrophil count decreased |
| Muscle spasms | Contusion | Tumor lysis syndrome |
| Pneumonia | Febrile neutropenia | Urinary tract infection |
| Oedema peripheral |  | White blood cell count decreased |
| Arthralgia | | |
| Decreased appetite | | |
| Contusion | | |
| Insomnia | | |
| Chills | | |
| Peripheral sensory neuropathy | | |
| Stomatitis | | |
| Febrile neutropenia | | |
| Abdominal pain | | |
| Back pain | | |
| Bronchitis | | |

*^a^ Source is Table 7 of IB (v11).*
